# Divergent Organomagnesium Reactivity of Rigid, Dinucleating Naphthyridine Ligands: Backbone Changes with Big Impact

**DOI:** 10.1021/acs.organomet.5c00070

**Published:** 2025-05-16

**Authors:** Errikos Kounalis, Marieke M. Broekman, Puck Uyttewaal, Uladzislava Dabranskaya, Martin Lutz, Daniël L. J. Broere

**Affiliations:** a Organic Chemistry and Catalysis, Institute for Sustainable and Circular Chemistry, Faculty of Science, 8125 Utrecht University, Universiteitsweg 99, Utrecht 3584 CG, The Netherlands; b Structural Biochemistry Bijvoet Centre for Biomolecular Research, Faculty of Science, Utrecht University, Universiteitsweg 99, Utrecht 3584 CG, The Netherlands

## Abstract

We report the synthesis and characterization of two naphthyridine-based ligands bearing pendant secondary amine and amide donors, respectively. We additionally report their deprotonation chemistry and reactivity with dialkylmagnesium and Grignard reagents. The Grignard reactions yield structurally distinct **L**Mg_2_Cl_2_·(THF)_*n*_ complexes, with the amide-based complex exhibiting reduced steric strain from the ligand around the Mg_2_Cl_2_ core. Comparison of the steric profiles of the **L**Mg_2_Cl_2_·(THF)_*n*_ complexes reveals that this reduced steric strain stems from the difference in binding modes of the ligands, which in the amide case points the bulk of sterically demanding substituents away from the Mg_2_Cl_2_ core. Reactivity of the ligands with Mg­(*n*-Bu)_2_ shows divergent outcomes: the secondary amine-based ligand forms the **L**Mg_2_(*n*-Bu)_2_·(THF)_2_ complex cleanly, whereas the amide-based ligand produces paramagnetic species via Mg–C homolysis, triggering radical reactivity that results in ligand butylation and dimerization. These findings underscore the unique steric and electronic features of dimagnesium complexes supported by rigid, dinucleating naphthyridine ligands, highlighting how variations in ligand architecture can profoundly influence coordination chemistry and reactivity.

## Graphical abstract

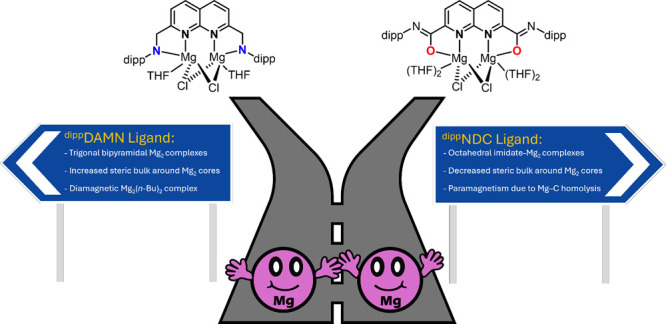

## Introduction

The high abundance of magnesium in the Earth’s crust, coupled with its low toxicity,^1^ has driven considerable interest in exploring the potential of Mg-based complexes for various applications. Extending beyond the well-established organomagnesium chemistry pioneered by Grignard,^2^ Mg complexes have demonstrated utility in diverse reactions, including hydroelementation,^3–8^ Lewis-acid catalysis,^9,10^ polymerization,^11^ and dehydrocoupling.^12^ These complexes are often stabilized by anionic diketiminate (NacNac) ligands, which employ hard nitrogen donors to support the Mg centers.^13^ The propensity for dimerization (resulting in a Mg_2_X_2_ “diamond core”) in such systems is well-documented, with a variety of bridging ligands, such as halides,^14–16^ hydrides,^17^ amidoboranes,^18^ and alkyl groups,^14,18^ forming dimers that mitigate the electronic and coordinative unsaturation of monomeric species. The nuclearity of Mg complexes, influenced by factors such as steric strain,^18,19^ the presence of coordinating solvents or coligands,^14,18,20–22^ and coligand size,^15^ significantly impacts their reactivity. For example, computational studies by the Maron and Hill groups on Mg-catalyzed hydrosilylation of alkenes have shown that dinuclear complexes can retain their nuclearity during alkene insertion, while subsequent steps involve monomeric species.^23^ The cooperative reactivity of these dinuclear complexes has been highlighted in work by the Harder group, where dimagnesium complexes based on dinucleating (bis-NacNac) ligands were shown to result in an alternative mechanistic pathway and decreased onset-temperatures for the dehydrocoupling of amidoborane complexes.^24^ Additionally, the Williams group has demonstrated that a dimagnesium complex bound to a macrocyclic ligand is highly active toward epoxide/CO_2_ ring-opening copolymerization (ROCOP).^25^ Despite these advantages, the flexibility of the ligands in these examples often allows the Mg centers to adopt variable distances, complicating efforts to study the intrinsic reactivity of the dimagnesium core.

To address this challenge and harness the advantages of enforced nuclearity, we aimed to synthesize dimagnesium complexes within a rigid, dinucleating framework. We chose the 1,8-naphthyridine motif as the basis for our ligands, as this framework provides a constrained environment to enforce close proximity between the Mg centers and suppresses monomer–dimer equilibria that could alter reactivity.^26^ Previous work has shown that the phosphine donors of the naphthyridine-based bis-phosphine ‘expanded pincer’ ligand (^*t* **‑Bu**^ **PNNP**) developed by our group can be a poor match for hard metals.^27^ As such, in this work, we introduce the Di-Amino-Methylene-Naphthyridine (^**dipp**^ **DAMN**) and Naphthyridine Di-Carboxylamide (^**dipp**^ **NDC**) ligands (Scheme 1c). Both ligands feature anionic flanking donors, making them more suitable for stabilizing dinuclear cores of harder metals such as Mg. We report the deprotonation chemistry of both ligands and detail the synthesis and characterization of various dimagnesium complexes supported by these ligands and quantify their steric profiles. These studies expand the library of dimagnesium complexes and offer insights into how nuclearity and ligand design influence magnesium-based coordination chemistry and reactivity, displaying markedly divergent behavior upon modifications in the ligand architecture.

**Scheme 1:**
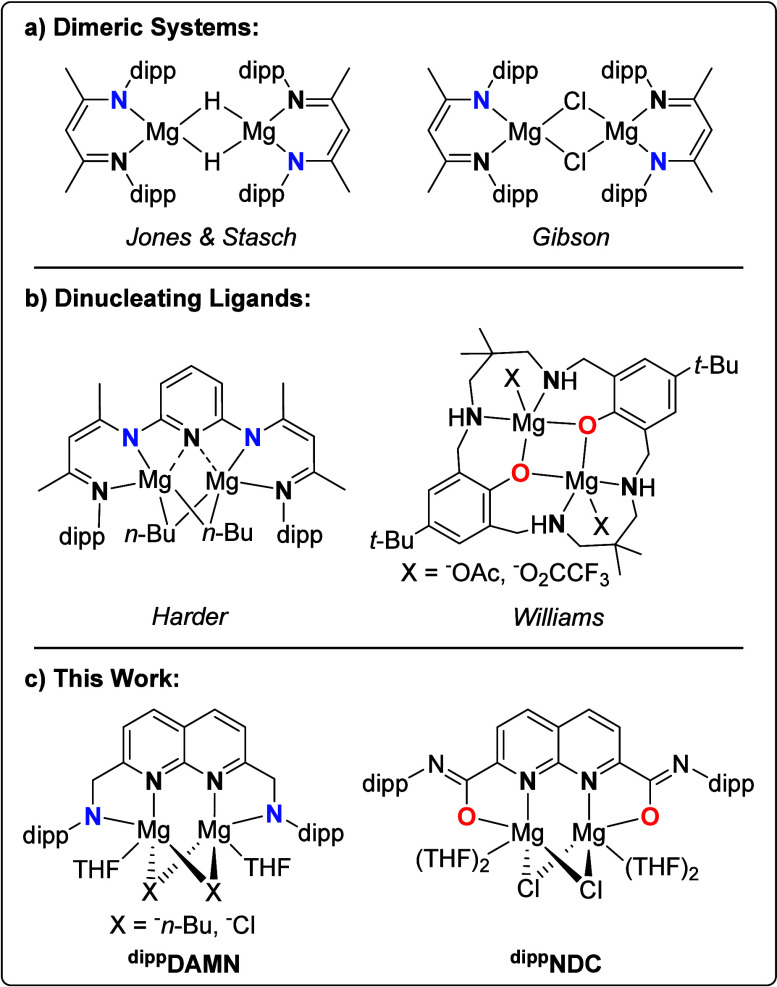
**(a)** Representative Dimeric Dimagnesium Complexes Reported in Literature,^15,17^ **(b)** Dimagnesium Complexes Supported by Dinucleating Ligands,^24,25^ and **(c)** the Dimagnesium Complexes Reported in This Work

## Results and Discussion

### Synthesis of ^dipp^DAMN and Its Deprotonation

The ^**dipp**^ **DAMN** ligand (Scheme 2) was synthesized as a slightly air-sensitive white solid in three steps from 2,7-dimethyl-1,8-naphthyridine in a 32% overall yield (see ESI Section 1.2). We envisioned that isolation of the dipotassium salt of the ligand would allow access to complexes of metals where internal base precursors are not available, similar as demonstrated for the related ^*t* **‑Bu**^ **PNNP** ligand.^28^ Accordingly, the ^**dipp**^ **DAMN** ligand was treated with two equiv of KO*t*-Bu in THF, which led to a dark orange solution. Analysis of the reaction mixture by ^1^H NMR (toluene-*d*_8_, 298 K) spectroscopy revealed broad resonances, which we attributed to fluxional binding of K^+^. Using VT NMR spectroscopy, no decoalescence was observed down to 193 K (toluene-*d*_8_, see ESI Figure S7). Notably, the addition of 1 equiv 18-crown-6 to this mixture resulted in a color change to dark green and the concomitant formation of **1**. The ^1^H and ^13^C­{^1^H}­NMR spectra (C_6_D_6_, 298 K) showed a number of resonances indicative of the loss of the C_2v_-symmetry (see ESI Section 1.4). The ^1^H NMR spectrum revealed four equally integrating naphthyridine resonances (δ = 6.34–5.78 ppm) at a more upfield shift compared to the free ligand, which agrees with partial dearomatization of the naphthyridine core. This is further confirmed by the observation of a singlet at δ = 5.46 ppm which we attribute to the methine proton. The presence of a broader apparent singlet at δ = 4.84 ppm (attributed to N–*H*) and a doublet (^3^ *J*_H,H_ = 4.8 Hz) at δ = 3.98 ppm (attributed to –C*H*_2_) integrating in a 1:2 ratio is consistent with one side of the naphthyridine motif being doubly deprotonated and the other side being left intact. One N–*H* proton being still present in **1** could be additionally confirmed through the observation of a weak intensity vibration at 3502 cm^–1^ in the ATR-IR spectrum. Deprotonation of the free ligand in the related ^*t* **‑Bu**^ **PNNP** ligand can result in either the cis-isomer (i.e., the donor arm pointing toward the binding pocket) or the trans-isomer.^28^ To gauge which of the isomers was obtained in this case, a 2D-NOESY spectrum was recorded of **1**. The antiphase cross-peak between the methine resonance and one the naphthyridine resonances at δ = 6.02 ppm confirmed the formation of the cis-isomer (see ESI Figure S10). The same 2D-NOESY however also showed an in-phase cross-peak (EXSY) between the methine resonance and a very broad resonance at δ = 6.63 ppm. Recording VT ^1^H NMR spectra of a toluene-*d*_8_ solution of **1** at lower temperatures revealed sharpening of this resonance with a clear peak being visible at temperatures below 233 K (see ESI Figure S14). This allowed for integration, showing a 1:1 ratio with the methine resonance.

**Scheme 2:**
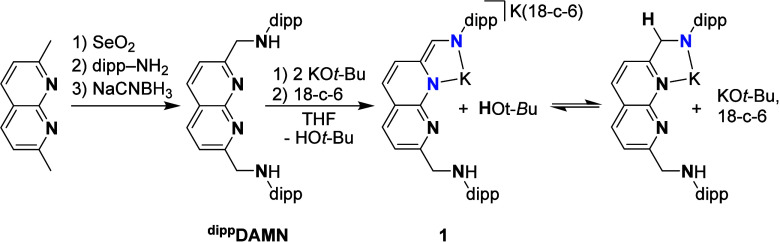
Synthesis of the Dinucleating ^**dipp**^ **DAMN** and Subsequent Deprotonation, Resulting in Complex **1** and the Observed Equilibrium with the Protonated Form

We attribute this broad resonance to the O–*H* proton of a *t*-BuOH molecule which reversibly protonates the methine carbon of **1**, resulting in the equilibrium depicted in Scheme 2. A closer inspection of the ^1^H NMR spectrum revealed a singlet at δ = 1.35 ppm, partially obfuscated by the dipp methyl resonances, which could potentially be attributed to the *t*-Bu protons of the exchanging *t*-BuOH. Extensive (freeze-)­drying of the compound did not lead to a decrease of the amount of exchanging *t*-BuOH, suggesting that in the solid state the equilibrium lies to the side of the nondearomatized ligand (Scheme 2, right). Interestingly, addition of an extra equiv of 18-c-6 did not result in the change of speciation, suggesting that one K^+^ ion is tightly bound in the naphthyridine pocket as depicted in Scheme 2.

Combined, these observations show that upon deprotonation of the secondary amine, the methylene linker on the same side becomes more acidic and gets subsequently deprotonated by the second equiv of KO*t*-Bu. This unexpected deprotonation of the ^**dipp**^ **DAMN** ligand led us to set our sights on a ligand design without methylene protons. By substituting the pendant secondary amines with pendant amides, we expected to retain the binding features ^**dipp**^ **DAMN** while suppressing the observed deprotonation of the methylene linkers.

### Ligand Synthesis and Deprotonation Studies of ^dipp^NDC

Consequently, the ^**dipp**^ **NDC** ligand (Scheme 3) was synthesized in 4 steps from 2,7-dimethyl-1,8-naphthyridine in an overall 44% yield (see ESI Section 1.5).^29^ ^**dipp**^ **NDC** was treated with two equiv of either KO*t*-Bu or KBn in C_6_H_6_ (Scheme 3, middle). Analysis of the reaction mixture (out of KBn) in C_6_H_6_ by ^1^H NMR spectroscopy (298 K, PRESAT) before removal of the solvent revealed clean conversion to a new species (**2**, see ESI Figure S28). The number of resonances observed are consistent with a species symmetric on each side of the mirror plane perpendicular to the naphthyridine plane. A singlet resonance attributed to the amide protons is absent, suggesting full deprotonation of the ligand. For the –C*H*_3_ protons on the dipp substituents, two doublets at δ = 1.32 and 1.20 ppm integrating to 12 H each were observed and for the methine protons only one resonance integrating to 4 H was observed. This difference in magnetic environment of the –C*H*_3_ groups can be indicative of slow or hindered rotation of the N–C_Ar_ bonds on the NMR time scale, rendering these the –C*H*_3_ groups diastereotopic. While removal of the solvent of **2** resulted in the irreversible formation of an intractable mixture of products, the addition of 2 equiv of 18-crown-6 enabled the isolation of **2**·2­(18-c-6) (Scheme 3). Inspection of the ^1^H NMR spectrum (in C_6_D_6_ at 298 K, see ESI Figure S29) shows a shift of the resonances compared to **2**, indicative of a change in the binding of K^+^. Additionally, the appearance of the – C*H*_3_ protons as a single (albeit broad) doublet at δ = 1.60 ppm which integrates to 24 H, suggests that the previously proposed constrained rotation of the N–C_Ar_ bonds might be alleviated upon sequestration of K^+^. Layering a saturated toluene solution of **2**·2­(18-c-6) with pentane resulted in the formation of single crystals, suitable for X-ray diffraction. Elucidation of the crystal structure (Figure 1) revealed the dimeric nature of **2**·2­(18-c-6) in the solid-state. Two independent crown ether-sequestered K^+^ ions are found per naphthyridine motif, residing above and below the naphthyridine plane, respectively. The ^**dipp**^ **NDC** ligands are bound to K^+^ *via* the imidate (*via* oxygen) binding mode rather than the amidate (*via* nitrogen) binding mode,^30^ with K–O_(lig)_ distances of 2.553(3) Å and 2.744(3) Å. There is a short contact between one of the sequestered potassium cations and a methyl group on one of the dipp groups of the other molecule in the dimer. This short K···C contact of 3.415(4) Å is within the range of reported examples of – CH_3_ to K^+^ agostic bonding where the K^+^ is sequestered by 18-crown-6.^31–34^

**Scheme 3:**
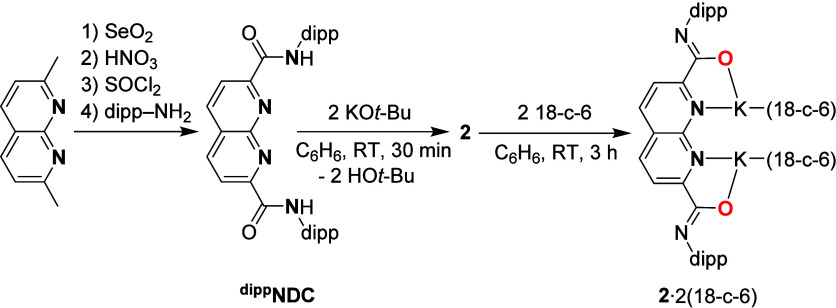
Syntheses of the ^**dipp**^ **NDC** Ligand, and Complexes **2** and **2**·2­(18-c-6)

**Figure 1:**
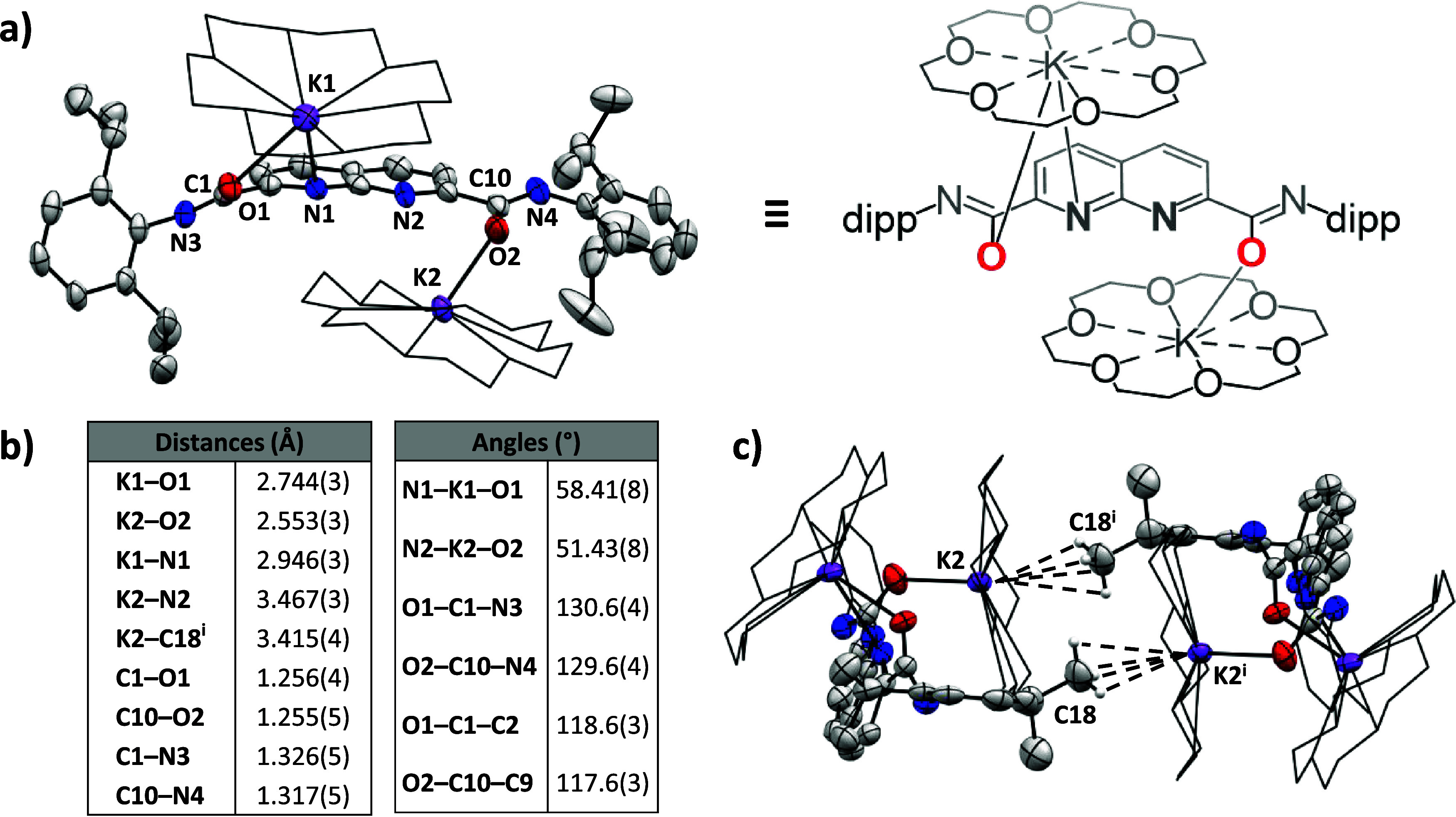
**a)** Displacement ellipsoid plot of **2**·2­(18-c-6) at 50% probability, flanked by a schematic representation of the perspective. Hydrogen atoms, minor disorder components and cocrystallized toluene are omitted for clarity. Additionally, only half of the dimer is depicted, and 18-crown-6 molecules are depicted as wireframe to enhance clarity. **b)** Selected bond distances and angles in Å and °, respectively. Symmetry code *i*: – x, 1–y, 1–z. **c)** Side-view of the dimer of **2**·2­(18-c-6), visualizing the agostic interactions between K^+^ and one of the –CH_3_ substituents on one of the dipp groups.

### ^dipp^NDCMg_2_Cl_2_

We hypothesized that transmetalation of **2**·2­(18-c-6) with metal precursors would most likely result in the retention of the imidate binding mode (*see below*). To investigate if we could access the amidate form of the ligand, we attempted direct metalation of the N–H bond using Grignard reagents, which contain an internal base. A reaction between ^**dipp**^ **NDC** and two equiv MeMgCl resulted in a bright orange solution concomitant with visible effervescence. From this mixture, **3** was isolated as a yellow solid in 74% yield (Scheme 4, left). Analysis of the ^1^H and ^13^C­{^1^H} NMR spectra (THF-*d*_8_, 298 K) revealed full conversion of the ligand to a new compound with a number of resonances consistent with a C_2v_-symmetry on the NMR time scale (see ESI Section 1.7). The observed symmetry and absence of N–*H* resonances are in line with full deprotonation of the ^**dipp**^ **NDC** ligand. Layering a saturated solution of **3** in THF with hexane and storing it at –40 °C for several days yielded orange single crystals suitable for analysis by X-ray diffraction.

**Scheme 4:**
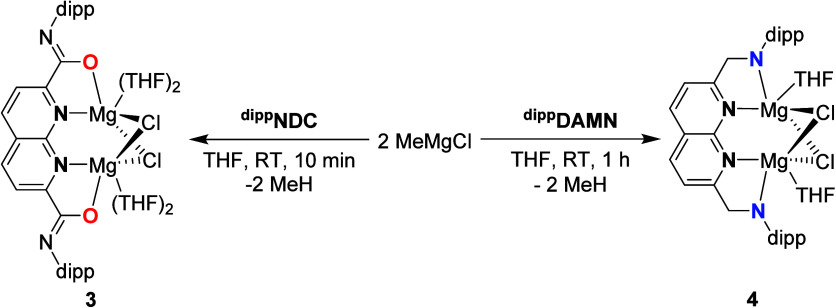
Syntheses of Complexes **3** and **4**

The solid-state structure of **3** (^**dipp**^ **NDC**Mg_2_Cl_2_·4THF, Figure 2b) revealed the formation of a dinuclear complex containing two independent Mg centers, one in each of the binding pockets of the ^**dipp**^ **NDC** ligand. Similar to **2**·2­(18-c-6), the imidate binding mode was obtained in the solid-state structure rather than the amidate binding mode, suggesting that the ligand readily tautomerizes upon deprotonation. Two THF molecules (cis to each other) are bound to each Mg center and together with the bridged chloride ligands a distorted octahedral geometry around Mg is obtained. The two Mg centers are positioned slightly outside of the naphthyridine plane (see ESI Figure S62), with the orientation of the axial ligands per center being antiparallel. The combination of this geometry with the variety of ligands on the Mg centers results in optical isomerism, with both the Λ,Λ and Δ,Δ compounds being formed in solution. The analyzed crystal was comprised of a single enantiomer of **3** (Δ,Δ). Having obtained structural insights and spectroscopic handles of **3**, we reacted **2**·2­(18-c-6) with 2 equiv of MgCl_2_ in THF. ^1^H NMR analysis of the reaction mixture showed **3** as a major product, thereby confirming our aforementioned hypothesis that this reaction would yield the imidate tautomer (see ESI Figure S36).

**Figure 2:**
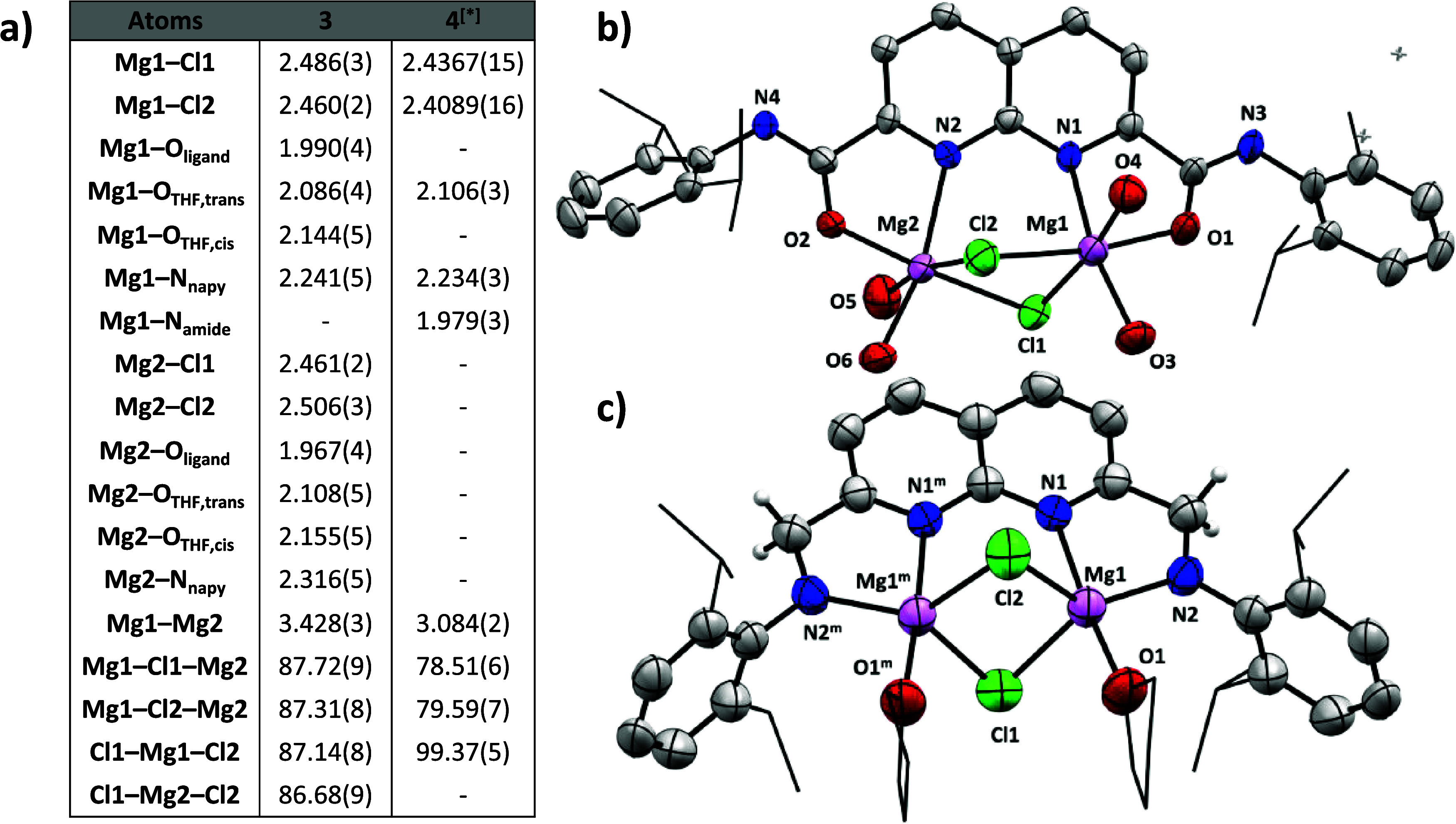
**a)** Selected bond distances and angles of compounds **3** and **4** in Å and °, respectively. ^[∗]^ **4** is located on a mirror plane. Only half of the distances and angles are independent. **b)** Displacement ellipsoid plot of the Δ,Δ enantiomer of **3** at 50% probability. Hydrogen atoms, the carbon backbones of coordinated THF and minor disorder components are omitted for clarity. The isopropyl groups on the dipp substituents are depicted as wireframe for clarity. **c)** Displacement ellipsoid plot of **4** at 50% probability. Most hydrogen atoms, severely disordered solvent molecules, and the minor disorder components of THF are omitted for clarity. The isopropyl groups on the dipp substituents are depicted as wireframe for clarity. Symmetry code m: x, 1/2–y, z.

### ^**dipp**^ **DAMN**Mg_2_Cl_2_·2 THF

The imidate binding mode found for **2** and **3** decreases the steric pressure around the dimagnesium core due to steric bulk of the dipp groups pointing away from the dinuclear core. In order to investigate the influence the decrease in steric bulk around the Mg_2_Cl_2_ core has on its coordination chemistry, we targeted the synthesis of the Mg_2_Cl_2_ complex of the ^**dipp**^ **DAMN** ligand for direct comparison with **3**. Addition of ^**dipp**^ **DAMN** to a THF solution of 2 equiv of MeMgCl led to clean formation of **4** as a bright orange solid in quantitative yield (Scheme 4, right). Analysis of the ^1^H NMR and ^13^C­{^1^H} NMR spectra (C_6_D_6_, 298 K) showed a number of resonances consistent with retention of the C_2v_-symmetry of the ligand (see ESI Section 1.9). The loss of the N–*H* resonances, the presence of only two naphthyridine resonances (δ = 7.00 and 6.61 ppm) and the appearance of a singlet at δ = 5.08 ppm integrating to 4H, which we attribute to the methylene linkers, all suggested that the secondary amines are selectively deprotonated by the methyl groups of the Grignard reagent, contrasting the deprotonation order observed for **1**. In addition, broad resonances are observed at δ = 3.65 and 1.25 ppm suggesting that THF is bound to the Mg centers in solution.

Layering a saturated THF solution of **4** with pentane at –40 °C led to the formation of orange needles suitable for analysis by X-ray diffraction. The solid-state structure of **4** (Figure 2c) agrees with the spectroscopic data and confirms the selective deprotonation of the N–H protons by the Grignard reagent, with each Mg center residing in a bidentate binding pocket of the dinucleating ligand. In the crystal, **4** is located on a mirror plane. The two chlorides bridge the two Mg centers in a “diamond core” fashion and a THF ligand per Mg completes the observed trigonal bipyramidal geometry (τ = 0.75), with the naphthyridine N-donor and the THF O-donor occupying the axial positions. The bond between the naphthyridine N-donor and Mg (Mg1–N1 = 2.234(3) Å) is substantially longer than the bond between Mg and the pendant amide (Mg1–N2 = 1.979(3) Å), consistent with the neutral and anionic character of these donors, respectively.^35^

### Structural Insights

With the solid-state structures of the **L**Mg_2_Cl_2_ complexes determined, a comparison of the structural features and the bond metrics is possible. The most striking difference between the two complexes is the geometry around the Mg centers (Figure 2), being octahedral in **3** and trigonal bipyramidal in **4**. We ascribe this change in geometry to the imidate binding mode of the ^**dipp**^ **NDC** ligand in **3**, which results in a decrease of steric bulk around the Mg centers (for a quantification of the steric bulk in both complexes see below). The decreased steric encumbrance allows for the binding of an additional THF molecule and implies that the Mg centers in **4** are coordinatively unsaturated. The increased steric and electronic saturation in **3** results in longer Mg–Cl bonds compared to the same bonds found for **4** (2.460(2)-2.506(3) Å in **3** vs 2.4089(16)-2.4367(15) Å in **4**). The binding of an additional THF molecule in **3** also has a significant influence on the Mg···Mg distance. This distance in **3** is significantly larger than the distance in **4** (3.428(3) Å vs. 3.084(2) Å respectively). This is due to one of the Mg centers in **3** being pushed outside of the naphthyridine plane and one slightly below the naphthyridine plane (as is evident from their respective torsion angles χ­[O1,C1,C2,N1]= – 10.6(8)° and χ­[O2,C10,C9,N2]= – 2.7(8)°, see ESI Figure S62) to minimize the distortion of the octahedral geometry. The distance of Mg1 to the least-squares plane of the naphthyridine ring is – 0.897(2) Å and the corresponding distance for Mg2 is 0.495(2) Å. Contrastingly, the Mg centers in **4** reside in the naphthyridine plane, with the distance of Mg1 to the least-squares plane of the naphthyridine ring being 0.0799(10) Å. To quantify the steric environments surrounding the Mg_2_ cores in **3** and **4** imposed by each of the ligands, we used the SambVca 2.1 web application to build their respective steric maps.^36^ For dinuclear systems, choosing the centroid and radius of the defined sphere for the buried volume calculations is nontrivial, and based on previous work from our group we placed the centroid of the sphere in between the metal centers and set the radius of the defined sphere to 5 Å.^37^ The steric maps in Figure 3 reveal that the largest difference in steric encumbrance in the reaction hemisphere of the two complexes is in the *cis* ligand void space (i.e., above and below the naphthyridine plane).^38^ In the case of **4**, the *i*-Pr moieties on the dipp substituents shield the Mg centers above and below the naphthyridine plane, preventing the coordination of additional THF molecules. In the case of **3**, the dipp substituents are oriented away from the Mg centers, which significantly decreases the shielding of the *cis* void spaces by the *i*-Pr groups. The difference in the steric properties of the ligands in **3** and **4** are also expressed in their respective buried volumes (*V*_bur_) of 27.3% and 35.4%. We envision that these pronounced differences in steric profiles between the two complexes can lead to diverging reactivity due to the varying accessibility of the Mg centers for substrates.

**Figure 3:**
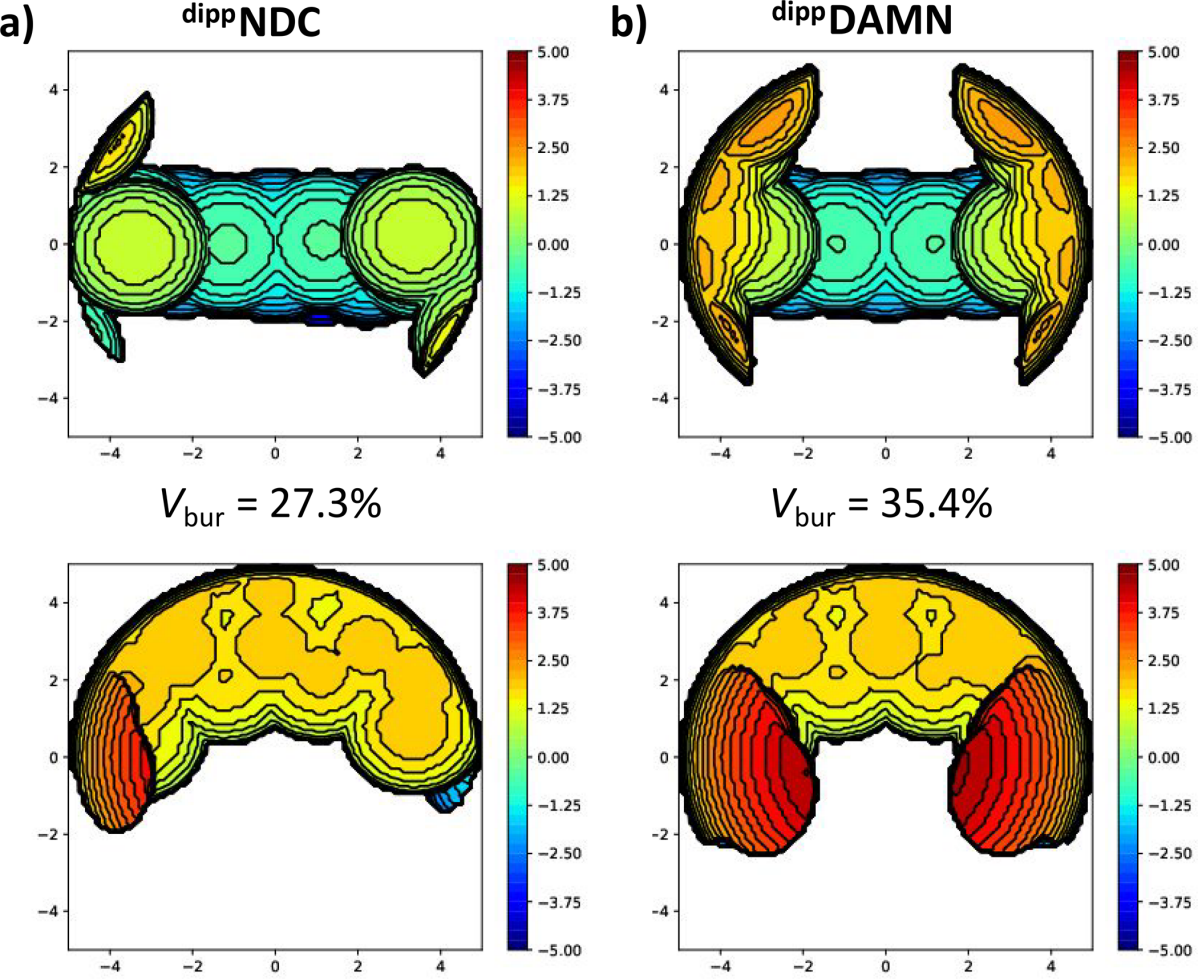
Steric maps and associated buried volumes of **a)** the ^**dipp**^ **NDC** ligand as bound to **3** and **b)** the ^**dipp**^ **DAMN** ligand as bound to **4**. The top maps show the front view of the ligand systems (parallel with the naphthyridine plane), and the bottom maps show the top-down view of the ligand systems (perpendicular to the naphthyridine plane).

### ^**dipp**^ **DAMN**Mg_2_(*n*-Bu)_2_·2 THF

The discrepancy in deprotonation order observed for **1** and **4**, along with the relative acidity of the methylene protons, prompted us to investigate whether treating ^**dipp**^ **DAMN** with two equiv of a dialkyl-magnesium species would result in deprotonation and subsequent dearomatization of the ligand, similar to what is observed for the related ^*t* **‑Bu**^ **PNNP** ligand when treated with strong bases.^39,40^ Treating a THF solution of ^**dipp**^ **DAMN** with 2 equiv of Mg­(*n*-Bu)_2_ (Scheme 5) resulted in clean formation of **5** as a brown solid in quantitative yield. The number of resonances in the ^1^H- and ^13^C­{^1^H}-NMR spectra (see ESI Section 1.10) is consistent with a C_2v_-symmetric compound. Broad resonances at δ = 3.37 and 1.18 ppm (C_6_D_6_, 298 K), integrating to 8H, are indicative of **5** binding two THF molecules in solution. Additional resonances at δ = 1.49 (overlapping with the –CH­(C*H*_3_)_2_ resonance of the dipp group, 4H), 1.31 (4H), 0.90 (6H) and the characteristic AA’BB’ resonance at 0.01 ppm (4H) are consistent with two butyl groups being present in the complex.

**Scheme 5:**
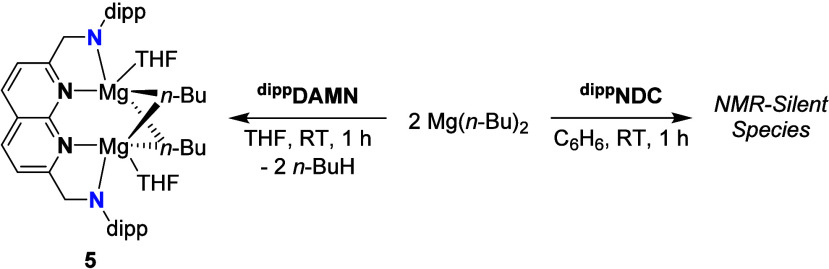
Reactions of Mg­(*n*-Bu)_2_ with the ^**dipp**^ **DAMN** and ^**dipp**^ **NDC** Ligands, Resulting in the Formation of **5** and a NMR-Silent Species, Respectively

Storing a saturated THF/pentane solution of **5** at – 40 °C yielded dark needle-shaped crystals suitable for X-ray diffraction (Figure 4). The solid-state structure revealed a distorted trigonal bipyramidal geometry around the Mg centers, with each butyl group bridging the Mg centers. The apical positions of the bipyramids are occupied by the N-donor of the naphthyridine ring and by a O-donor from THF. The 1:1 ratio between coordinated THF and Mg in the solid-state structure is consistent with earlier observations in solution. The N3–Mg1–C35 and N3–Mg1–C39 angles (125.10(15)° and 129.42(15)° respectively) are enlarged, while the C35–Mg1–C39 angle (104.92­(15)°) is contracted compared to ideal trigonal bipyramidal geometry, with similar trends observed for the second Mg center (τ = 0.65 and 0.71, respectively). The Mg1–Mg2 distance (2.7499(18) Å) and Mg–C bond lengths (2.302(4)-2.321(4) Å) align with metrics reported for crystallographically characterized Mg_2_–alkyl_2_ species with a similar “diamond-core”.^14,18,24,40–45^ The observation of the preferential formation of this core over deprotonation of the relatively acidic methylene protons is most likely attributed to the bridging binding mode of the butyl groups, which delocalizes their negative charge and renders them less basic. The Mg–C bond lengths are slightly longer than in most of these structures and likely reflect the THF coordination and higher electronic saturation of the Mg centers. The bond lengths of Mg1–N3 and Mg2–N4 (2.022(3) and 2.020(4) Å respectively) are considerably shorter than the Mg1–N1 and Mg2–N2 bonds (2.247(3) and 2.220(3) Å respectively), which is in line with their higher anionic character.

**Figure 4:**
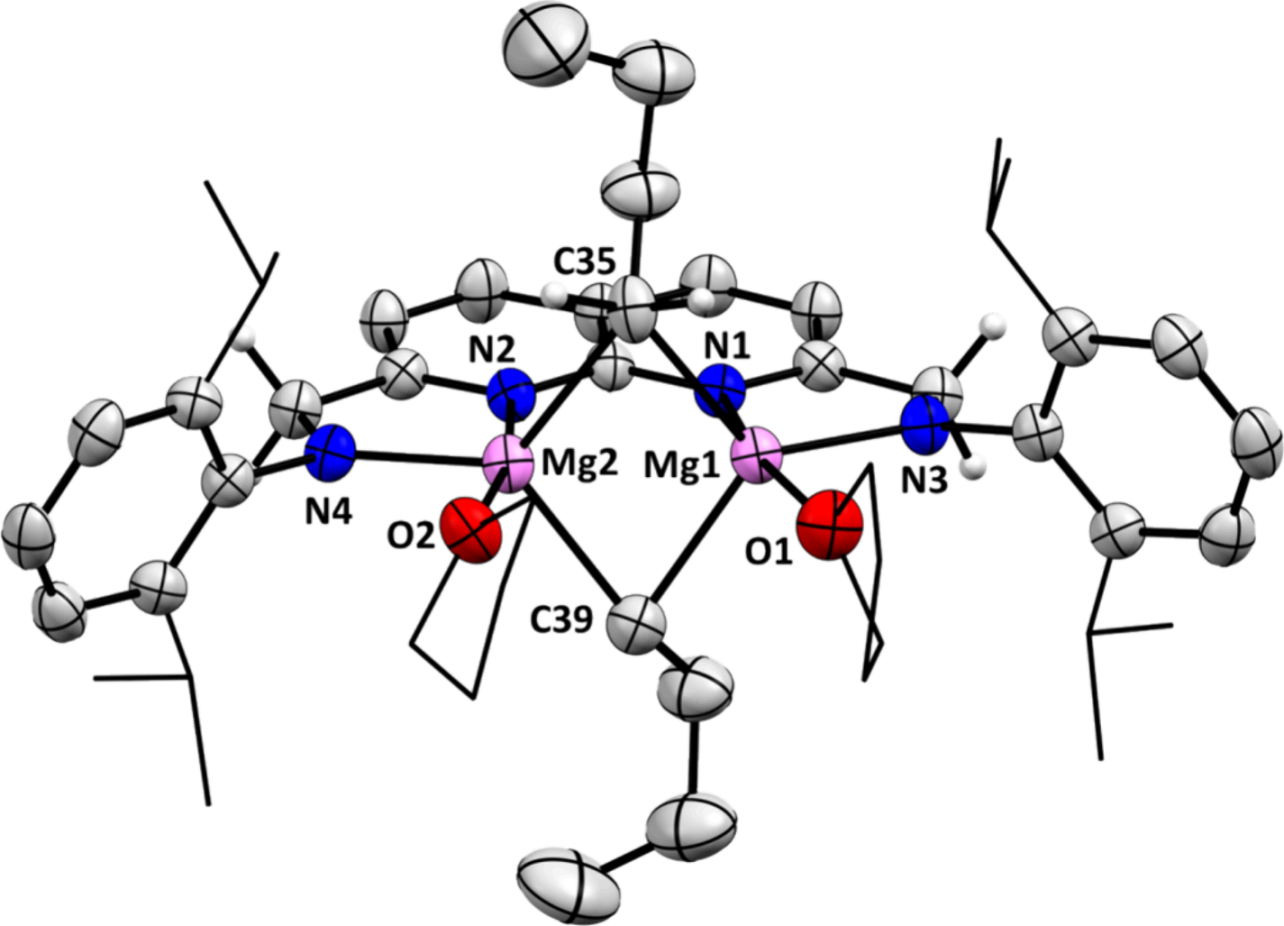
Displacement ellipsoid plot of **5** at 50% probability. Most hydrogen atoms, cocrystallized solvent molecules and minor disorder components are omitted and parts of the THF molecules and the dipp substituents on N are depicted as wireframe for clarity. The hydrogen atoms on C35 were found in the difference Fourier maps.

### ^**dipp**^ **NDC**Mg_2_(*n*-Bu)_2_

We explored if the dibutyl-magnesium chemistry observed for the ^**dipp**^ **DAMN** ligand also translated to the ^**dipp**^ **NDC** ligand. Reacting a benzene solution of ^**dipp**^ **NDC** with 2 equiv of Mg­(*n*-Bu)_2_ resulted in effervescence and a dark green solution. Interestingly, ^1^H NMR (PRESAT) analysis of the reaction mixture revealed the formation of an NMR-silent species (see ESI Figure S49), contrasting the described reactivity with ^**dipp**^ **DAMN**. X-band EPR spectroscopic analysis of the reaction mixture revealed an intense isotropic signal (*g*_iso_ = 2.0036) without well-resolved hyperfine interactions, consistent with the presence of paramagnetic species (Figure S50–S52). Despite repeated attempts, we were unable to grow single crystals suitable for analysis by X-ray diffraction out of the reaction mixture. To better understand the nature of the complexes formed, we quenched the reaction mixture with water (see ESI Section 1.11). ^1^H NMR analysis (C_6_D_6_, 298 K) after the aqueous workup revealed multiple resonances, consistent with the formation of several compounds. Of particular interest were the resonances between δ = 5.91 and 4.85 ppm, consistent with protons on dearomatized pyridine rings.^41,42^ The ^1^H–^1^H COSY NMR spectrum (see ESI Figure S55) showed coupling between the resonance at δ = 4.85 ppm and a doublet at δ = 3.23 ppm, obfuscated by the -dipp – C*H* septets. These shifts are similar to those reported for the 3- and 4-positions of a para-coupled bis-naphthyridine system (δ = 5.0 and 3.4 ppm respectively, in toluene-*d*_8_).^43^ Such para-coupling arises from the radical recombination of naphthyridine-centered radicals, as observed for dinickel complexes (Uyeda group) and dicopper complexes (Tilley group).^43,44^ Homonuclear and heteronuclear 2D NMR spectra (see ESI Figures S57–58) reveal that these resonances attributed to a para-coupled bis-naphthyridine arise from a distinct molecule. The remaining resonances in the δ = 5.91–4.85 ppm region correspond to a molecule bearing a butyl group, as evidenced by characteristic resonances at δ = 1.96 (α–C*H*_2_) and 0.82 ppm (−C*H*_3_). ESI-MS analysis (positive mode, see ESI Figure S59) of the reaction mixture postaqueous workup revealed signals at *m*/*z* = 592.5, 617.2 and 1195.8, which can be attributed to the [M]^+^ (M = ^**dipp**^ **NDC** ^**Bu**^), [M + Na]^+^ (M = ^**dipp**^ **NDC** ^**Bu**^ **H**), and [M+Na+MeCN]^+^ (M = ^**dipp**^ **NDC** ^**Bu**^ **H–** ^**dipp**^ **NDCH**), respectively (Scheme 6).

**Scheme 6:**
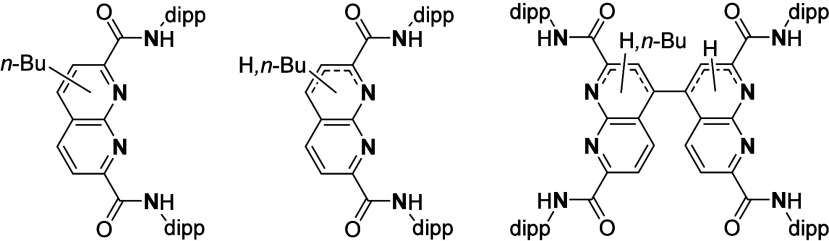
Some of the Identified Products That Form upon Quenching the NMR-Silent Reaction Mixture

Combining these data, we propose that a ^**dipp**^ **NDC**Mg_2_(*n*-Bu)_2_ species initially forms, with the observed effervescence most likely arising from butane liberation. Consistent with previous observations, we expect that the ligand will be in the imidate form. This complex most likely undergoes Mg–C homolysis, similar to what is observed for dialkylmagnesium complexes bound to conjugated diimines, bis-pyridines, and di-iminopyridines.^45^ DFT calculations on truncated **L**Mg_2_Me_2_ models (see ESI Sections 2.4–2.7) reveal a Gibbs free energy difference of 38.3 kcal·mol^–1^ between the singlet and triplet states of the ^**dipp**^ **DAMN**-based complex. In contrast, the same difference for the ^**dipp**^ **NDC**-based complex is only 20.8 kcal·mol^–1^, making it thermally accessible at room temperature. Notably, the geometry optimization of the triplet state of ^**dipp**^ **NDC**Mg_2_Me_2_·4 THF consistently resulted in extrusion of a methyl radical (see ESI Figure S61), highlighting the energetically facile nature of Mg–C homolysis in this case. For reported mononuclear complexes with pyridine-based ligands, homolysis of M–C bonds (M = Li, Mg, Al, Zn) often leads to alkylation of various positions of the pyridine, including the pyridinic nitrogen.^41,46,47^ This process is proposed to occur via single-electron transfer from the Mg–C bond to the pyridine backbone, followed by radical recombination with the liberated alkyl radical (Scheme 7, left).^48,49^ If the alkyl radical escapes the solvent cage, the resulting ligand-centered radicals can reversibly dimerize (Scheme 7, right).^50,51^ In these literature examples, both alkylation and dimerization products are diamagnetic, which contrasts with our observations for the ^**dipp**^ **NDC** ligand. We propose that the observed paramagnetism most likely results from the extended conjugated system of the ^**dipp**^ **NDC** ligand, which can stabilize ligand-centered radicals better than the mononuclear analogues, and pushes the alkylation and dimerization equilibria toward the ligand-centered radical side (Scheme 7, for a discussion of the proposed equilibria, see ESI Section 1.12).^42,50,51^ While the exact nature of the species formed in this reaction remains elusive, the combined NMR, EPR and ESI-MS data suggest a complex mixture of products, including butylated and coupled derivatives of the ^**dipp**^ **NDC** ligand. These products arise from the generated butyl radical being either trapped by or escaping the solvent cage, respectively.

**Scheme 7:**
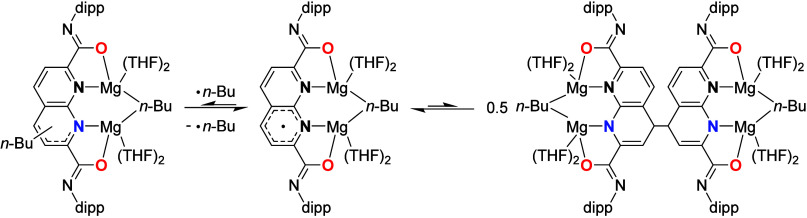
Proposed Equilibria That Potentially Can Take Place upon Mg–C Homolysis[Fn sch7-fn1]

a
*n*-Bu radical for the middle structure not depicted for clarity. Left proposed equilibrium (butylation) can result upon trapping of the *n*-Bu radical by the solvent cage, alternatively, the right proposed equilibrium (dimerization) can result upon the *n*-Bu radical escaping the solvent cage.

## Conclusions

In conclusion, this work highlights the significant influence ligand architecture can have on the coordination chemistry of dimagnesium complexes supported by dinucleating naphthyridine-based ligands. For the described reactivity of the ^**dipp**^ **NDC** and ^**dipp**^ **DAMN** ligands with Grignard reagents this influence is of steric nature, with the steric bulk surrounding the Mg_2_Cl_2_ core differing markedly between the two ligands. This divergence is expressed in different coordination geometries around the Mg centers, which largely depend on the degree of coordinative saturation that is allowed by the steric bulk imposed by the dinucleating ligands. In contrast, the described reactivity of the ^**dipp**^ **NDC** and ^**dipp**^ **DAMN** ligands with dialkylmagnesium reagents highlights how changes in the ligand backbone can have a pronounced influence on the stability of the resulting Mg_2_Bu_2_ complexes. Whereas ^**dipp**^ **DAMN** forms a well-defined, diamagnetic Mg_2_(*n*-Bu)_2_ complex, the extended conjugated system of ^**dipp**^ **NDC** results in paramagnetic species that form upon Mg–C homolysis and subsequent radical reactivity. Collectively, these findings establish a framework for leveraging ligand design to tailor the reactivity of organomagnesium complexes, opening avenues for the development of novel organometallic transformations.

## Supplementary Material





## Data Availability

An initial draft of this manuscript was uploaded to ChemRxiv.^52^ The data supporting this article have been included as part of the ESI, the raw data can be accessed through the Yoda repository, DOI: 10.24416/UU01-3IJEWU. CCDC 2422290 (^**dipp**^ **NDC**K_2_·2­(18-c-6)), 24222291 (^**dipp**^ **NDC**Mg_2_Cl_2_·4 THF), 2422292 (^**dipp**^ **DAMN**Mg_2_Cl_2_·2 THF) and 2371205 (^**dipp**^ **DAMN**Mg_2_(*n*-Bu)_2_·2 THF) contain the supplementary crystallographic data for this paper. These data can be obtained free of charge from The Cambridge Crystallographic Data Centre via www.ccdc.cam.ac.uk/data_request/cif.

## Notes

Synthesis and characterization of compounds, spectra, and crystallographic and computational details (PDF)
XYZ coordinates for the optimized structures (XYZ)
