# Supplementary material for: Divergent Organomagnesium Reactivity of Rigid, Dinucleating Naphthyridine Ligands: Backbone Changes with Big Impact
Source: Organometallics. 2025 May 16;44(10):1057–66. doi: 10.1021/acs.organomet.5c00070 (PMC12117560; doi:10.1021/acs.organomet.5c00070)
Supplement: Supplementary file 1 [file om5c00070_si_001.pdf]

SUPPORTING INFORMATION FOR

Divergent Organomagnesium Reactivity of Rigid, Dinucleating Naphthyridine Ligands: Backbone Changes with Big Impact

Errikos Kounalis,[a] Marieke M. Broekman,[a] Puck Uyttewaal,[a] Uladzislava Dabranskaya,[a] Martin Lutz,[b] Daniël L. J. Broere*[a]

[a] Organic Chemistry & Catalysis, Institute for Sustainable and Circular Chemistry, Faculty of Science, Utrecht University, Universiteitsweg 99, 3584 CG, Utrecht, The Netherlands

[b] Structural Biochemistry, Bijvoet Centre for Biomolecular Research, Faculty of Science, Utrecht University, Universiteitsweg 99, 3584 CG, Utrecht, The Netherlands

***Corresponding Author**

d.l.j.broere@uu.nl

Contents

1. Experimental methods: ... S3
1.1 General Considerations: ... S3
1.2 Synthesis of ^{dipp}DAMN: ... S4
1.3 Deprotonation of ^{dipp}DAMN with 2 equiv KO*t*-Bu: ... S8
1.4 Synthesis of $^{dipp}DAMNK_2$(18-c-6)·HO*t*-Bu: ... S9
1.5 Synthesis of ^{dipp}NDC: ... S14
1.6 Synthesis of $^{dipp}NDCK_2$·2(18-c-6): ... S18
1.7 Synthesis of $^{dipp}NDCMg_2Cl_2$·4 THF: ... S23
1.8 Alternate Synthesis of $^{dipp}NDCMg_2Cl_2$·4 THF: ... S27
1.9 Synthesis of $^{dipp}DAMNMg_2Cl_2$·2 THF: ... S28
1.10 Synthesis of $^{dipp}DAMNMg_2(n\text{-Bu})_2$·2 THF: ... S32
1.11 Reacting ^{dipp}NDC with $Mg(n\text{-Bu})_2$: ... S36
1.12 Discussion of the Proposed Equilibria: ... S41
2. Computational Methods: ... S43
2.1 General Considerations: ... S43
2.2 Example Input File for Geometry Optimisations: ... S43
2.3 Example Input File for SP Calculations: ... S43
2.4 Calculated Energies of $^{dipp}DAMNMg_2Me_2$·2 THF (Singlet): ... S43
2.5 Calculated Energies of $^{dipp}DAMNMg_2Me_2$·2 THF (Triplet): ... S44
2.6 Calculated Energies of $^{dipp}NDCMg_2Me_2$·4 THF (Singlet): ... S44
2.7 Calculated Energies of $^{dipp}NDCMg_2Me_2$·4 THF (Triplet): ... S44
3. X-ray crystal structure determination: ... S45
3.1 Crystal structure determination of $^{dipp}NDCK_2$·2(18-c-6): ... S45
3.2 Crystal structure determination of $^{dipp}NDCMg_2Cl_2$·4 THF: ... S46
3.3 Crystal structure determination of $^{dipp}DAMNMg_2Cl_2$·2 THF: ... S47
3.4 Crystal structure determination of $^{dipp}DAMNMg_2(n\text{-Bu})_2$·2 THF: ... S47
4. References ... S48

1. Experimental methods:

1.1 General Considerations:

All manipulations were performed under inert atmosphere using standard Schlenk techniques or inside a N_2-filled MBraun UNILABplus or N_2-filled MBraun MB200B glovebox using anhydrous solvents and reagents, unless stated otherwise. Glassware was dried under vacuum at 130 °C before use. Solvents were collected from a MBraun MB-SPS-800 solvent purification system and stored over 4 Å molecular sieves, except for CH_2Cl_2, which was stored over 3 Å molecular sieves. CH_3CN was dried over 3 Å molecular sieves and subsequently filtered over activated alumina. THF was distilled from a purple ketyl solution and degassed by sparging with N_2. All solvents were degassed by sparging with N_2. Deuterated solvents were obtained from Cambridge Isotope Laboratories, degassed by 3 freeze-pump-thaw cycles followed by backfilling with N_2 and stored over molecular sieves. All commercial reagents were obtained from Sigma Aldrich, Strem, Fischer Scientific or Acros and used without further purification. NMR data was recorded on an Agilent MRF400 equipped with an oneNMR probe and Optima Tune system, on a Varian VNMR-S-400 equipped with an AutoX probe and Agilent ProTune probe tuning accessory or on a 400 MHz Jeol EZCL G system with an HFX probe. Spectra were recorded at 298 K and chemical shifts (δ) are given in ppm referenced to (one of) the residual solvent peak (7.16 for C_6D_6, 3.58 for THF-d_8, 2.09 for toluene-d_8). All resonances in ^{13}C-NMR were referenced to the solvent. IR spectra were recorded on a FT-IR PerkinElmer Spectrum Two™ spectrophotometer equipped with an ATR-probe. ESI-MS measurements were performed on a Advion Expression LCMS equipped with and automated Plate Express TLC plate reader. Elemental analyses were performed by MEDAC ltd. in the United Kingdom. EPR spectra were recorded on a Bruker EMXPlus X-band spectrometer. Column chromatography was performed using Merck silica gel (60–200 mesh). Organomagnesium reagents were titrated regularly to ensure correct concentrations. Diisopropylphenyl naphthyridine bisaldimine (^{dipp}**NBA**) and 1,8-naphthyridine-2,7-dicarboxaldehyde were synthesised according to literature procedures.[1,2]

1.2 Synthesis of ^{dipp}DAMN:

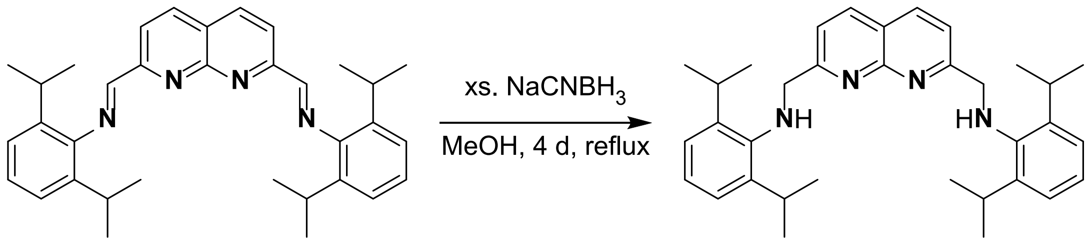

In a N_2-filled Schlenk flask, ^{dipp}**NBA** (1.13 g, 2.24 mmol, 1.0 equiv) was dissolved in dry and degassed MeOH (130 mL). $NaCNBH_3$ (5.90 g, 93.9 mmol, 41.9 equiv) was added all at once to the solution at 0 °C. The mixture was stirred for 15 min at 0 °C, warmed to RT and then refluxed for 4 d. The mixture changed from a yellow to wine-red suspension to a clear orange solution. The progress of the reaction was monitored by TLC. The mixture was allowed to cool to RT and the solvent was evaporated *in vacuo*. A degassed K_2CO_3(aq) (4 M, 160 mL) solution was added to the solids and the mixture was stirred thoroughly for 15 min. The mixture was extracted with degassed CH_2Cl_2 (3 x 80 mL). The organic fractions were collected in a Schlenk flask via cannula transfer and dried over Na_2SO_4. The mixture was separated by cannula filtration and the solvent of the filtrate evaporated under vacuum. The resulting solids were transferred to a glovebox and extracted with pentane (350 mL). The solvent was removed *in vacuo* to yield ^{dipp}**DAMN** as a tan solid (995 mg, 1.96 mmol, 88% yield). Crystalline material can be obtained by storing a saturated pentane solution at -40°C.

Note: Aerobic solutions of ^{dipp}**DAMN** slowly oxidise to the starting imine, a NMR-sample (20 mg, approx. 65 mM) that has been exposed to air shows almost complete conversion within 16 h. Similar oxidations are also observed upon attempted purification by silica gel-based column chromatography.

^{1}H NMR (400 MHz, C_6D_6, 298 K): δ 7.32 (d, $^3J_{H,H}$ = 8.2 Hz, 2H), 7.20-7.12 (m, 6H*), 6.83 (d, $^3J_{H,H}$ = 8.2 Hz, 2H), 5.09 (t, $^3J_{H,H}$ = 5.9 Hz, 2H), 4.41 (d, $^3J_{H,H}$ = 5.9 Hz, 4H), 3.68 (sept, $^3J_{H,H}$ = 6.9 Hz, 4H), 1.30 (d, $^3J_{H,H}$ = 6.9 Hz, 24H). *Overlapping signals

^{13}C{^{1}H}-NMR (APT, 101 MHz, C_6D_6, 298 K): δ 163.1, 155.8, 144.4, 143.0, 136.7, 124.4, 124.0, 120.8, 120.7, 57.4, 28.3, 24.1.

ATR-IR (cm^{-1}): 3351 (m), 2960 (s), 2924 (m), 2867 (m), 1611 (m), 1459 (m), 1362 (w), 1256 (w), 855 (w), 806 (w), 753 (m).

Elemental Analysis: Calcd. For $C_{34}H_{44}N_4$: C, 80.27; H, 8.72; N, 11.01. Found C, 80.11; H, 9.03; N, 10.93.

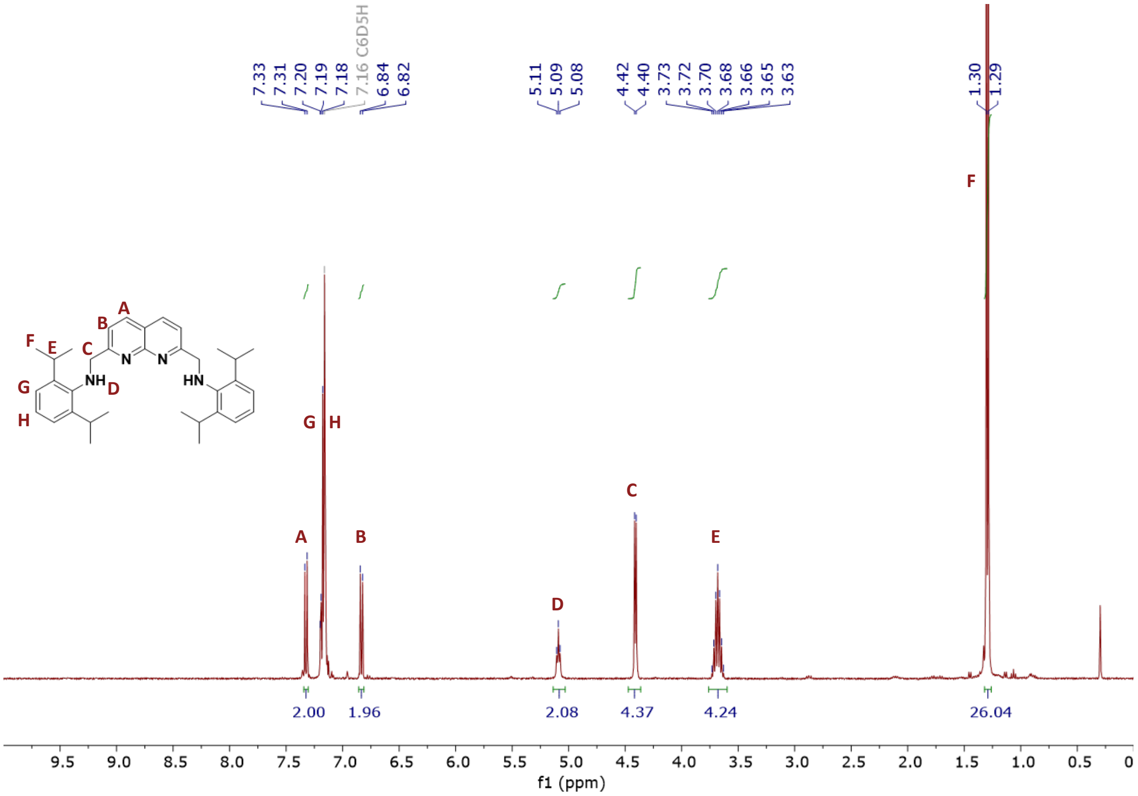

Figure S1: ^{1}H-NMR spectrum of $^{\mathbf{dipp}}$**DAMN** in C_6D_6 at 25 °C.

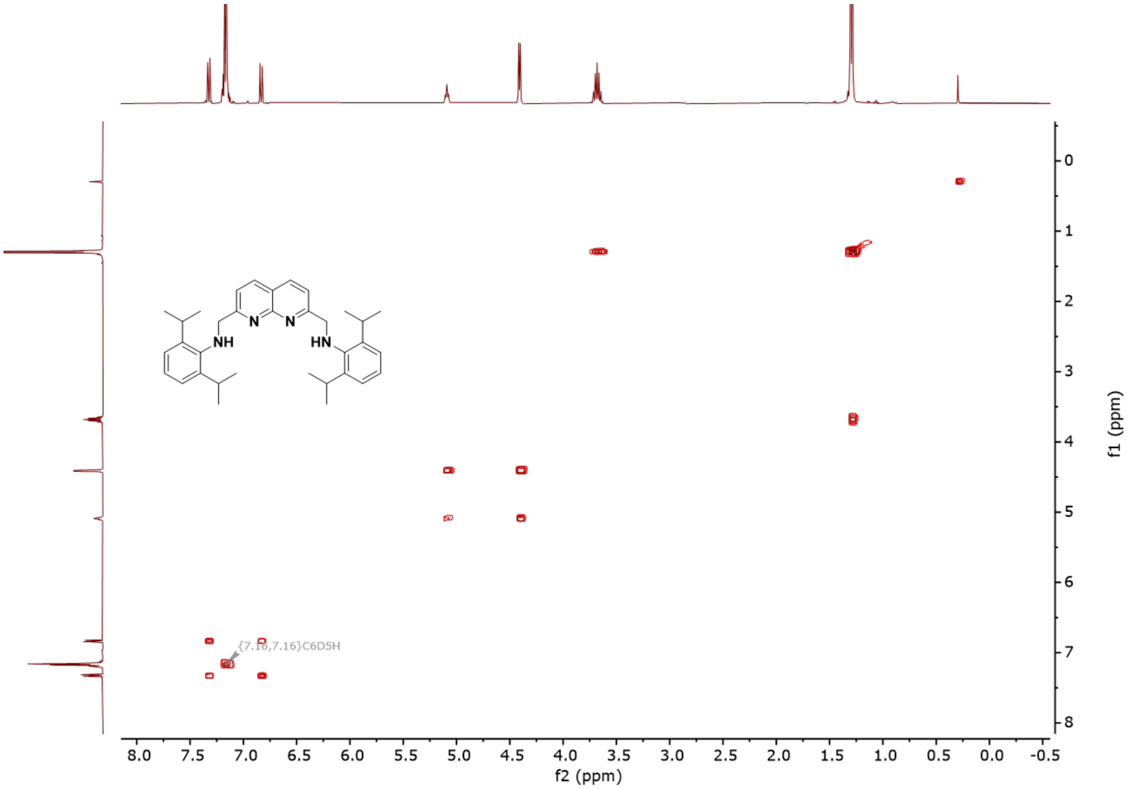

Figure S2: ^{1}H gCOSY spectrum of $^{\mathbf{dipp}}$**DAMN** in C_6D_6 at 25 °C.

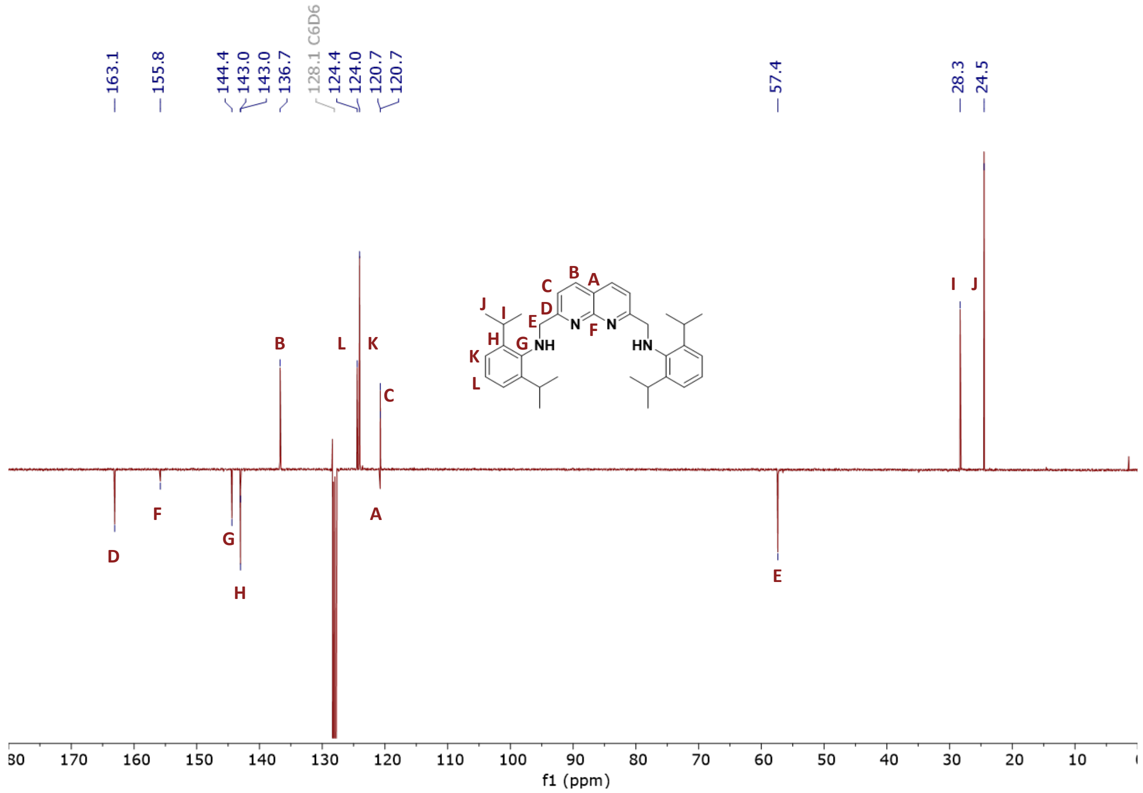

Figure S3: $^{13}C\{^{1}H\}$-NMR (APT) spectrum of **^{dipp}DAMN** in C_6D_6 at 25 °C.

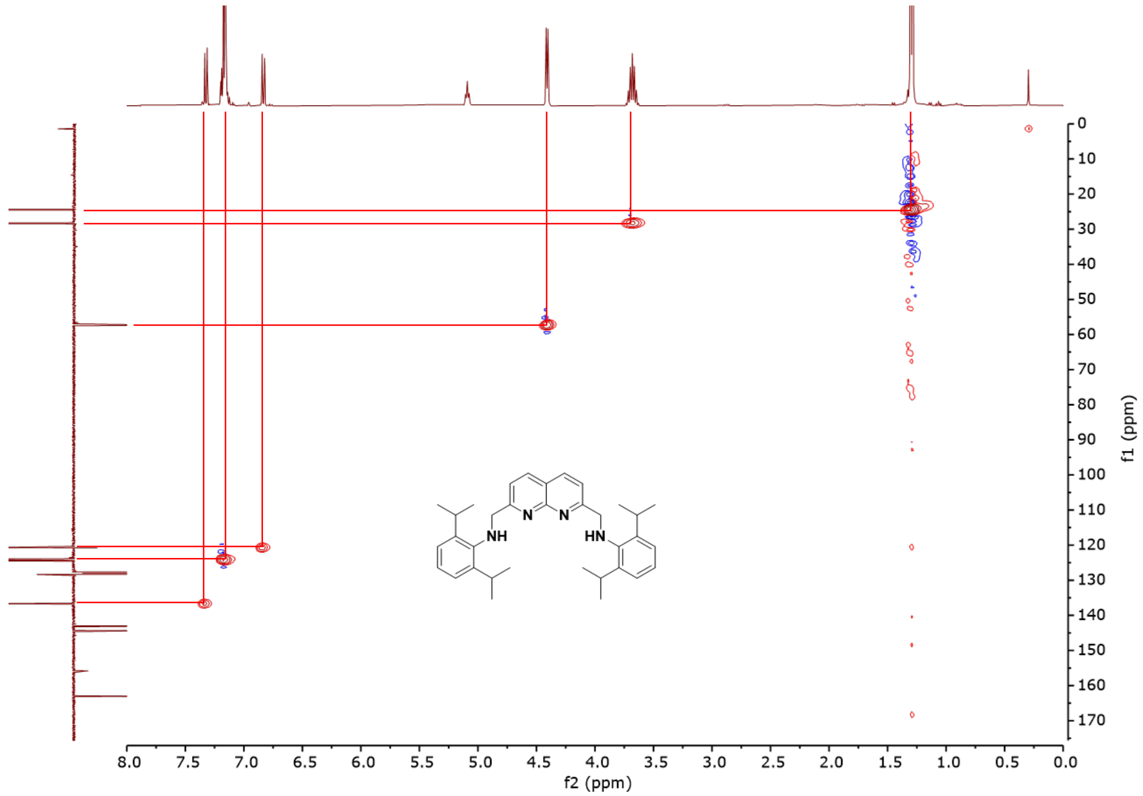

Figure S4: ^{1}H-^{13}C ASAP-HMQC NMR spectrum of **^{dipp}DAMN** in C_6D_6 at 25 °C.

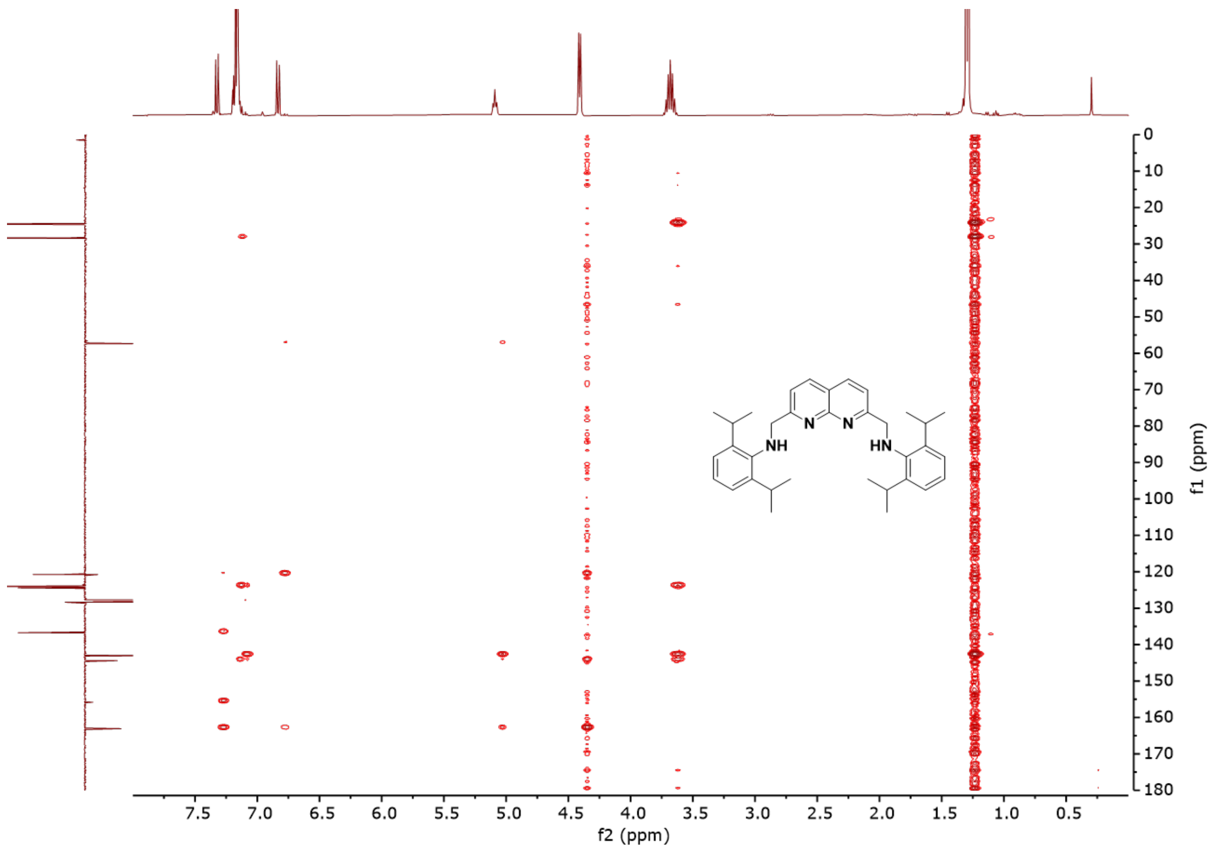

Figure S5: ^{1}H-^{13}C HMBC NMR spectrum of **^{dipp}DAMN** in C_6D_6 at 25 °C.

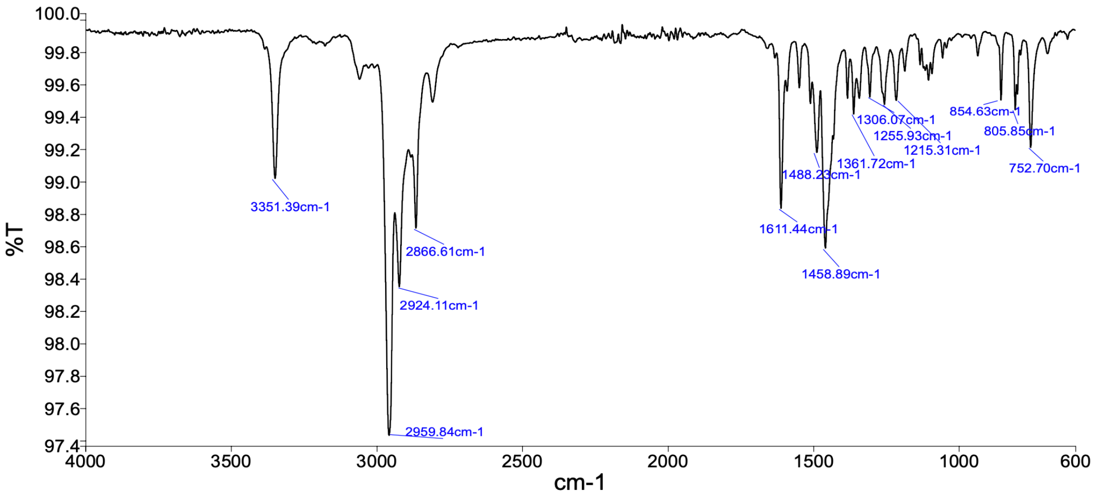

Figure S6: ATR-IR spectrum of **^{dipp}DAMN** measured as a film under N_2 flow at 25 °C.

1.3 Deprotonation of ^{dipp}DAMN with 2 equiv KO*t*-Bu:

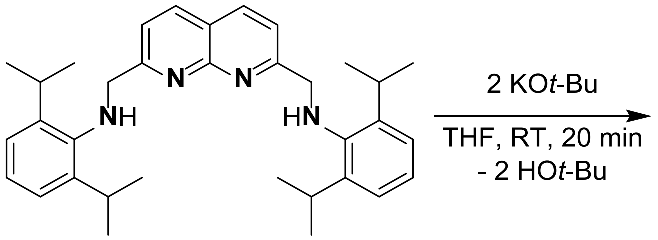

A solution of KO*t*-Bu (4.4 mg, 39.4 µmol, 2.0 equiv) in THF (1.5 mL) was added dropwise to a stirring solution of **^{dipp}DAMN** (10.0 mg, 19.7 µmol, 1.0 equiv) in THF (1.5 mL), turning the solution dark orange. The mixture was left to stir at ambient temperature for 20 min after which the solvent was removed in vacuo yielding 18.1 mg of dark orange film.

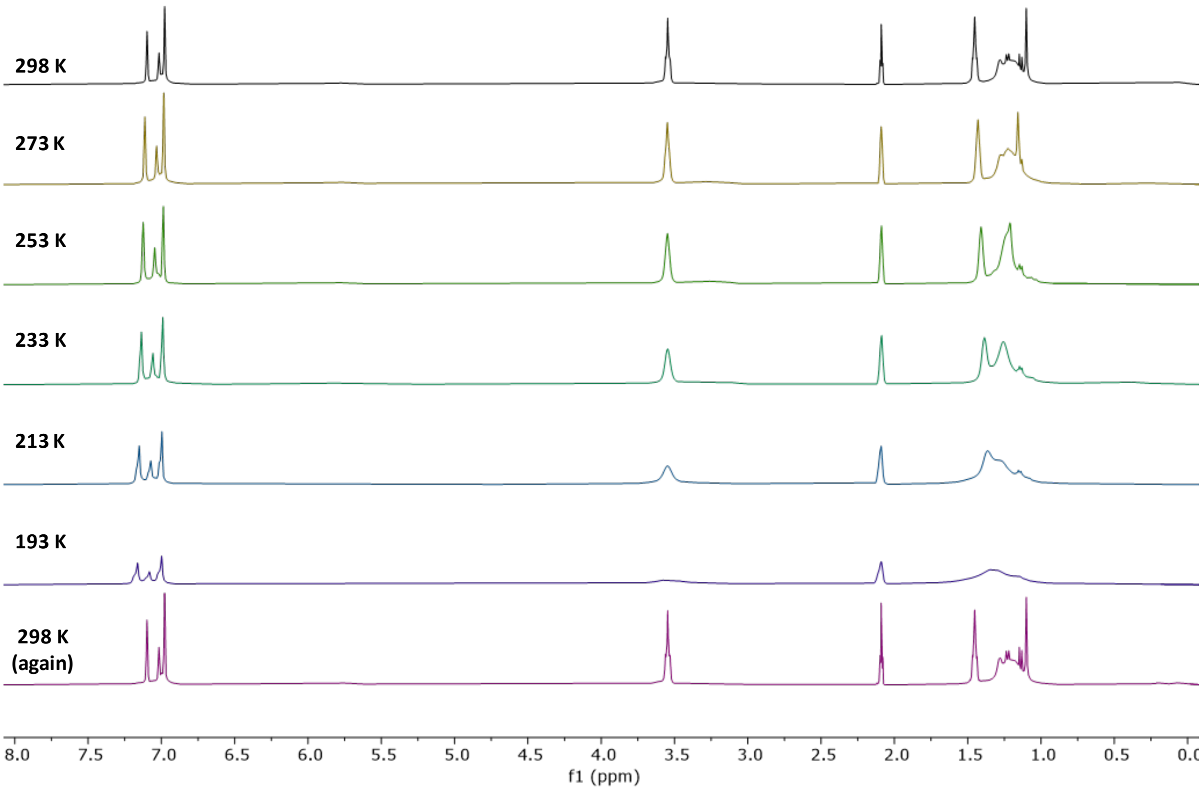

Figure S7: Stacked VT ^{1}H-NMR spectra of the orange film obtained by reacting **^{dipp}DAMN** with 2 equiv of KO*t*-Bu, recorded in toluene-d_8.

1.4 Synthesis of ^{dipp}DAMNK$_2$(18-c-6)·HO*t*-Bu:

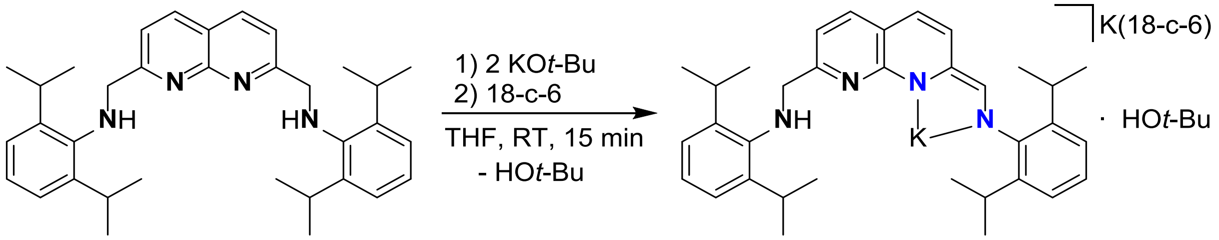

A suspension of KO*t*-Bu (30.9 mg, 275 µmol, 2.0 equiv) in THF (2 mL) was added dropwise to a vigorously stirred solution of ^{dipp}**DAMN** (70.0 mg, 138 µmol, 1.0 equiv) in THF (3 mL) at ambient temperature, yielding a dark-orange solution. To this mixture, a suspension of 18-crown-6 (36.4 mg, 138 µmol, 1.0 equiv) in THF (1.5 mL) was added dropwise, affording a dark-green solution. The mixture was stirred for 15 min and subsequently the solvent was evaporated *in vacuo*. The residue was stripped with pentane (2 mL) to remove residual THF, followed by freeze-drying from benzene (4.5 mL), yielding the title compound as a dark green powder (113.9 mg, 123 µmol, 89% yield).

^{1}H NMR (400 MHz, C_6D_6, 298 K): δ 7.22-7.19 (m, 4H*), 7.11-7.06 (m, 2H*), 6.34 (d, $^3J_{H,H}$ = 6.7 Hz, 1H), 6.02 (d, $^3J_{H,H}$ = 9.0 Hz, 1H), 5.90 (d, $^3J_{H,H}$ = 9.0 Hz, 1H), 5.78 (d, $^3J_{H,H}$ = 6.7 Hz, 1H), 5.46 (s, 1H), 4.84 (s, 1H), 3.98 (d, $^3J_{H,H}$ = 4.8 Hz, 2H), 3.78 (sept, $^3J_{H,H}$ = 6.8 Hz, 2H), 3.67 (sept, $^3J_{H,H}$ = 6.8 Hz, 2H), 3.25 (s, 24H), 1.41 (d, $^3J_{H,H}$ = 6.8 Hz, 12H*), 1.38 (d, $^3J_{H,H}$ = 6.8 Hz, 12H*), 1.35 (s*).

*Overlapping resonances

^{13}C{^{1}H}-NMR (APT, 101 MHz, C_6D_6, 298 K): δ 166.0, 157.0, 146.8, 143.9, 141.6, 140.2, 137.8, 128.1*, 127.4*, 123.9, 123.8, 122.8, 121.9, 121.7*,121.7*, 108.9, 103.5, 69.9, 66.9, 57.9, 35.3, 28.3*,28.3*, 24.7, 24.6.

*Overlapping resonances

ATR-IR (cm^{-1}): 3502 (w), 2958 (m), 2893 (w), 2867 (m), 1592 (m), 1567 (m), 1523 (m), 1440 (m), 1424 (m), 1351 (m), 1284 (m), 1249 (m), 1204 (m), 1157 (m), 1110 (s), 1061 (m), 962 (m), 927 (m), 753 (m).

Elemental analysis: Satisfactory elemental analysis results could not be obtained for this compound, most likely due to a small excess of HO*t*-Bu (*i.e.* more than 1 equiv) being retained due to the described dynamic exchange.

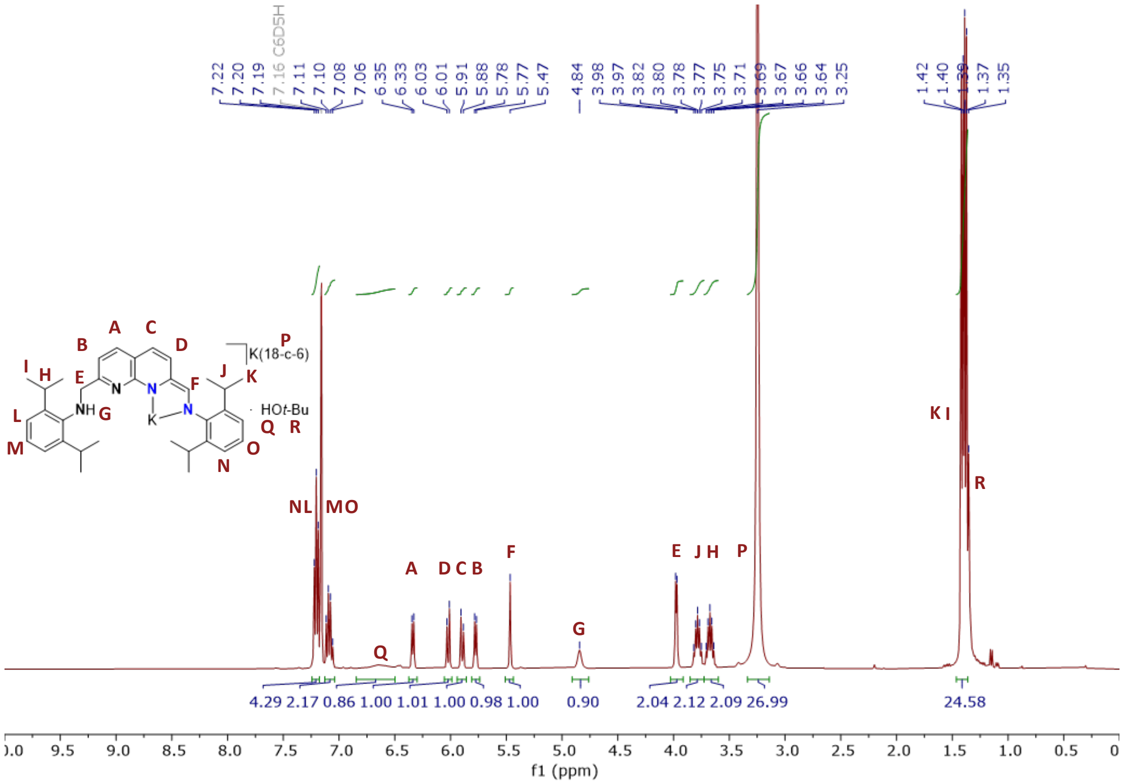

Figure S8: ^{1}H-NMR spectrum of **^{dipp}DAMN**K_2(18-c-6)·HO*t*-Bu in C_6D_6 at 298 K. The resonance marked with Q sharpens at lower temperatures.

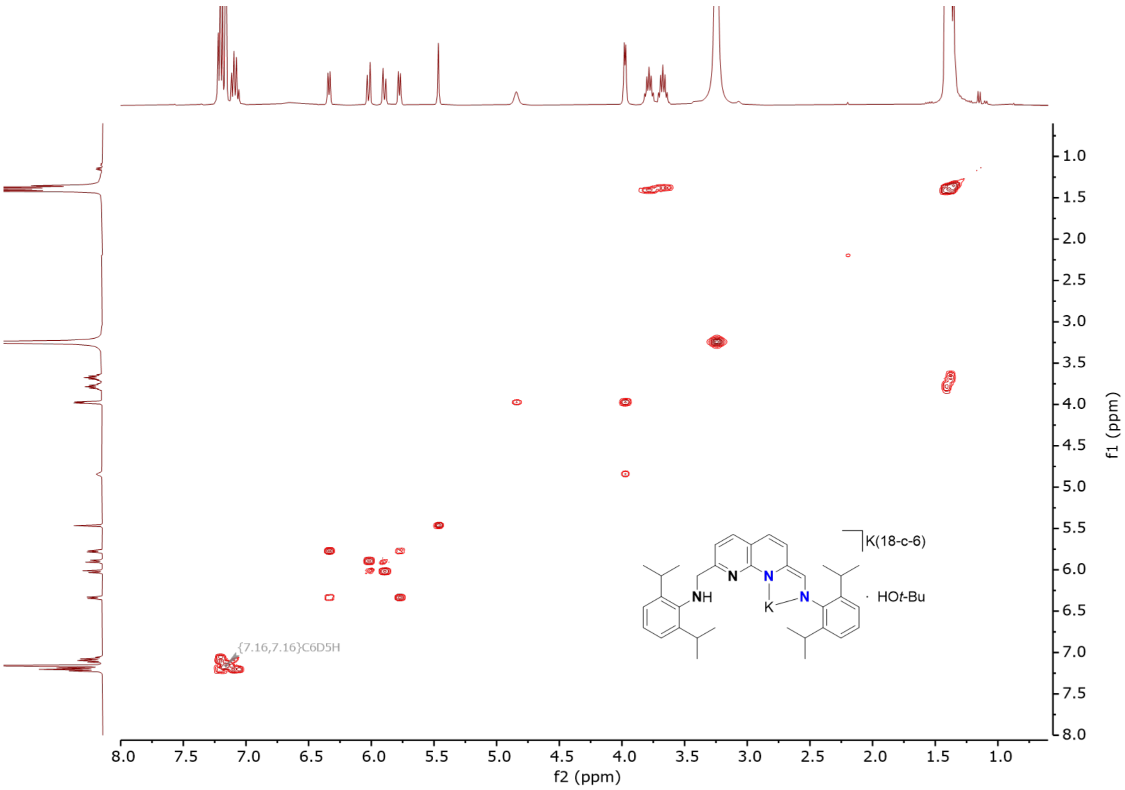

Figure S9: ^{1}H COSY spectrum of **^{dipp}DAMN**K_2(18-c-6)·HO*t*-Bu in C_6D_6 at 298 K.

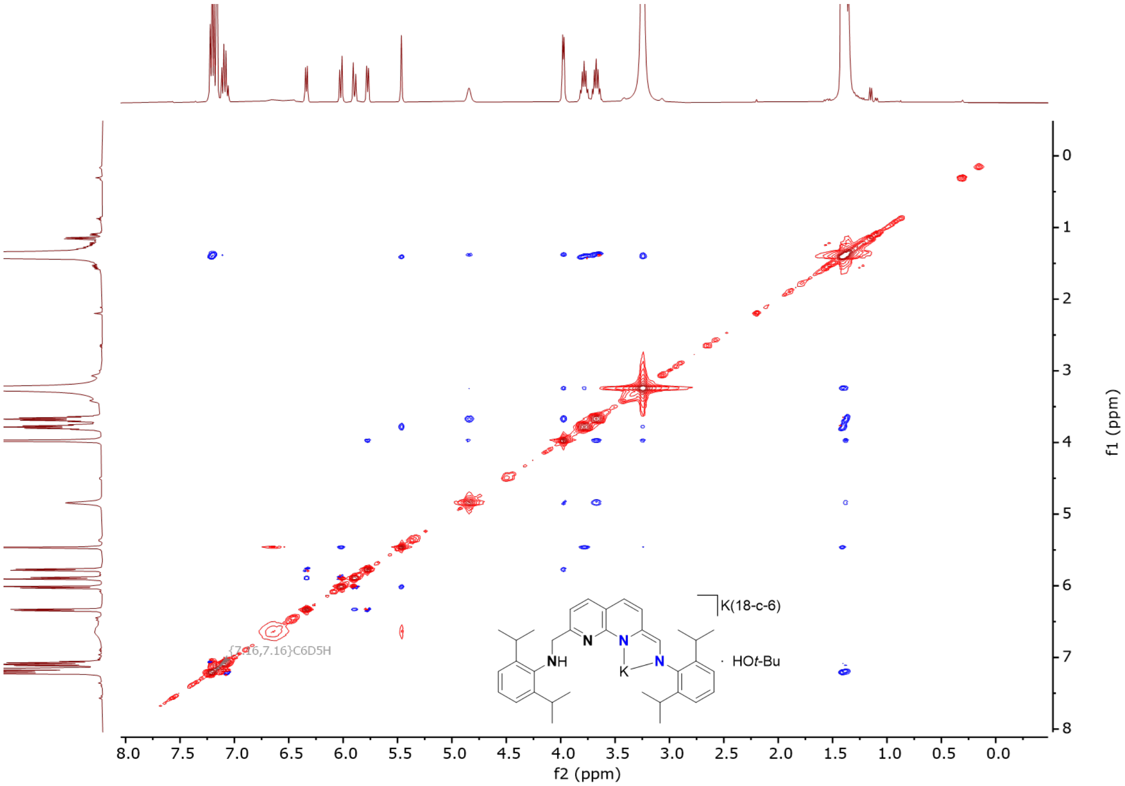

Figure S10: 2D-NOESY spectrum of **^{dipp}DAMN**K_2(18-c-6)·HO*t*-Bu in C_6D_6 at 298 K.

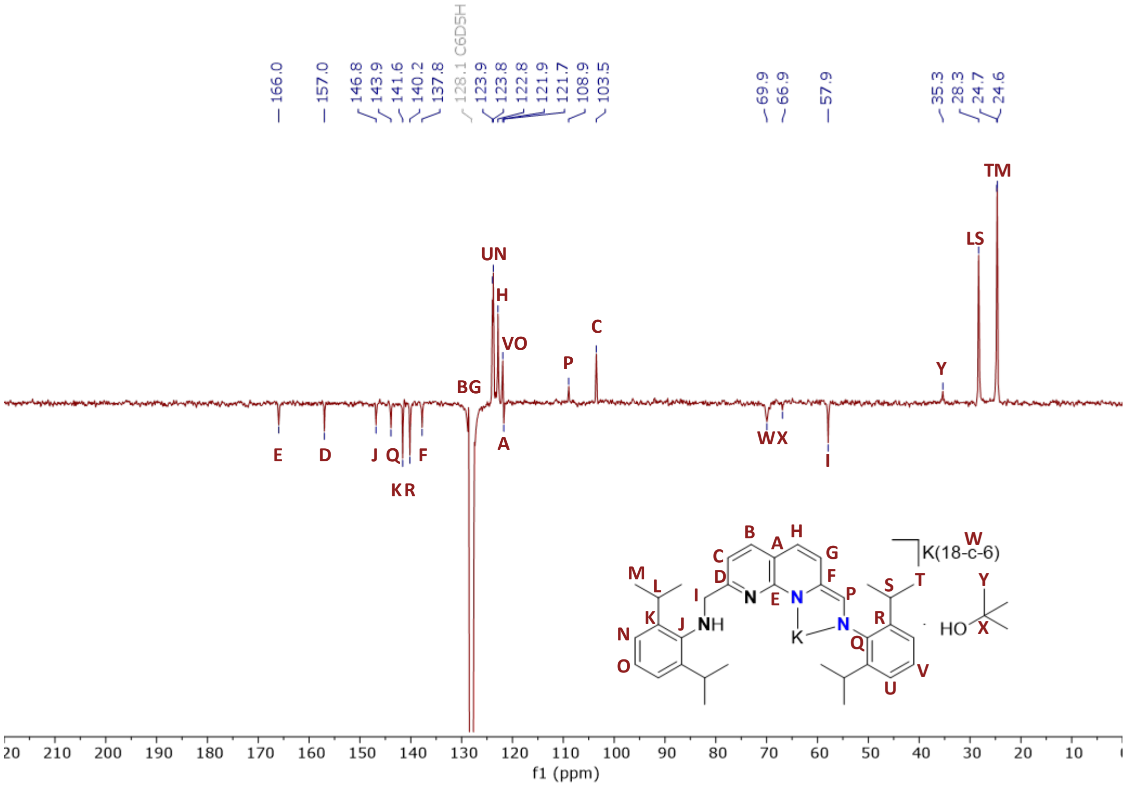

Figure S11: $^{13}C\{^{1}H\}$-NMR (APT) spectrum of **^{dipp}DAMN**K_2(18-c-6)·HO*t*-Bu in C_6D_6 at 298 K. The resonances marked with B and G are identified through heteronuclear 2D-NMR spectroscopy.

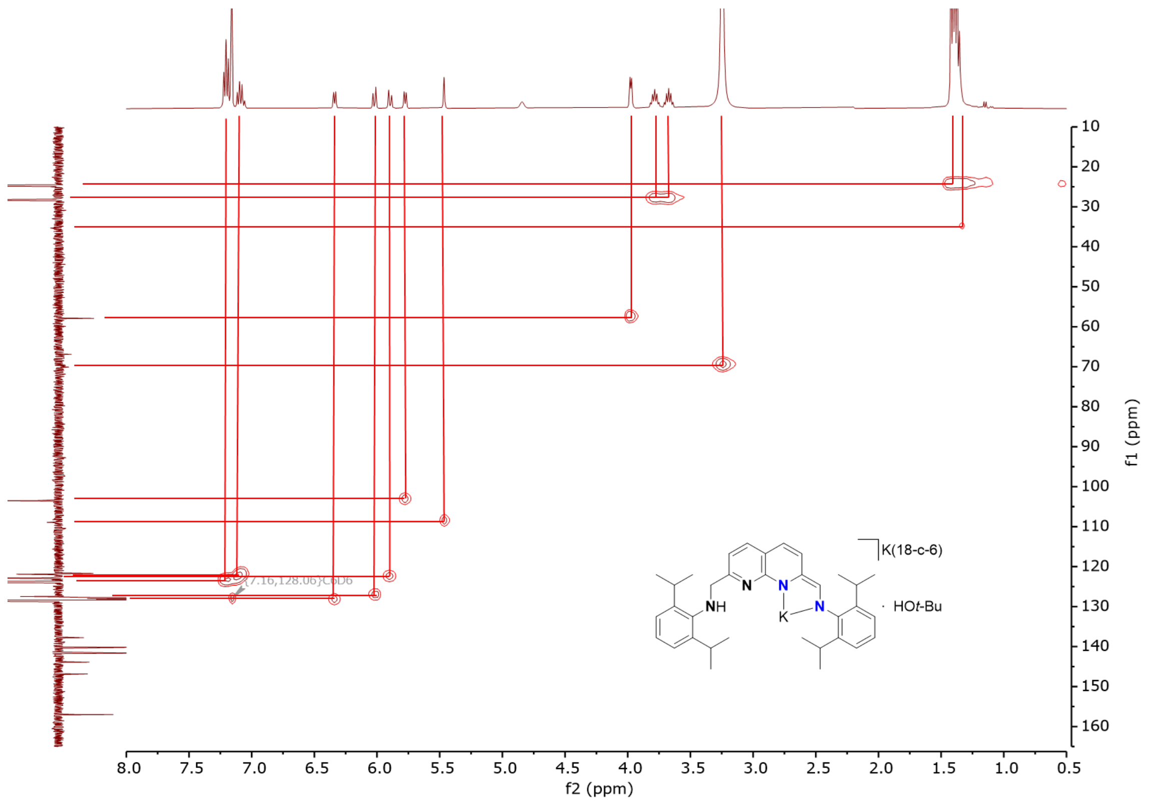

Figure S12: ^{1}H-^{13}C ASAP-HMQC NMR spectrum of **^{dipp}DAMN**K_2(18-c-6)·HO*t*-Bu in C_6D_6 at 298 K.

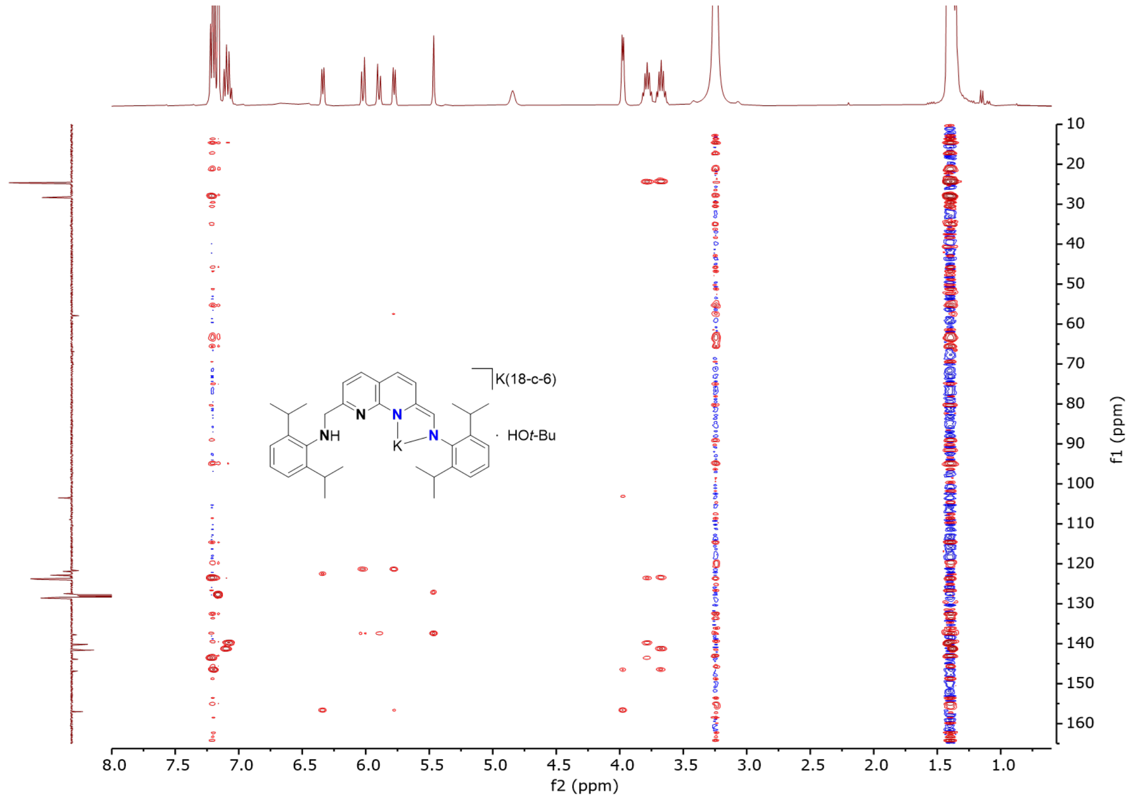

Figure S13: ^{1}H-^{13}C HMBC NMR spectrum of **^{dipp}DAMN**K_2(18-c-6)·HO*t*-Bu in C_6D_6 at 298 K.

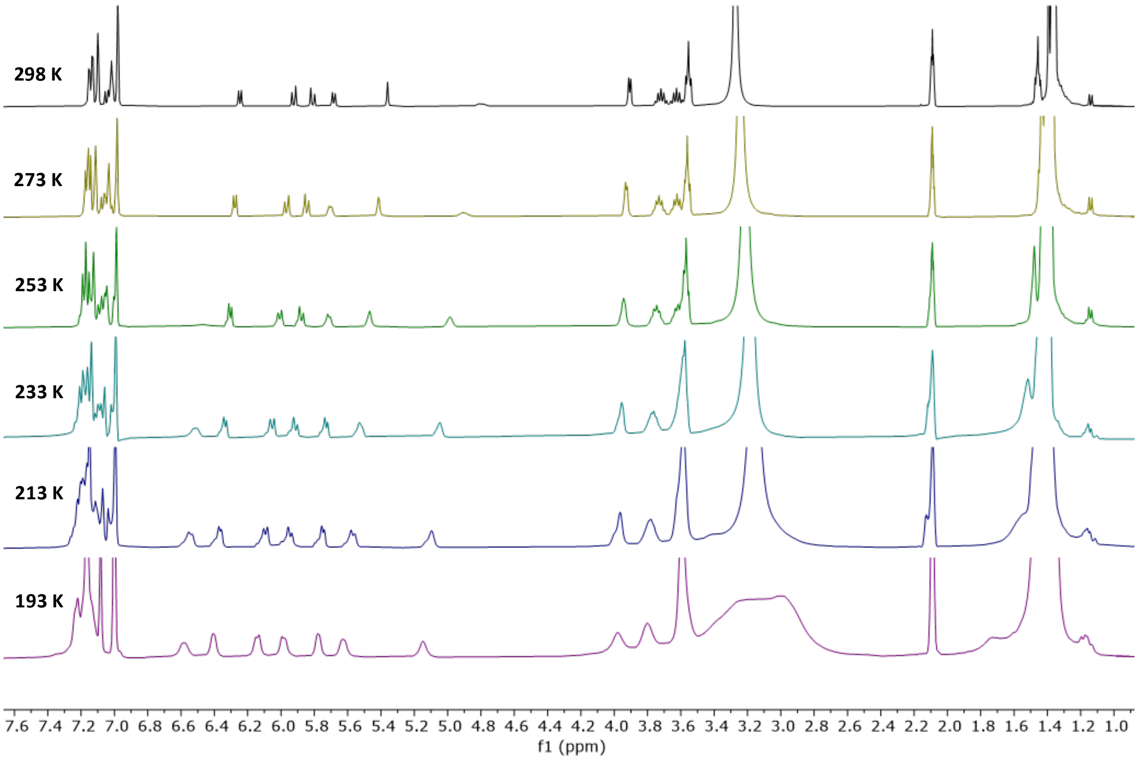

Figure S14: Stacked VT-NMR spectra of **^{dipp}DAMN**K_2(18-c-6)·HO*t*-Bu in toluene-d_8.

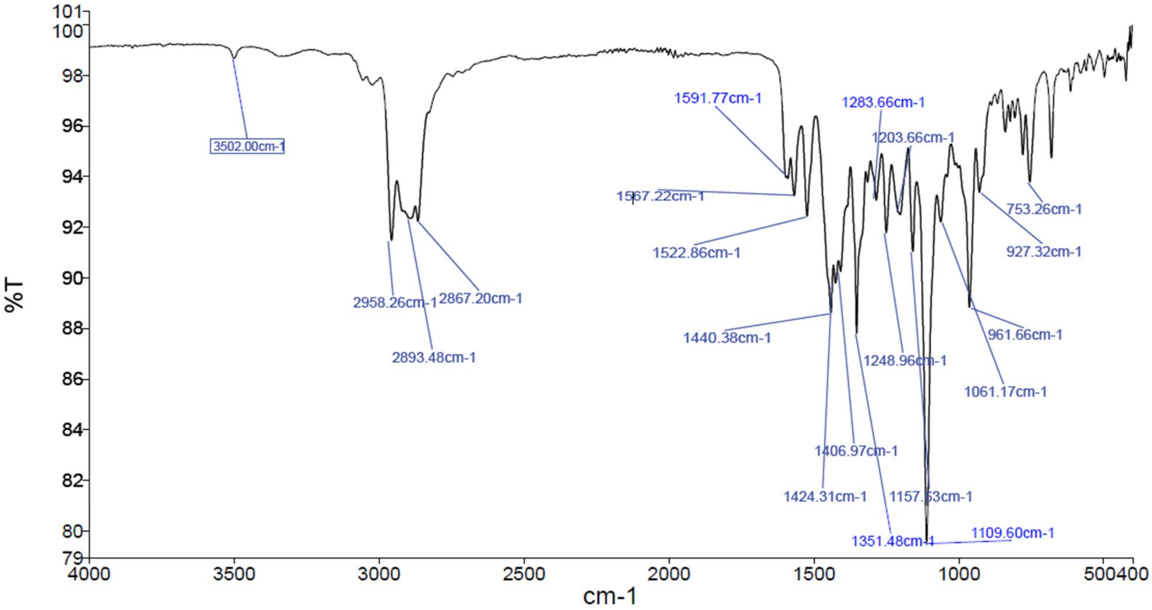

Figure S15: ATR-IR spectrum of **^{dipp}DAMN**K_2(18-c-6)·HO*t*-Bu measured as a film under N_2 flow at 298 K.

1.5 Synthesis of ^{dipp}NDC:

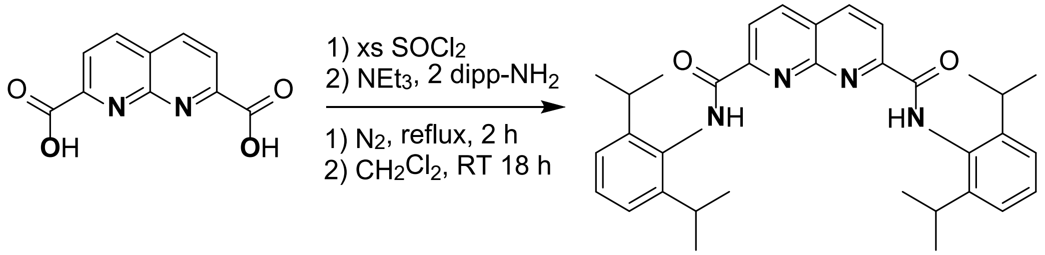

Under an N_2 atmosphere, 1,8-naphthyridine-2,7-dicarboxylic acid (1.00 g, 4.58 mmol 1.00 equiv.) was suspended in $SOCl_2$ (17.5 mL), yielding a yellow suspension. After refluxing the mixture for 2 h a dark orange solution was obtained. The mixture was allowed to cool down and unreacted $SOCl_2$ was removed under vacuum. Subsequently, CH_2Cl_2 (100 mL), NEt_3 (1.8 mL, 12.9 mmol) and 2,6-diisopropylaniline (1.8 mL, 9.5 mmol, 2.1 equiv) were added to the yellow coloured solid, yielding a dark green solution. The solution was stirred at ambient temperature for 18 h and subsequently the volatiles were removed under vacuum to give a green coloured solid. Purification by column chromatography (SiO_2, CH_2Cl_2:PE, 1:1, R_f = 0.3) gave the title compound as an off-white coloured solid (1.88 g, 76%).

^{1}H-NMR (400 MHz, C_6D_6, 298 K): δ 9.76 (s, 2H), 8.56 (d, $^3J_{H,H}$ = 8.3 Hz, 2H), 7.69 (d, $^3J_{H,H}$ = 8.3 Hz, 2H), 7.24 (t, $^3J_{H,H}$ = 7.5 Hz, 2H), 7.13 (d, $^3J_{H,H}$ = 7.5 Hz, 4H), 3.33 (sept, $^3J_{H,H}$ = 6.9 Hz, 4H), 1.19 (d, $^3J_{H,H}$ = 6.9 Hz, 24H).

^{13}C-APT NMR (101 MHz, C_6D_6, 298 K): δ 163.0, 153.9, 153.0, 146.4, 139.3, 131.4, 128.5, 125.5, 123.5, 121.1, 29.2, 23.6.

ATR-IR (cm^{-1}): 3352 (w), 3295 (m), 2960 (m), 2869 (w), 1681 (s), 1595 (w), 1684 (s), 1483 (s), 1419 (w), 1385 (w), 1363 (w), 1133 (w), 1119 (w), 876 (w), 798 (w), 790 (w), 734(w).

Elemental Analysis: Calcd. For $C_{34}H_{40}N_4O_2$: C, 76.09; H, 7.51; N, 10.43. Found C, 75.92; H, 7.51; N, 10.32.

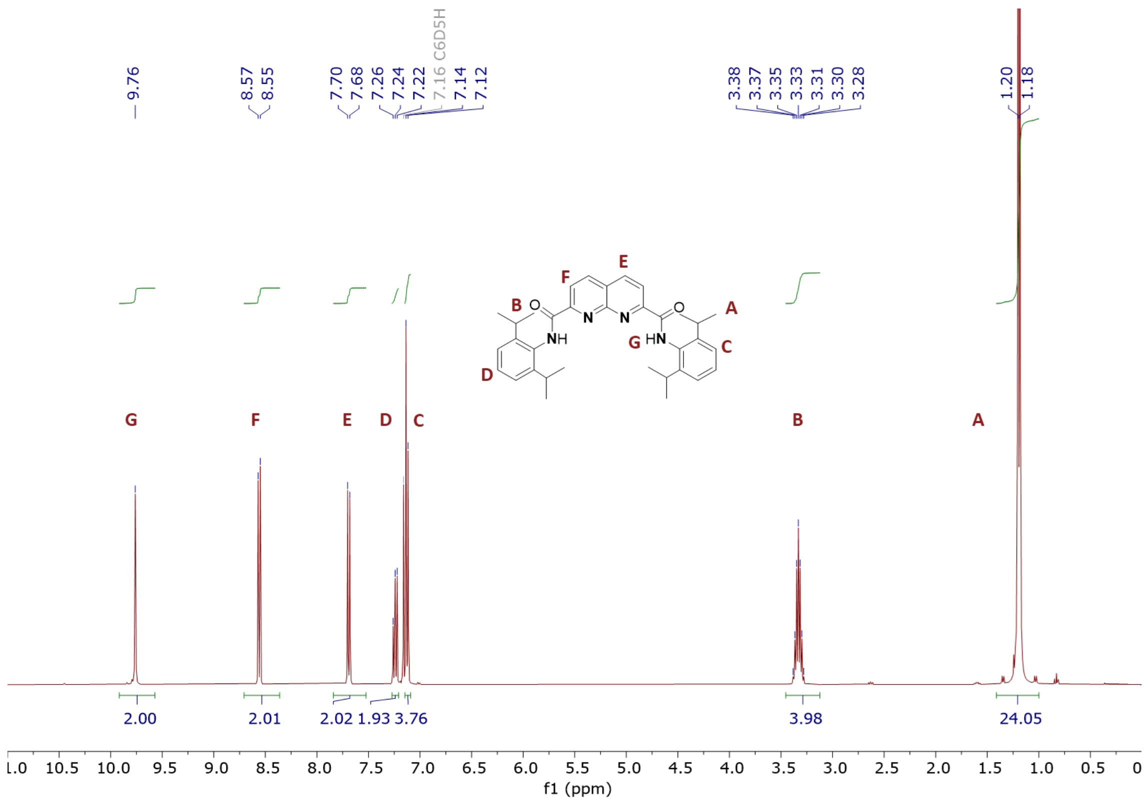

Figure S16: ^{1}H-NMR spectrum of **dippNDC** in C_6D_6 at 298 K.

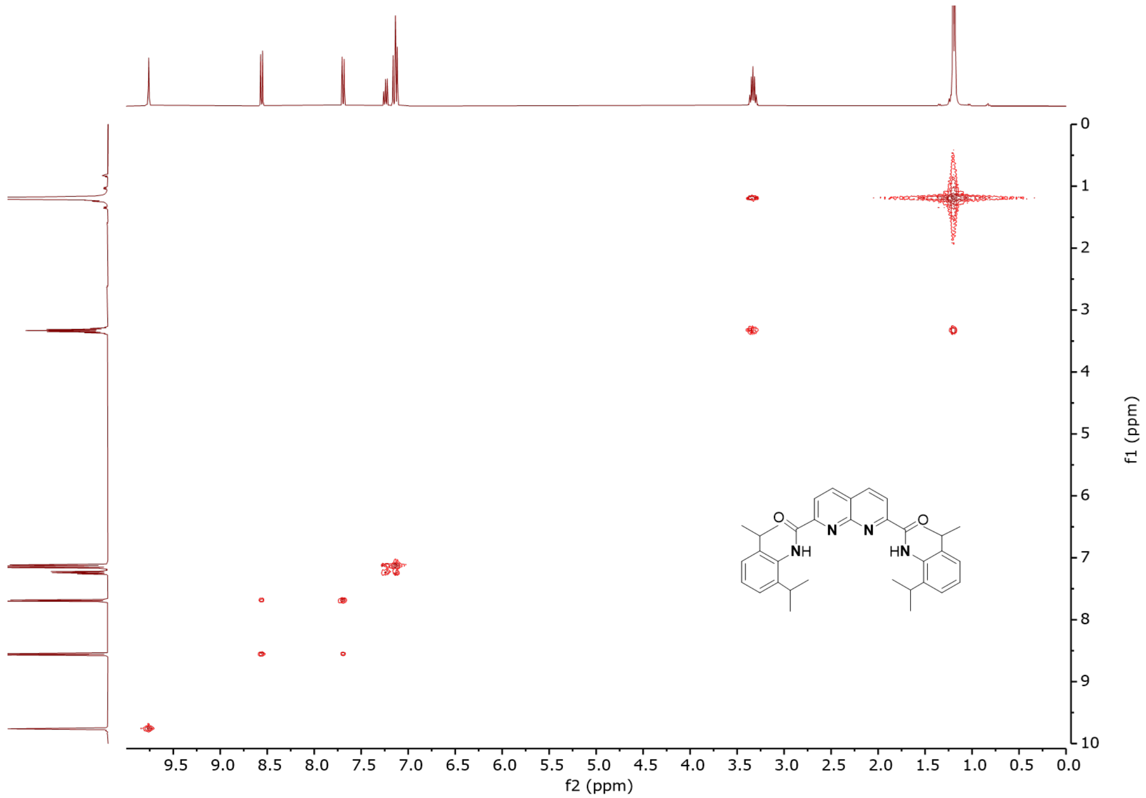

Figure S17: ^{1}H COSY NMR spectrum of **dippNDC** in C_6D_6 at 298 K.

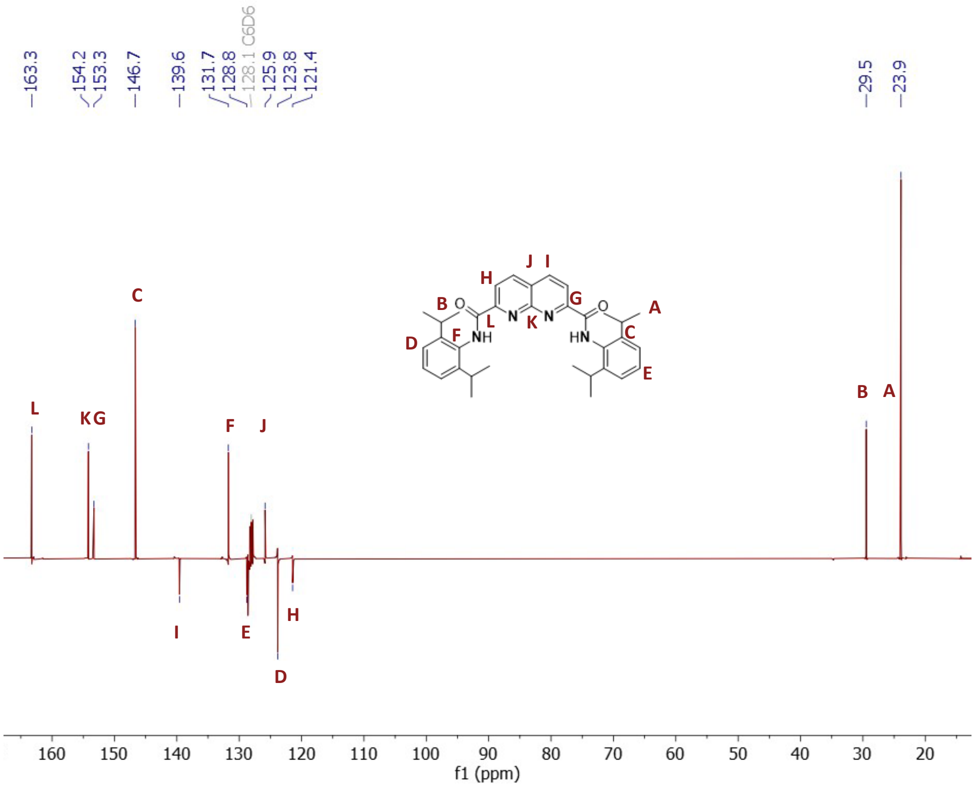

Figure S18: $^{13}C\{^{1}H\}$-NMR (APT) spectrum of **^{dipp}NDC** in C_6D_6 at 298 K. Note: A phasing error occurred when recording this spectrum, affecting resonances A and B.

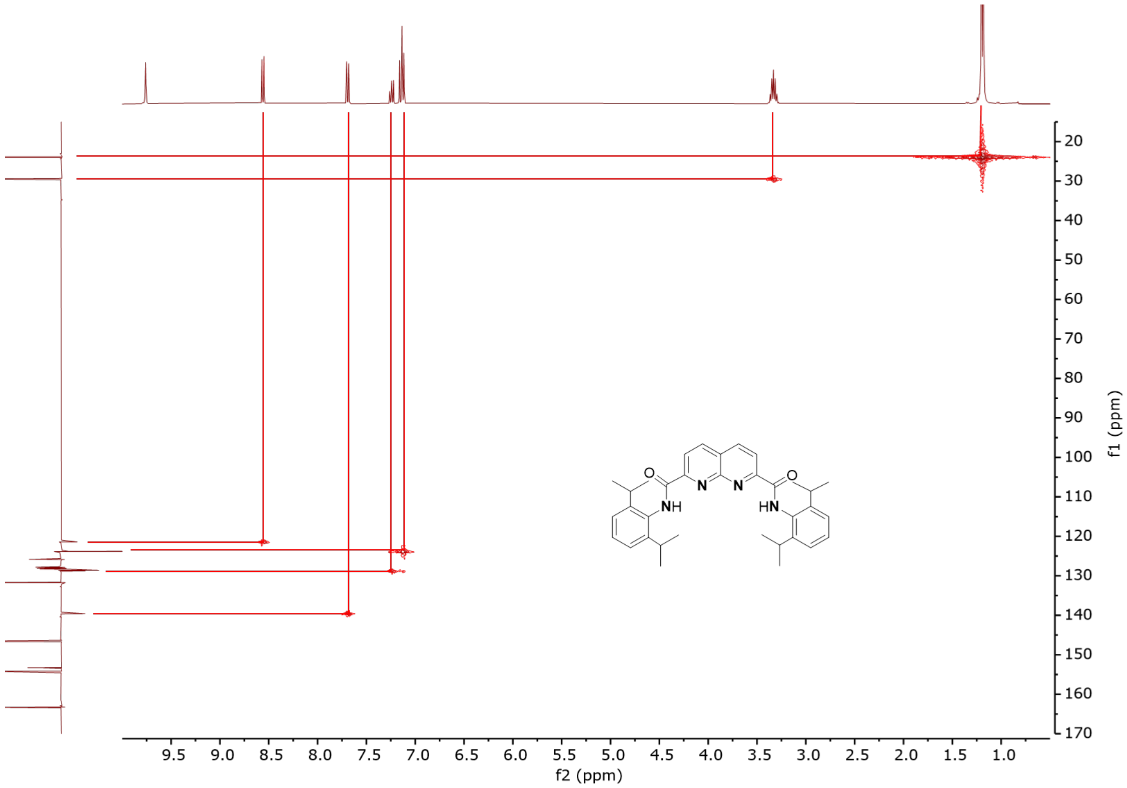

Figure S19: ^{1}H-^{13}C HMQC NMR spectrum of **^{dipp}NDC** in C_6D_6 at 298 K.

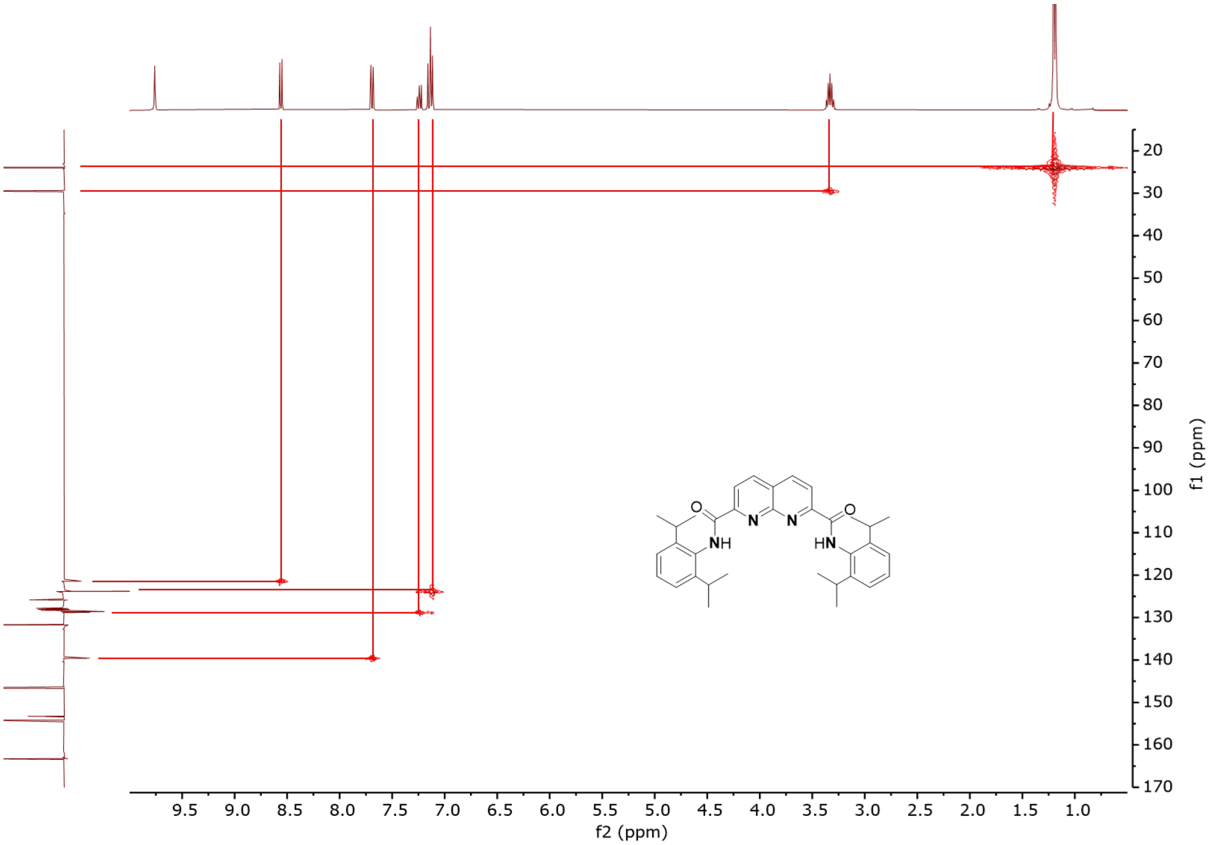

Figure S20: ^{1}H-^{13}C HMBC NMR spectrum of **^{dipp}NDC** in C_6D_6 at 298 K.

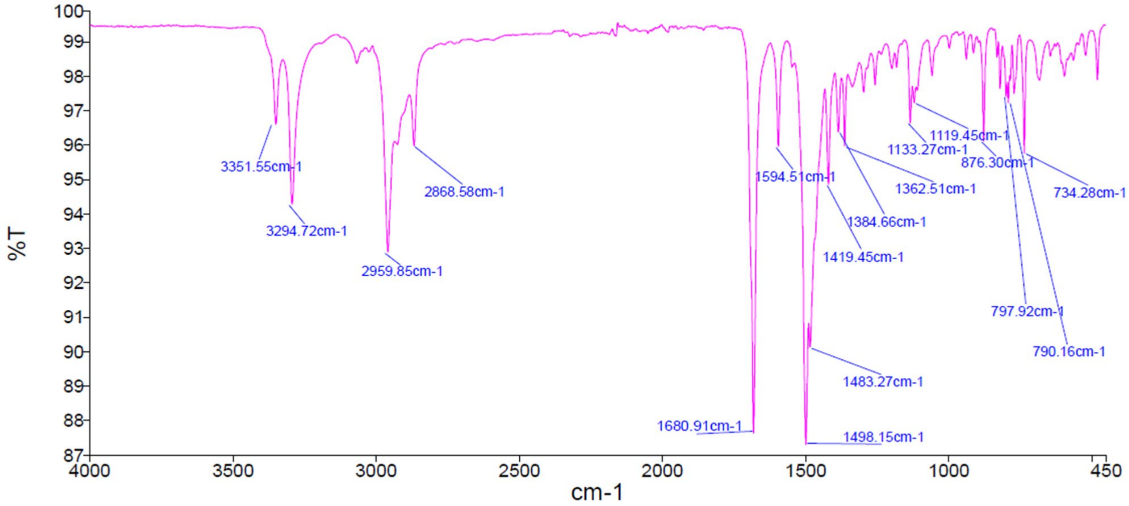

Figure S21: ATR-IR spectrum of **^{dipp}NDC** measured at 298 K.

1.6 Synthesis of ^{dipp}NDCK$_2$·2(18-c-6):

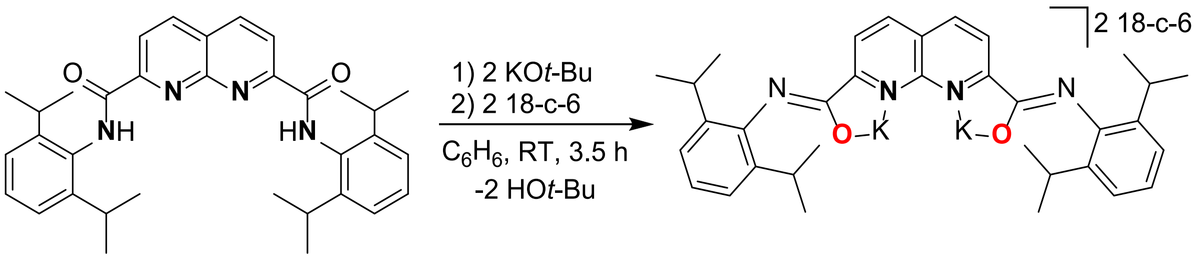

A suspension of KO*t*-Bu (4.2 mg, 37.3 µmol, 2.0 equiv) in benzene (1.5 mL) was added slowly to a vigorously stirred solution of ^{dipp}**NDC** (10.0 mg, 18.6 µmol, 1.0 equiv) in benzene (1.5 mL) at ambient temperature, yielding a light-yellow solution. After 30 min a solution of 18-crown-6 in benzene (1.5 mL) was added, turning the solution darker yellow. After 3 h of stirring at ambient temperature the solvent was removed under vacuum and the resulting yellow microcrystalline solid was washed with pentane (3 x 2 mL) and dried to give the title compound as a light-yellow solid (18.6 mg, 16.3 µmol, 88% yield). Single crystals suitable for analysis by X-ray diffraction were grown by layering a saturated toluene solution with pentane.

^{1}H NMR (400 MHz, C_6D_6, 298 K): δ 9.16 (d, $^{3}J_{H,H}$ = 8.2 Hz, 2H), 7.21 (d, $^{3}J_{H,H}$ = 8.2 Hz, 2H), 7.43 (d, $^{3}J_{H,H}$ = 7.5 Hz, 4H), 7.25 (t, $^{3}J_{H,H}$ = 7.5 Hz, 2H), 3.97 (sept, $^{3}J_{H,H}$ = 6.8 Hz, 4H), 3.21 (s, 48 H), 1.61 (d, $^{3}J_{H,H}$ = 6.8 Hz, 24H).

^{13}C{^{1}H}-NMR (APT, 101 MHz, C_6D_6, 298 K): δ 165.9, 162.9, 157.3, 152.2, 141.0, 134.4, 124.1, 122.0, 121.5, 120.1, 70.1, 29.2, 24.3.

ATR-IR (cm^{-1}): 3049 (w), 2951 (m), 2888 (s), 2864 (s), 1593 (m), 1563 (s), 1525 (w), 1470 (w), 1430 (m), 1401 (w), 1352 (m), 1290 (w), 1250 (w), 1110 (s), 961 (w), 886 (w), 838 (w), 783 (w), 741 (w).

Elemental Analysis: The reactive nature of the compound prevented obtaining a satisfactory elemental analysis.

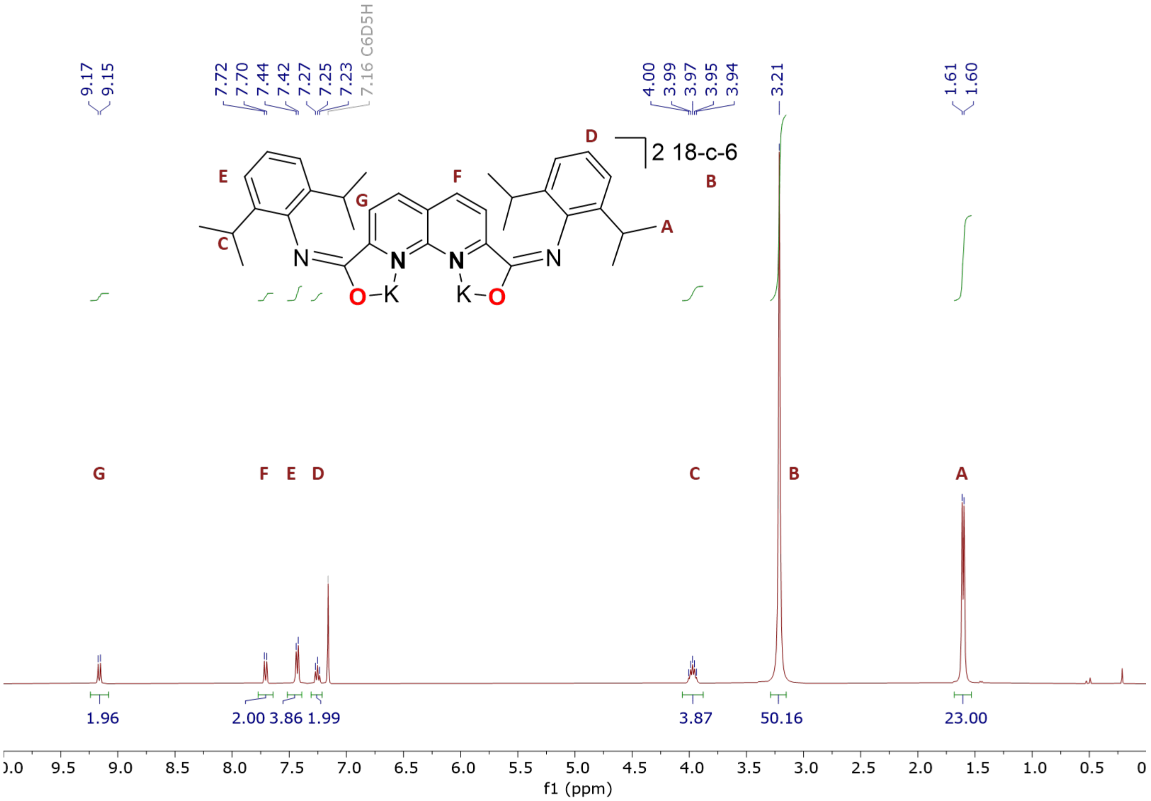

Figure S22: ^{1}H-NMR spectrum of **^{dipp}NDC**K_2·2(18-c-6) in C_6D_6 at 298 K.

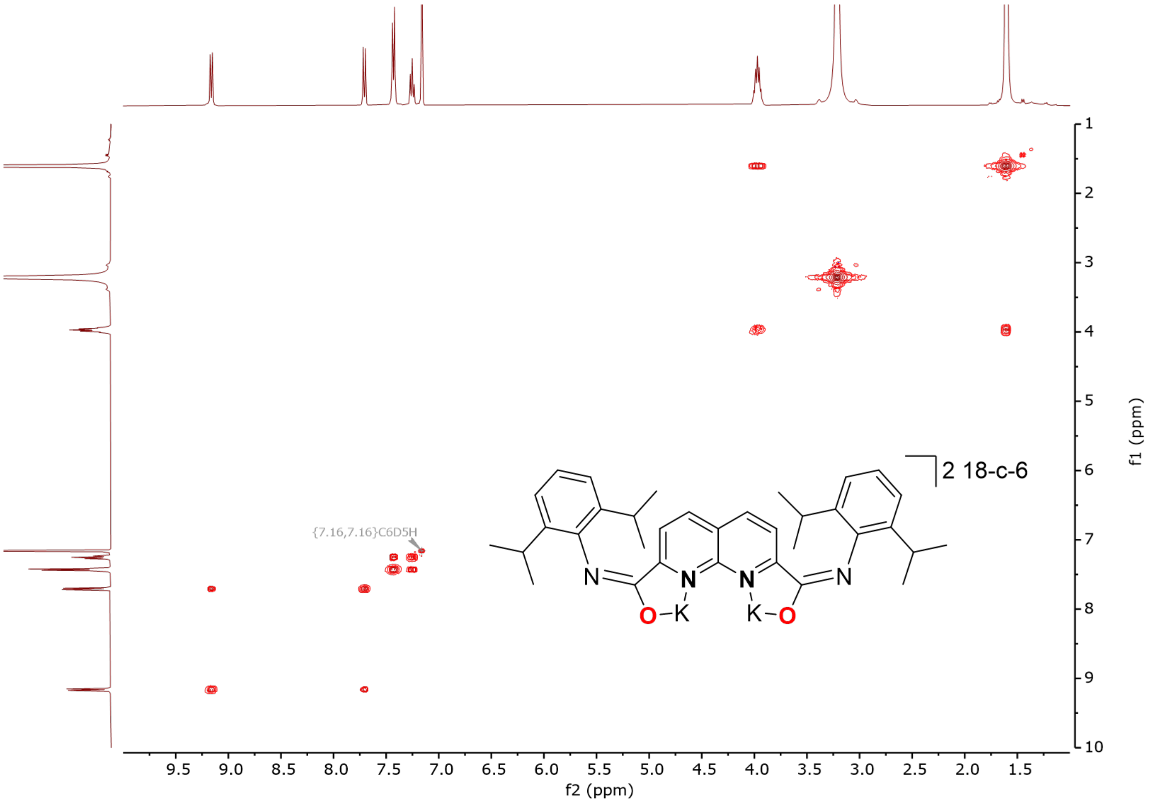

Figure S23: ^{1}H COSY NMR spectrum of **^{dipp}NDC**K_2·2(18-c-6) in C_6D_6 at 298 K.

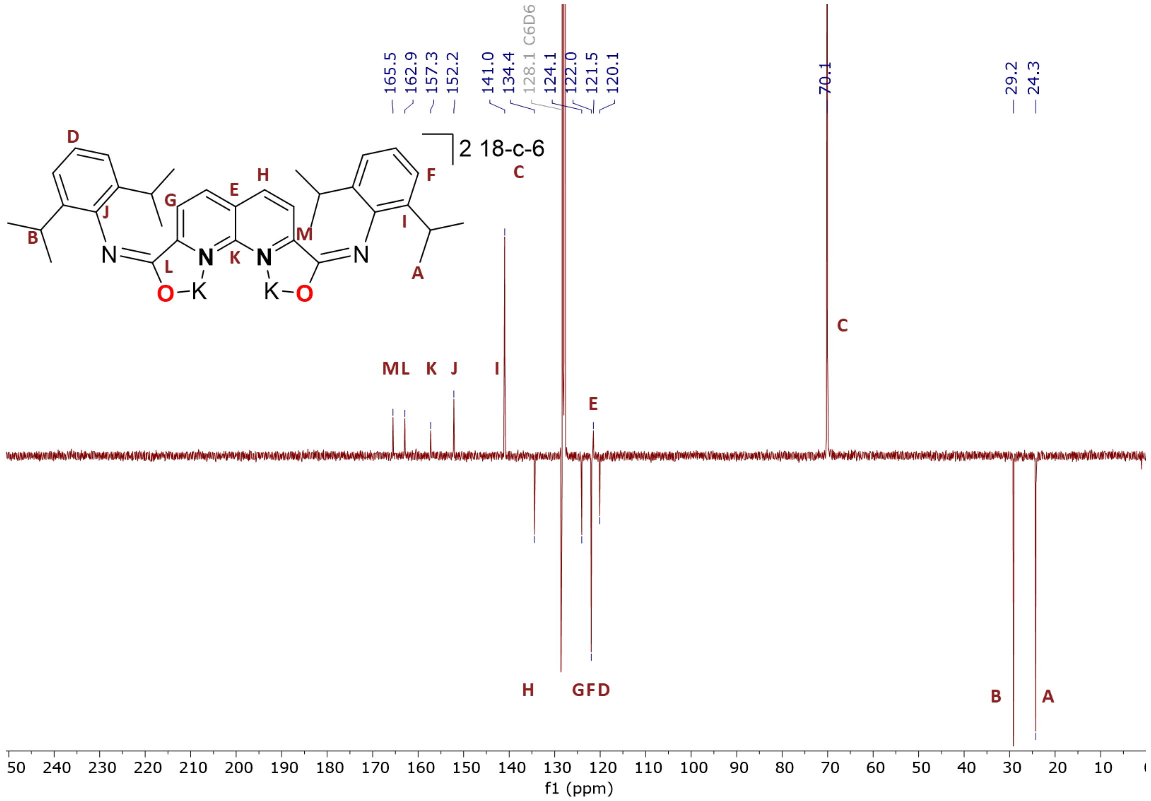

Figure S24: $^{13}C\{^{1}H\}$-NMR (APT) spectrum of **^{dipp}NDC**K_2·2(18-c-6) in C_6D_6 at 298 K.

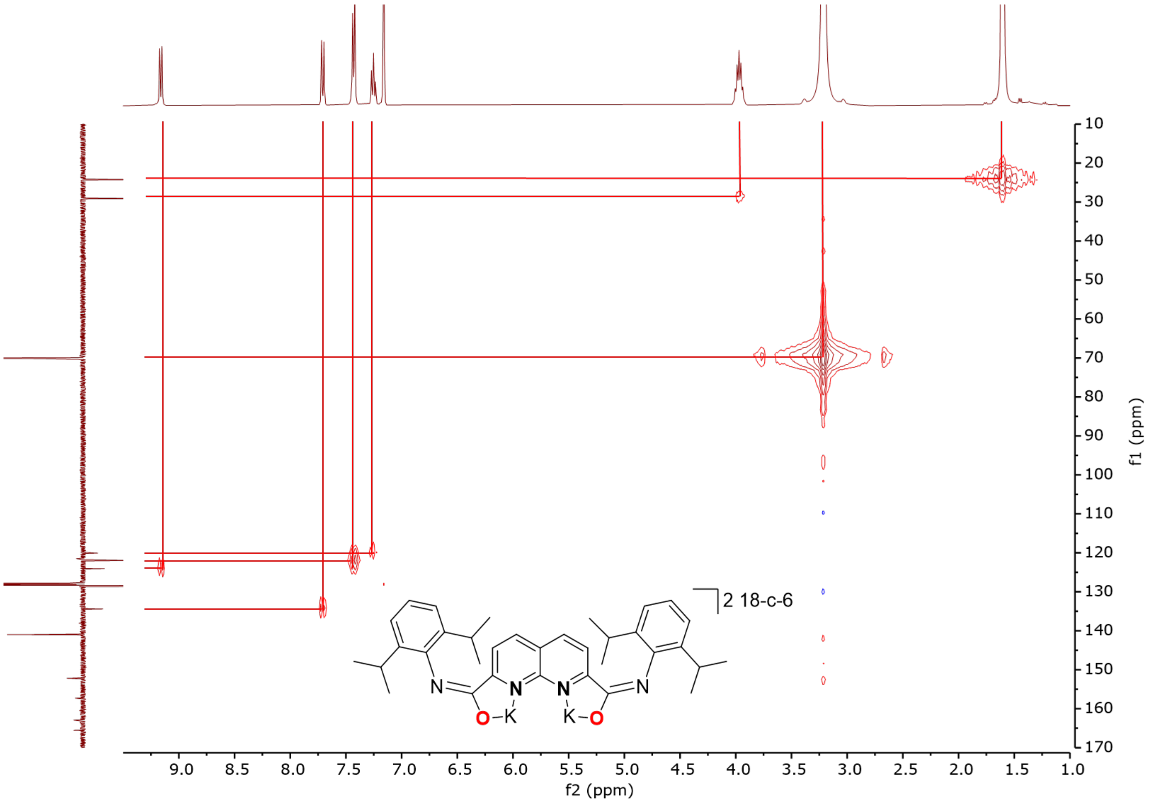

Figure S25: ^{1}H-^{13}C ASAP-HMQC NMR spectrum of **^{dipp}NDC**K_2·2(18-c-6) in C_6D_6 at 298 K.

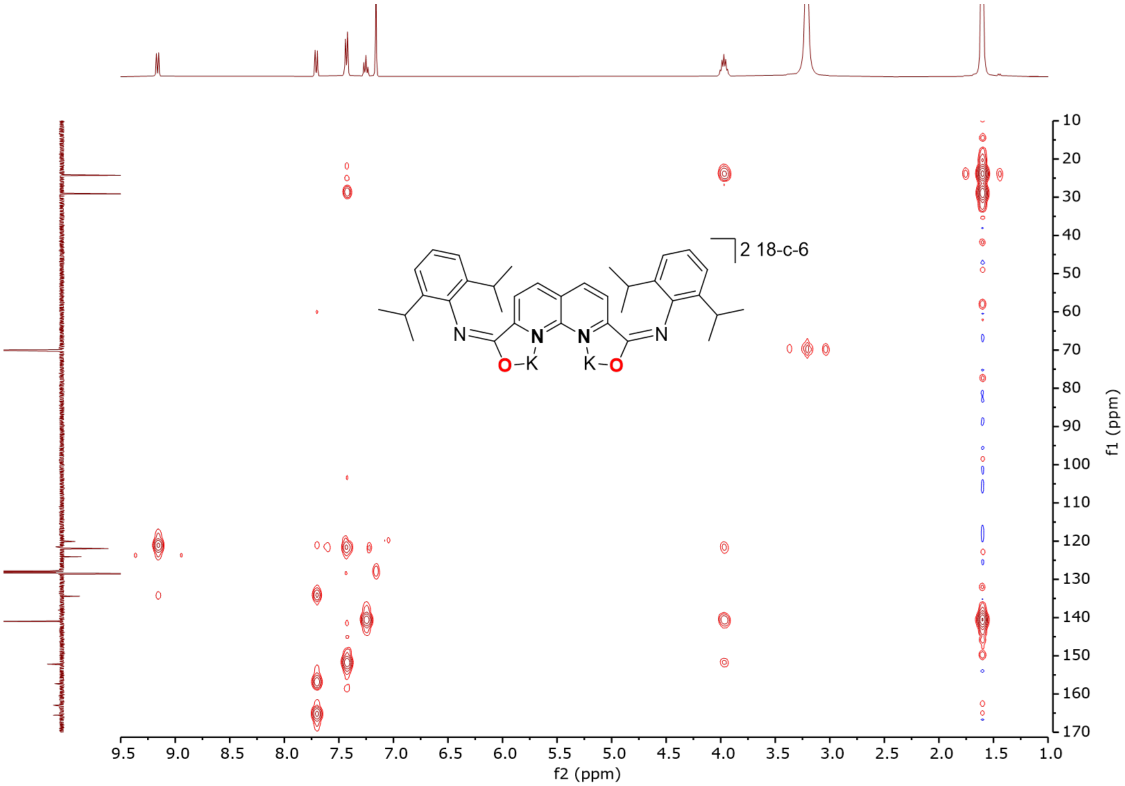

Figure S26: ^{1}H-^{13}C HMBC NMR spectrum of ^{dipp}**NDC**K_2·2(18-c-6) in C_6D_6 at 298 K.

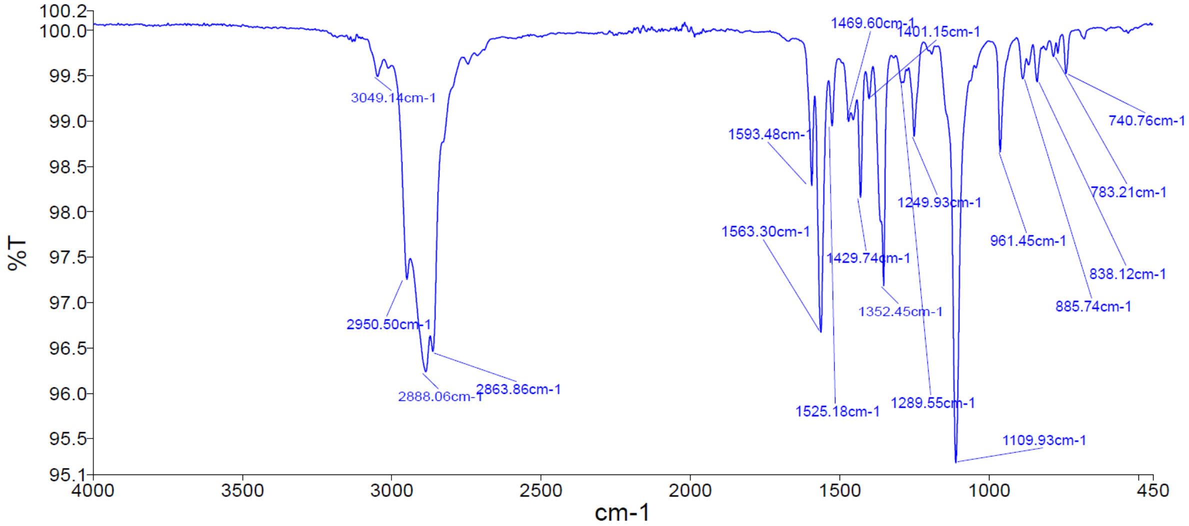

Figure S27: ATR-IR spectrum of ^{dipp}**NDC**K_2·2(18-c-6) measured as a film under N_2 flow at 298 K.

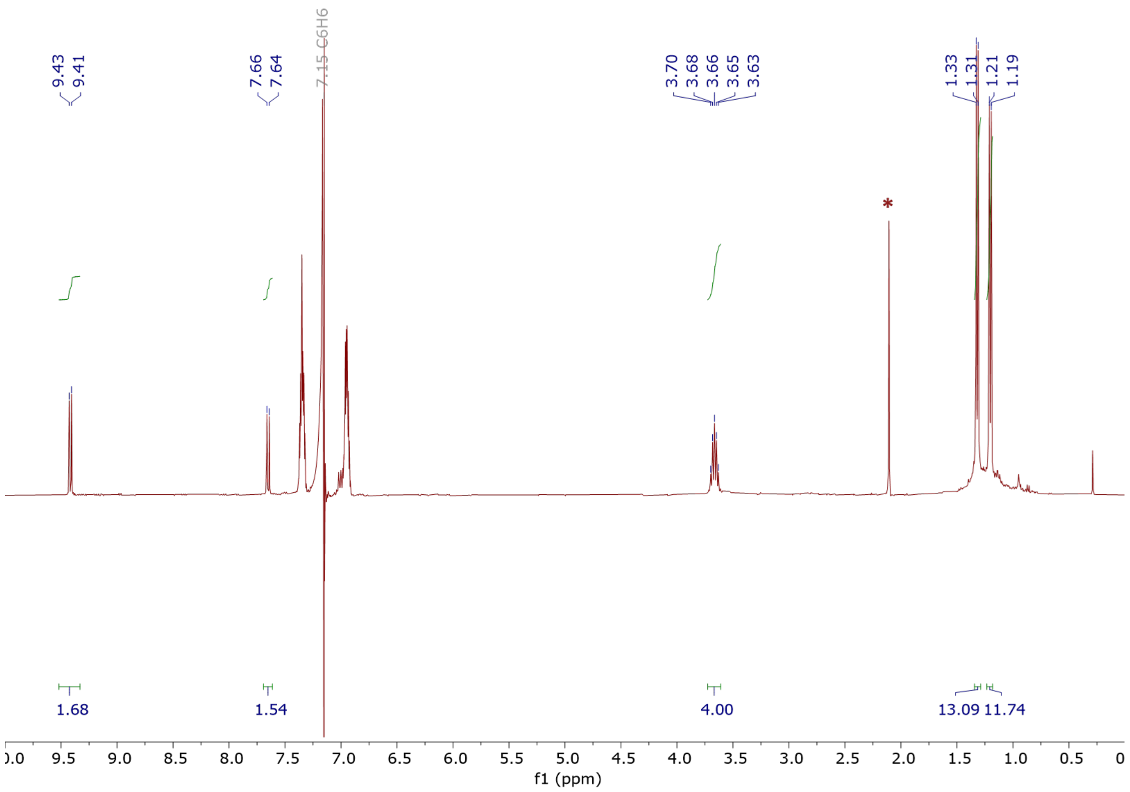

Figure S28: ^{1}H-NMR spectrum with PRESAT solvent suppression of the reaction mixture of ^{dipp}**NDC**K_2 (out of KBn), in C_6H_6 at 298 K. The resonance marked with a * is attributed to toluene.

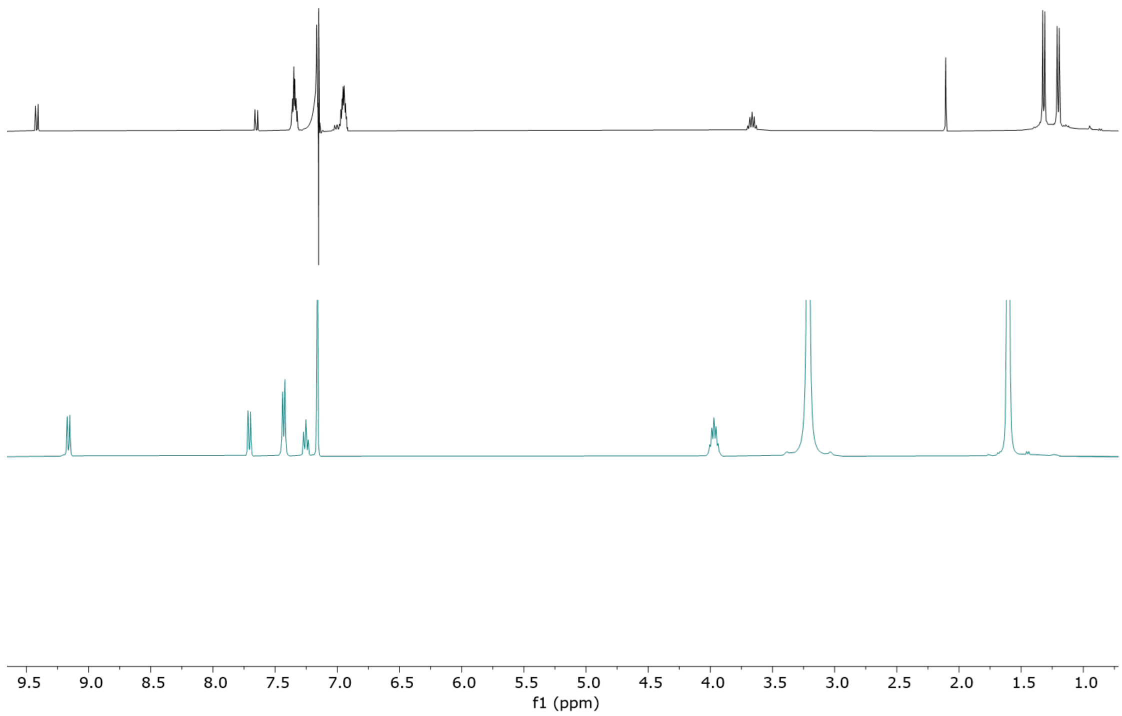

Figure S29: Stacked ^{1}H-NMR spectra of (top) ^{dipp}**NDC**K_2 in C_6H_6 and (bottom) ^{dipp}**NDC**K_2·2(18-c-6) in C_6D_6. Spectra recorded at 298 K.

1.7 Synthesis of ^{dipp}NDCMg$_2$Cl$_2$·4 THF:

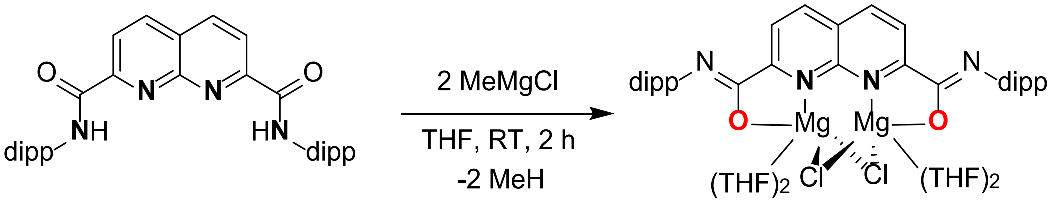

A solution of **^{dipp}NDC** (50.0 mg, 93 µmol, 1.0 equiv.) in THF (1 mL) was added dropwise to a colourless stirring solution of CH_3MgCl (3.06 M in THF, 60.9 µL, 186 µmol, 2.0 equiv.) in THF (1 mL), resulting in an instant colour change to a bright orange solution. After stirring the reaction mixture at ambient temperature for 2 h the solvent was removed under a dynamic vacuum to give a yellow-coloured solid. The solid was washed with hexane (3 x 2 mL) and dried extensively *in vacuo* to yield the title compound as a yellow solid. (65.0 mg, 74%). Single crystals suitable for analysis by X-ray diffraction were grown by layering a saturated solution of the compound in THF with hexane at -40 °C.

^{1}H-NMR (400 MHz, THF-d_8, 298 K): δ 8.90 (d, $^3J_{H,H}$ = 8.4 Hz, 2H), 8.54 (d, $^3J_{H,H}$ = 8.4 Hz, 2H), 6.99 (d, $^3J_{H,H}$ = 7.6 Hz, 4H), 6.83 (t, $^3J_{H,H}$ = 7.6 Hz, 2H), 3.28 (sept, $^3J_{H,H}$ = 6.9 Hz, 4H), 1.16 (d, $^3J_{H,H}$ = 6.9 Hz, 24H).

^{13}C-APT NMR (101 MHz, THF-d_8, 298 K): δ 162.3, 159.2, 152.7, 149.0, 139.9, 139.3, 124.8, 123.8, 122.4, 122.0, 29.3, 24.2.

ATR-IR (cm^{-1}): 3058 (w), 2959 (s), 2868 (m), 1648 (w), 1608 (m), 1584 (m), 1545 (w), 1503 (w), 1460 (w), 1435 (w), 1364 (m), 1293 (w), 1257 (w), 1206 (w), 1035 (m), 883 (w), 744 (w).

Elemental Analysis: The reactive nature of the compound prevented obtaining a satisfactory elemental analysis.

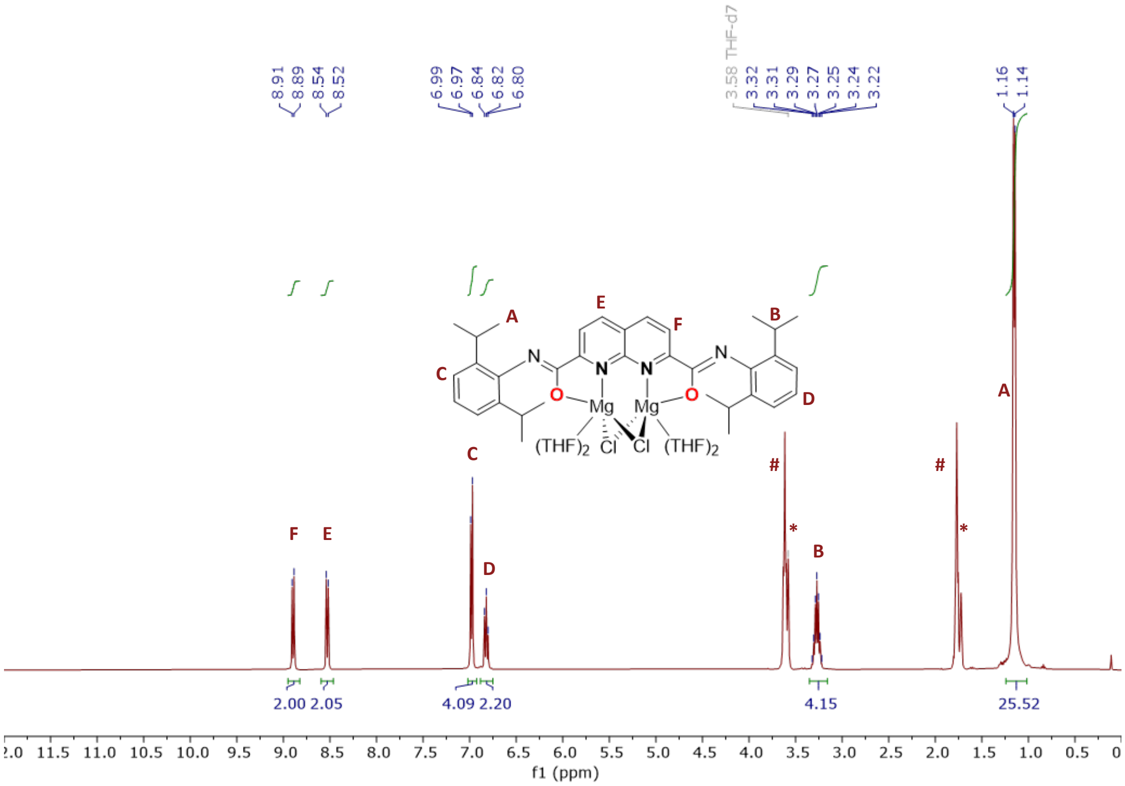

Figure S30: ^{1}H-NMR spectrum of **^{dipp}NDC**Mg_2Cl_2·4 THF in THF-d_8 at 298 K. Resonances marked with # are attributed to (bound) THF-H_8 and resonances marked with * are attributed to THF-d_7.

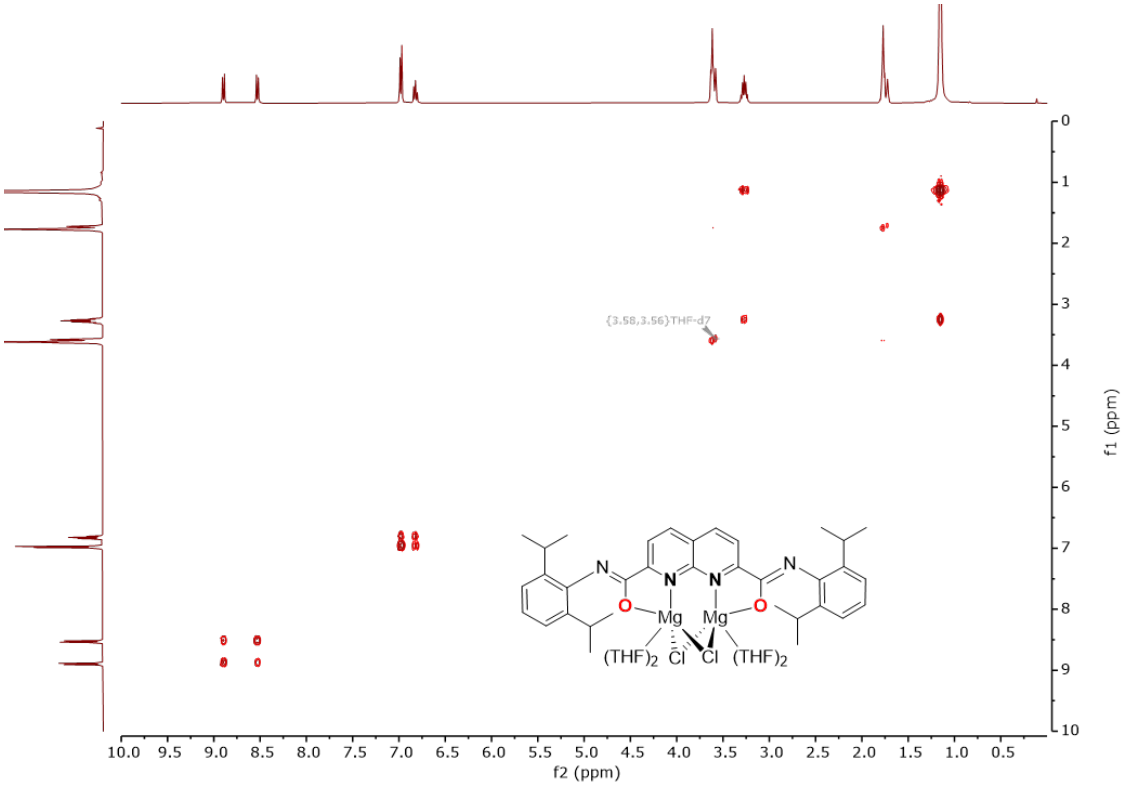

Figure S31: ^{1}H COSY NMR spectrum of **^{dipp}NDC**Mg_2Cl_2·4 THF in THF-d_8 at 298 K.

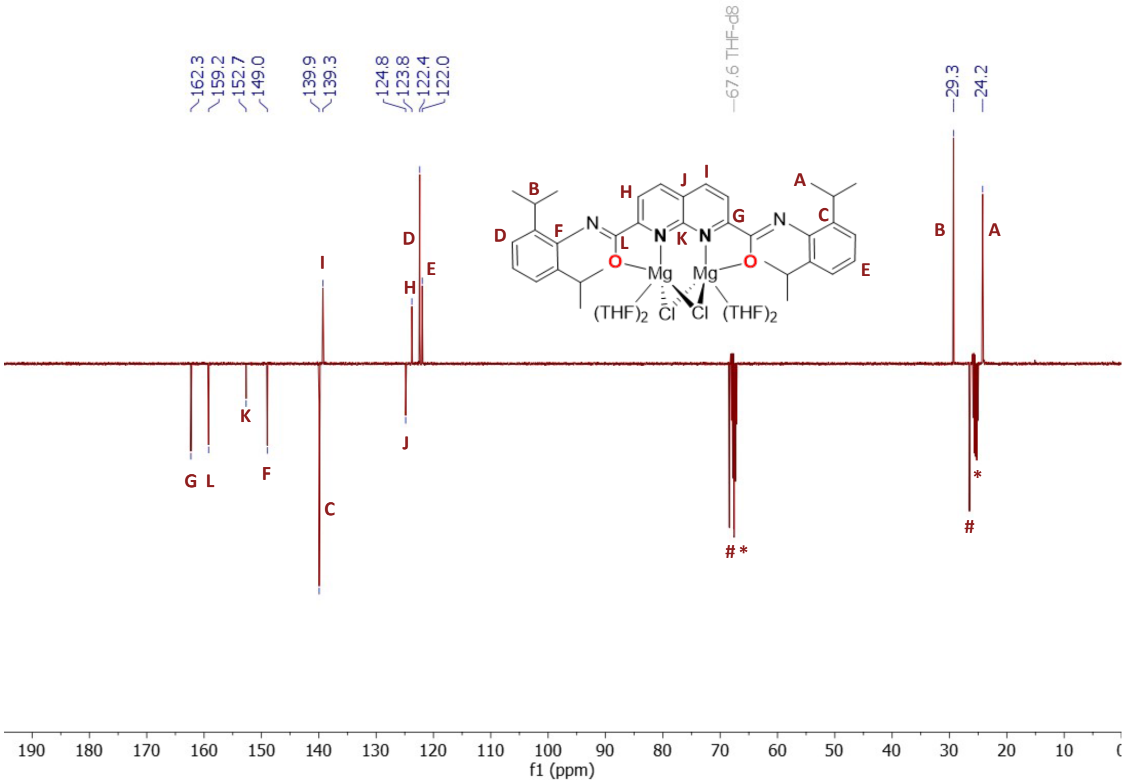

Figure S32: $^{13}C\{^{1}H\}$-NMR (APT) spectrum of **dippNDC**Mg_2Cl_2·4 THF in THF-d_8 at 298 K. Resonances marked with # are attributed to (bound) THF-H_8 and resonances marked with * are attributed to THF-d_7.

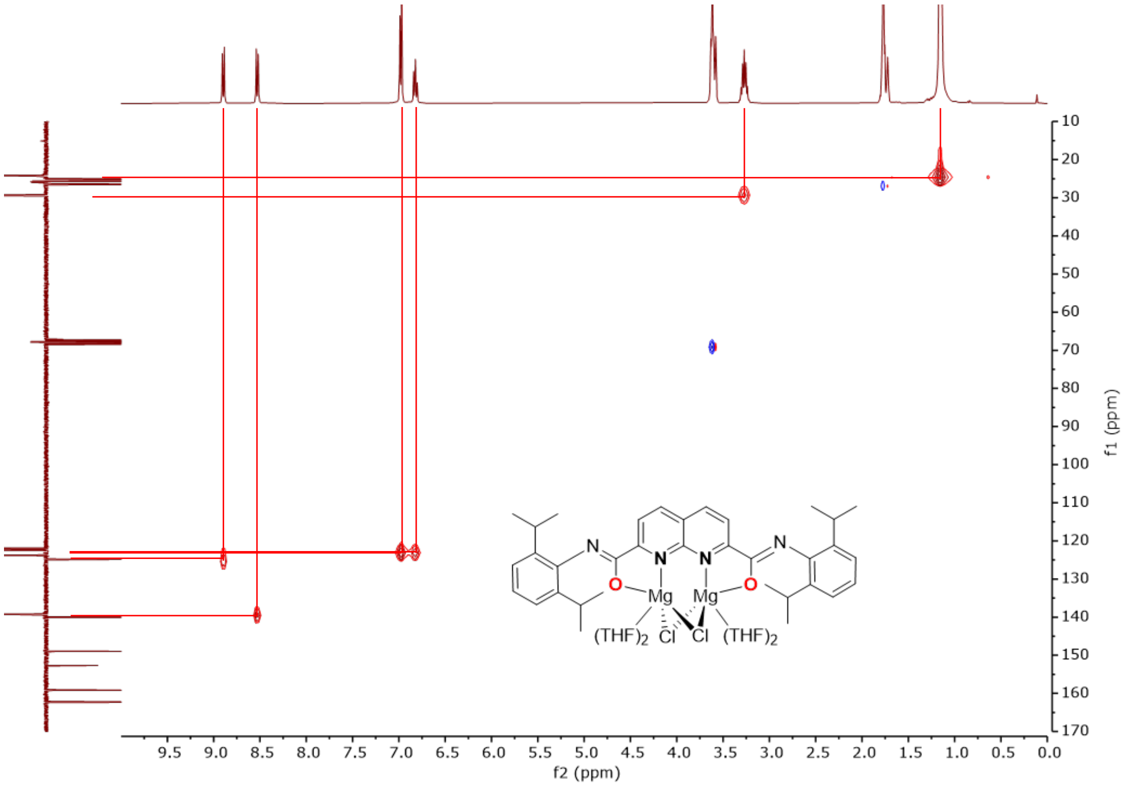

Figure S33: ^{1}H-^{13}C HSQC NMR spectrum of **dippNDC**Mg_2Cl_2·4 THF in THF-d_8 at 298 K.

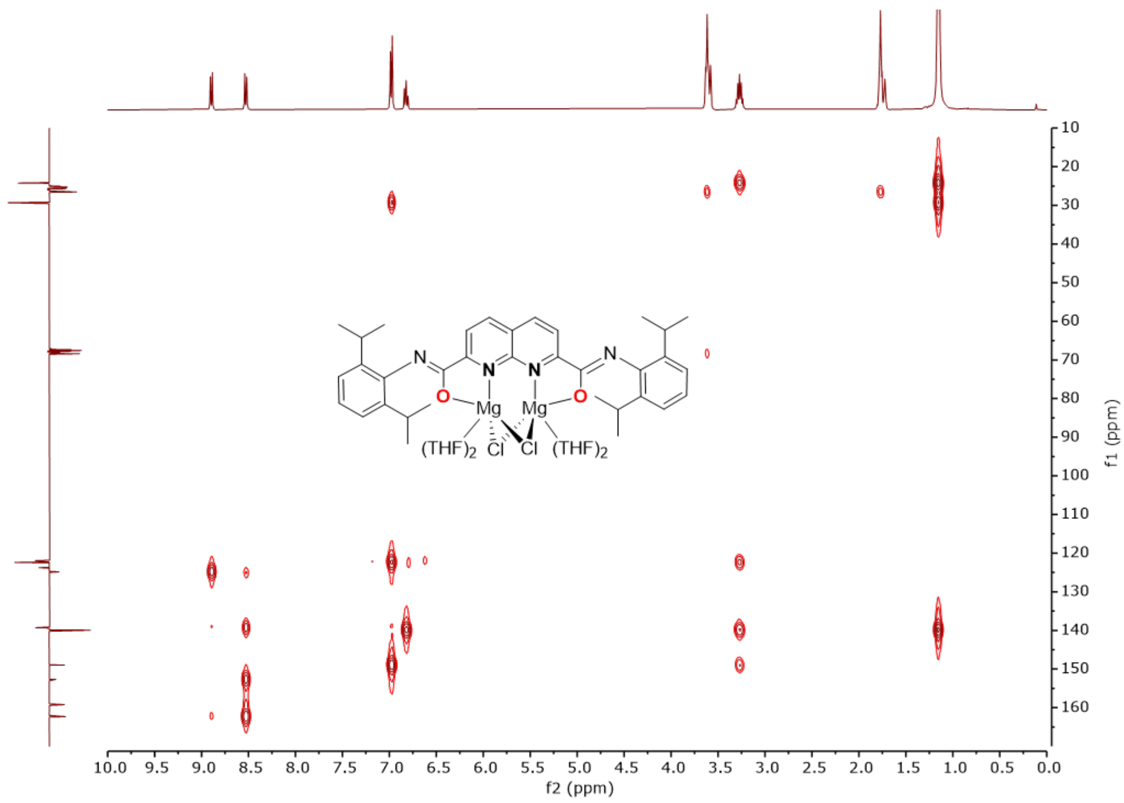

Figure S34: ^{1}H-^{13}C HMBC NMR spectrum of **^{dipp}NDC**Mg_2Cl_2·4 THF in THF-d_8 at 298 K.

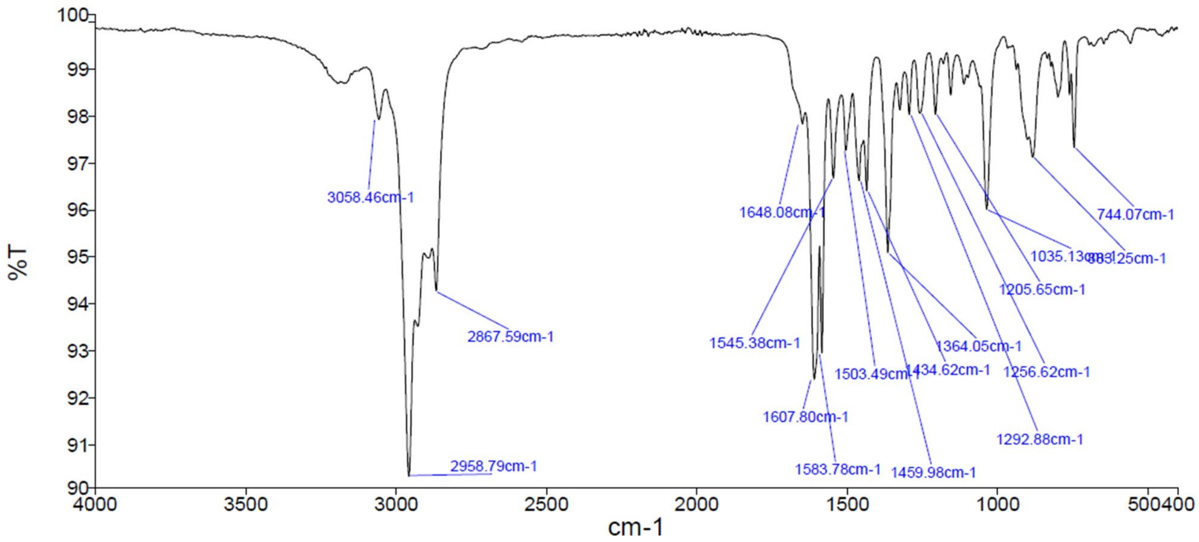

Figure S35: ATR-IR spectrum of **^{dipp}NDC**Mg_2Cl_2·4 THF measured as a film under N_2 flow at 298 K. The intensity of the peak at approx. 3200-3300 cm^{-1} increases over time, most likely due to hydrolysis of the complex.

1.8 Alternate Synthesis of ^{dipp}NDCMg$_2$Cl$_2$·4 THF:

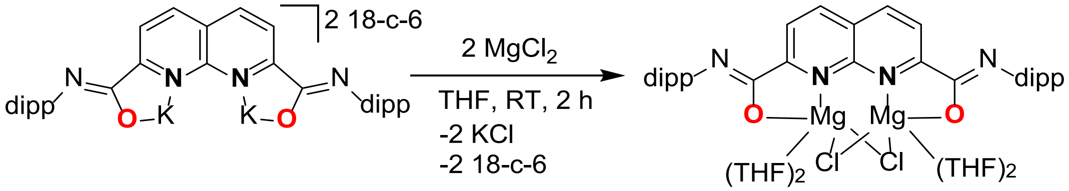

A solution of ^{dipp}**NDC**K_2·2(18-c-6) (21.4 mg, 18.7 µmol, 2.0 equiv) in THF (1.5 mL) was added dropwise to a vigorously stirred suspension of $MgCl_2$ (3.6 mg, 37.4 µmol, 2.0 equiv) in THF (1.5 mL) at ambient temperature, yielding a darker yellow mixture. The mixture was stirred for 2 h, over which the mixture turned progressively orange and cloudy. Subsequently the mixture was filtered, and the solvent was evaporated *in vacuo* to 19.4 mg of orange solid.

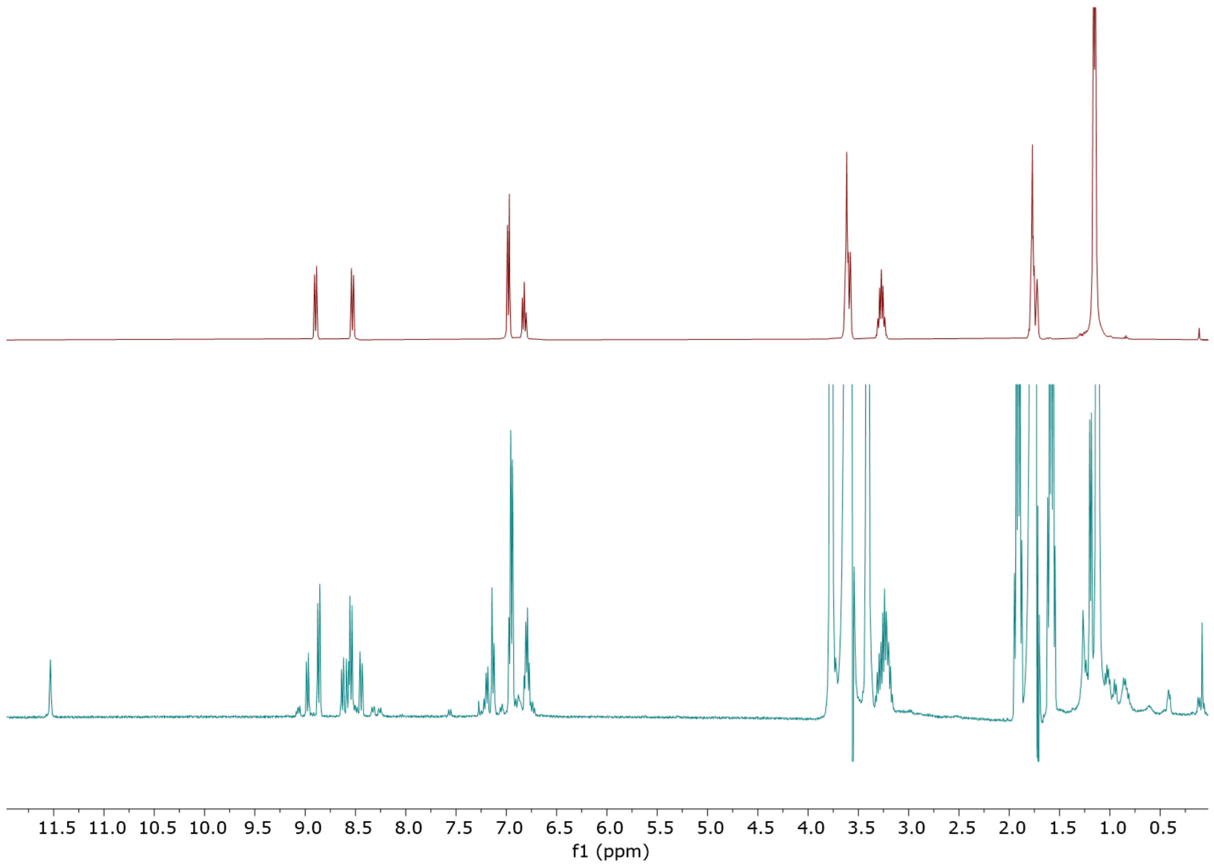

Figure S36: Stacked ^{1}H-NMR spectra of (top) ^{dipp}**NDC**Mg$_2$Cl$_2$·4 THF in THF-d_8 out of the Grignard route and (bottom) the reaction mixture in THF-H_8 containing ^{dipp}**NDC**Mg$_2$Cl$_2$·4 THF as a major species, synthesised out of the $MgCl_2$ route described in this section. Spectra recorded at 298 K.

1.9 Synthesis of ^{dipp}DAMNMg$_2$Cl$_2$·2 THF:

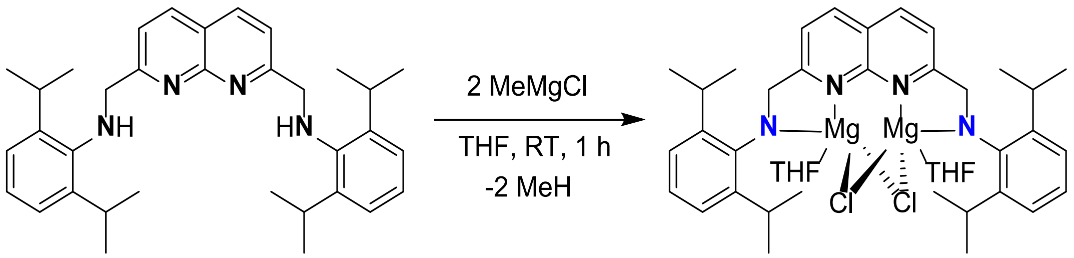

A solution of ^{dipp}**DAMN** (58.3 mg, 114.6 µmol, 1.0 equiv) in THF (10 mL) was added dropwise to a vigorously stirred solution of MeMgCl (76.4 µL, 229.2 µmol, 2.0 equiv, 3.00 M solution in THF) in THF (10 mL) at ambient temperature. The resulting dark-orange suspension was stirred for 1 h and subsequently the solvent was removed under vacuum. The solids were washed with pentane (4 x 3 mL) and dried *in vacuo* to yield ^{dipp}**DAMN**Mg$_2$Cl$_2$·2 THF as a bright orange solid (88.0 mg, 114.2 µmol, quantitative yield). Single crystals suitable for analysis by X-ray diffraction were grown by storing a saturated THF/pentane solution at −40 °C.

^{1}H NMR (400 MHz, C_6D_6, 298 K): δ 7.30 (d, $^3J_{H,H}$ = 7.4 Hz, 4H), 7.22 (t, $^3J_{H,H}$ = 7.4 Hz, 2H), 7.00 (d, $^3J_{H,H}$ = 8.3 Hz, 2H), 6.61 (d, $^3J_{H,H}$ = 8.3 Hz, 2H), 5.08 (s, 4H), 4.12 (sept, $^3J_{H,H}$ = 6.8 Hz, 2H), 3.65 (br, 8H), 1.42 (d, $^3J_{H,H}$ = 6.8 Hz, 24H), 1.25 (br, 8H).

^{13}C{^{1}H}-NMR (APT, 101 MHz, C_6D_6, 298 K): δ 170.7, 155.1, 153.6, 147.9, 136.9, 123.5, 122.3, 121.3, 121.0, 68.7*, 63.9, 27.9, 25.4*.

*Indirectly observed

IR-ATR (cm^{-1}): 3062 (w), 3034 (w), 2961 (s), 2927 (m), 2868 (m), 1608 (m), 1565 (m), 1508 (w), 1460 (m), 1456 (m), 1383 (m), 1362 (m), 1254 (m), 1102 (m), 1054 (m), 800 (m), 758 (m), 679 (m).

Elemental Analysis: The reactive nature of the compound prevented obtaining a satisfactory elemental analysis.

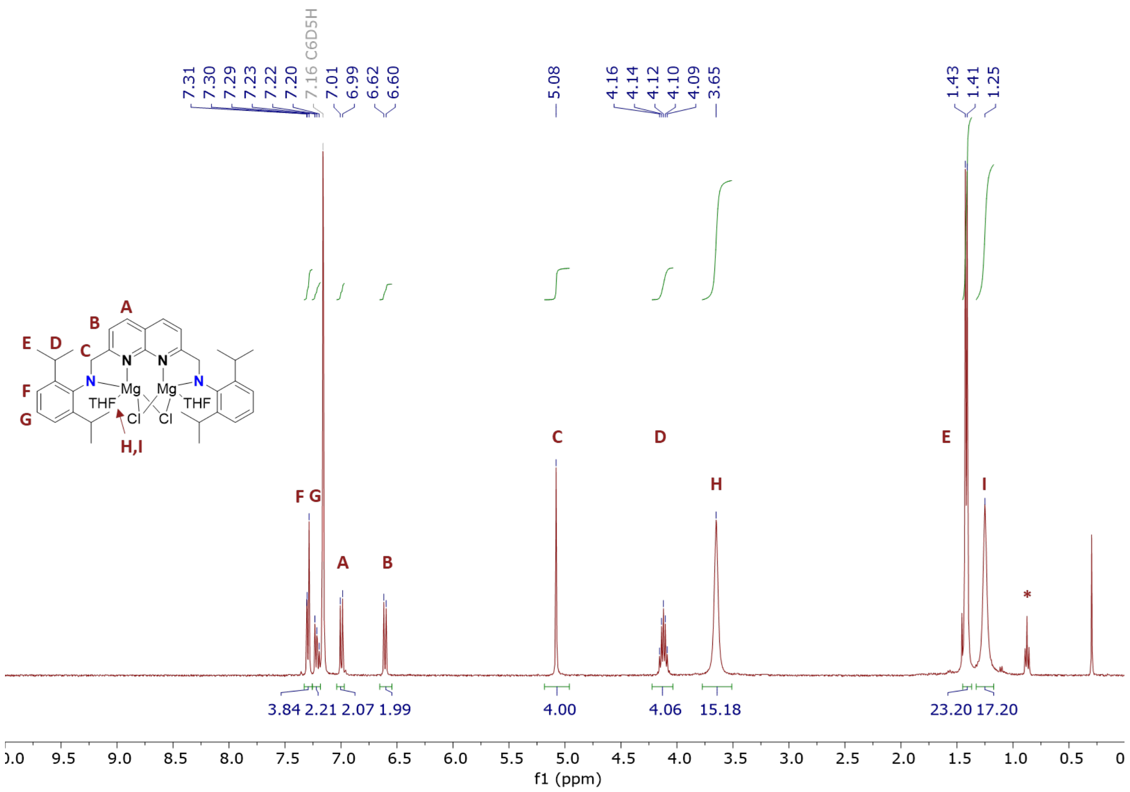

Figure S37: ^{1}H-NMR spectrum of **^{dipp}DAMN**Mg_2Cl_2·2 THF, in C_6D_6 at 298 K. Resonance indicated with an asterisk is attributed to residual hexane.

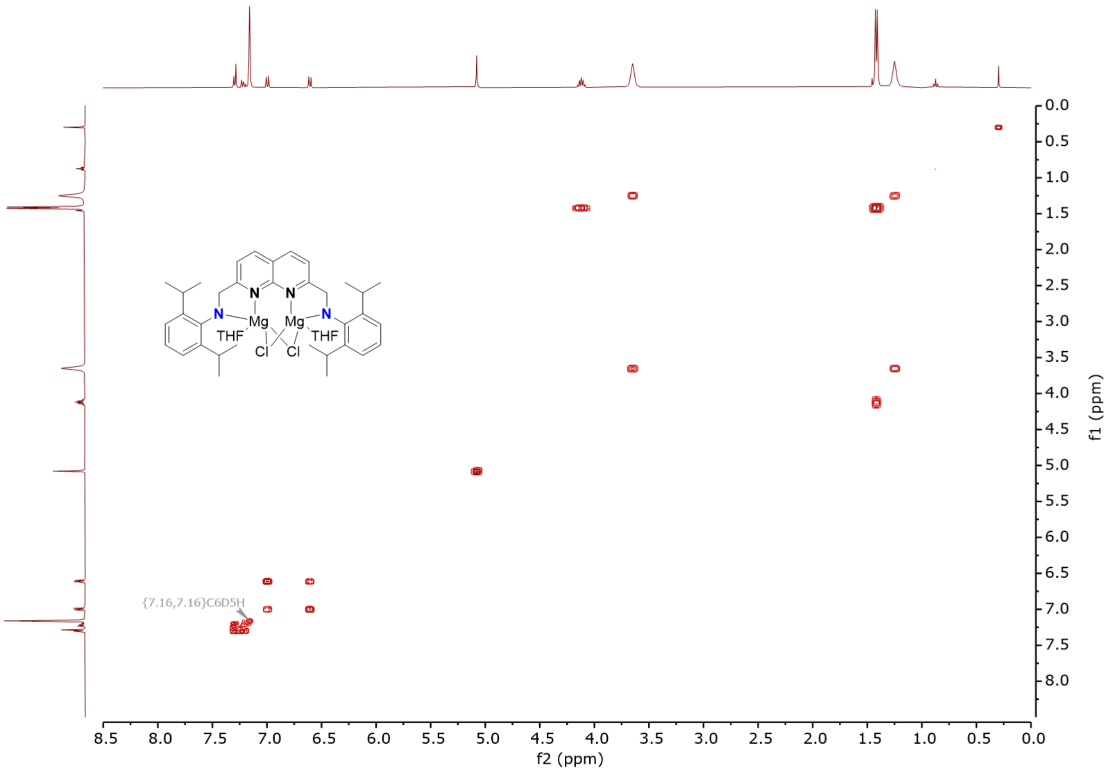

Figure S38: ^{1}H COSY NMR spectrum of **^{dipp}DAMN**Mg_2Cl_2·2 THF, in C_6D_6 at 298 K.

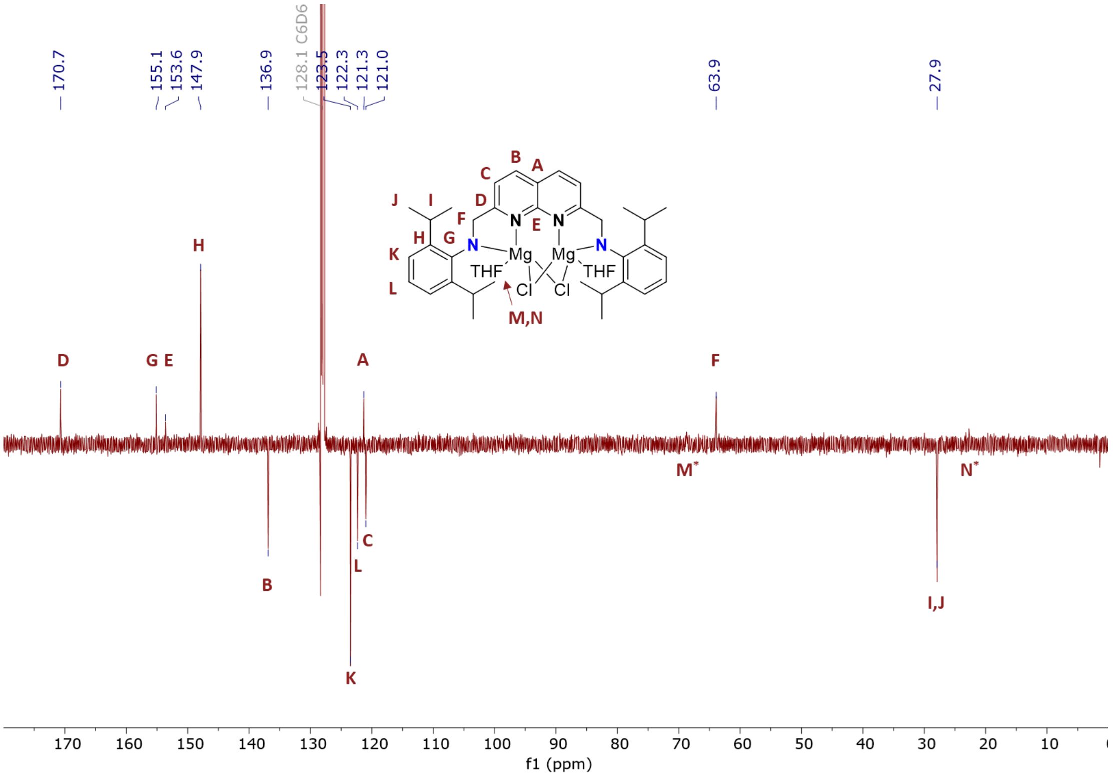

Figure S39: $^{13}C\{^{1}H\}$-NMR (APT) spectrum of **^{dipp}DAMN**Mg_2Cl_2·2 THF, in C_6D_6 at 298 K. The two resonances attributed to the bound THF ligands are not observed in this spectrum but are indirectly observed through ^{1}H-^{13}C correlation in the ASAP-HMQC spectrum below.

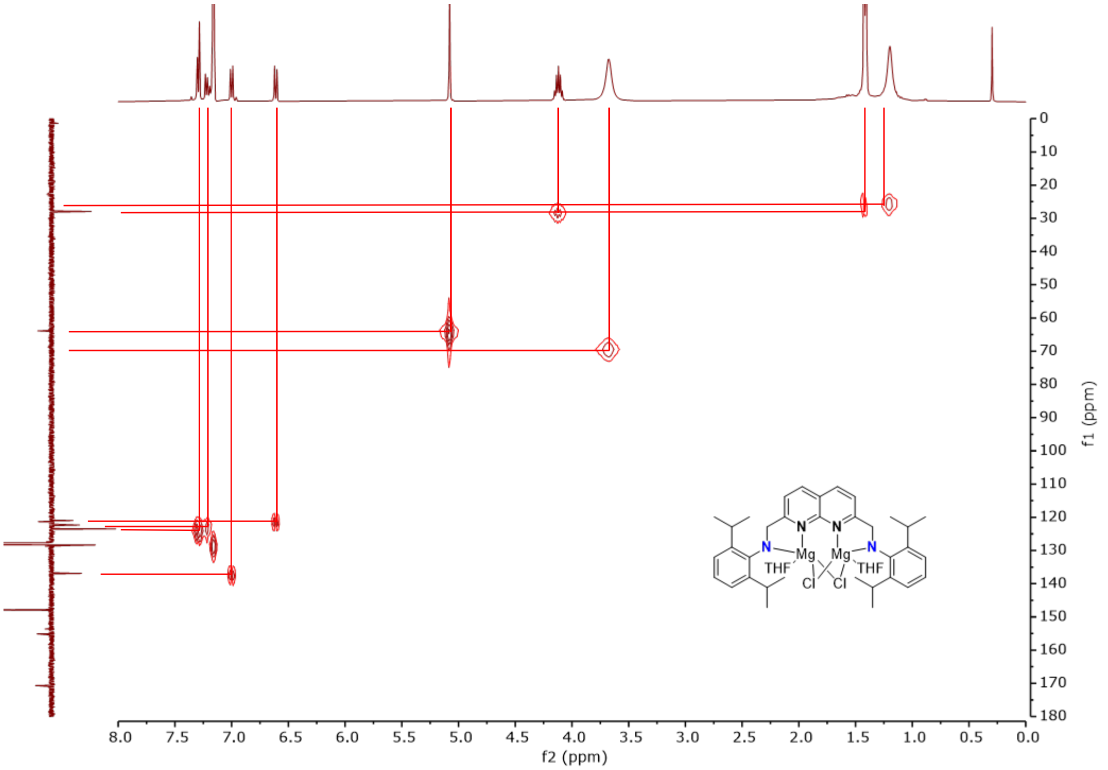

Figure S40: ^{1}H-^{13}C ASAP-HMQC NMR spectrum of **^{dipp}DAMN**Mg_2Cl_2·2 THF, in C_6D_6 at 298 K.

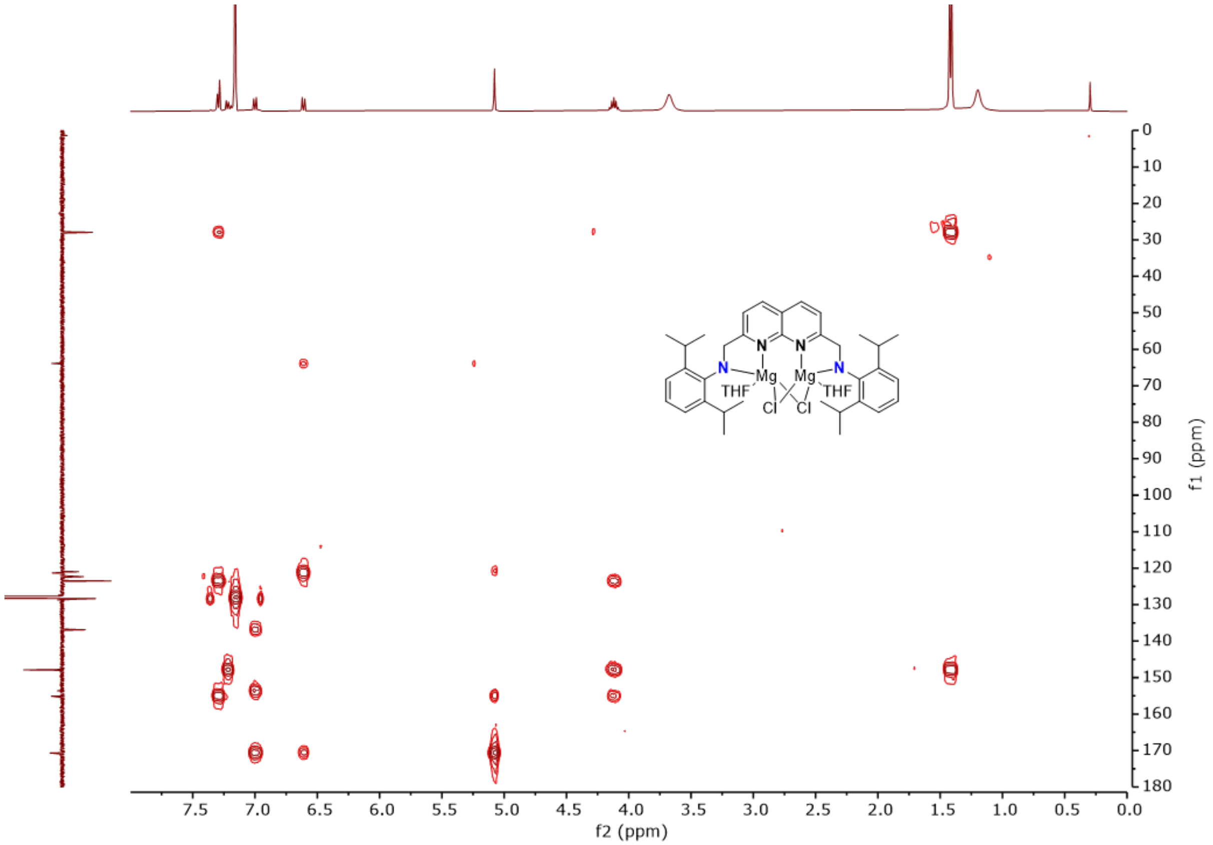

Figure S41: ^{1}H-^{13}C HMBC NMR spectrum of **^{dipp}DAMN**Mg_2Cl_2·2 THF, in C_6D_6 at 298 K.

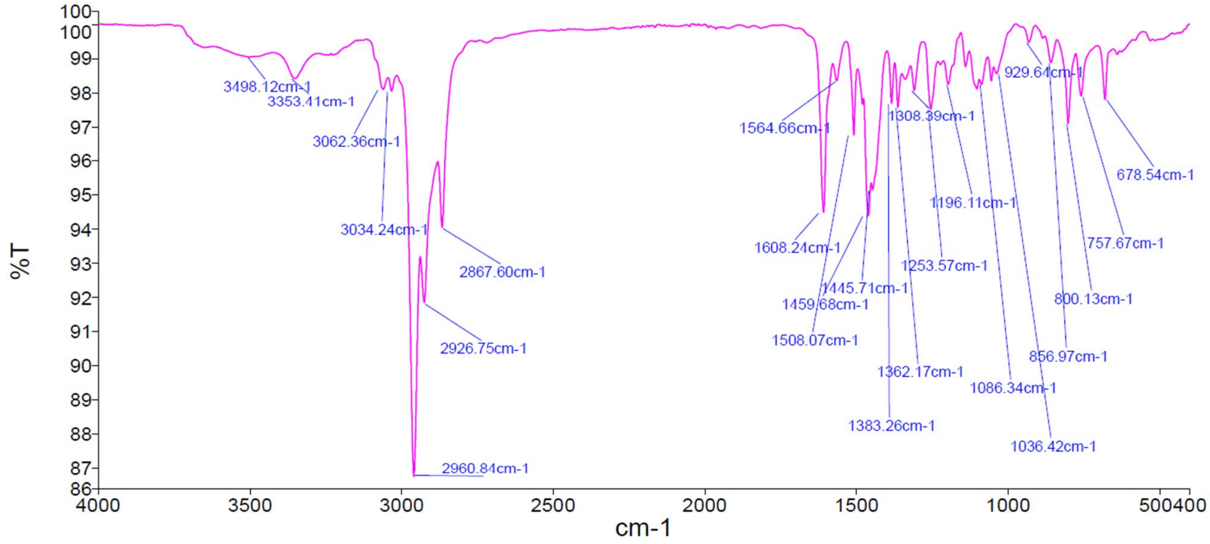

Figure S42: ATR-IR spectrum of **^{dipp}DAMN**Mg_2Cl_2·2 THF measured as a film under N_2 flow at 25 °C. Peaks above 3100 cm^{-1} increase over time due to hydrolysis.

1.10 Synthesis of ^{dipp}DAMNMg$_2$(*n*-Bu)$_2$·2 THF:

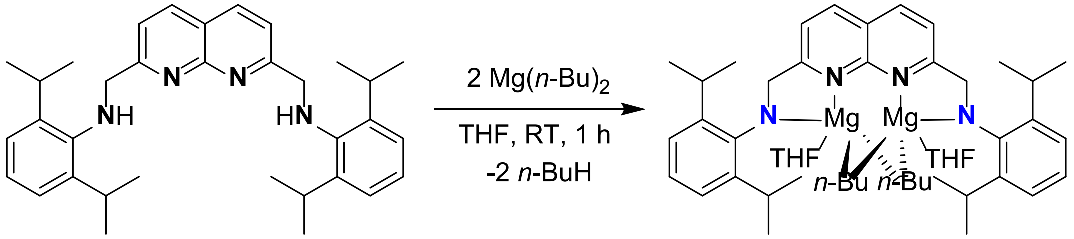

A solution of **^{dipp}DAMN** (75.0 mg, 147.5 µmol, 1.0 equiv) in THF (4 mL) was added dropwise to a vigorously stirred solution of Mg(*n*-Bu)$_2$ (842 µL, 295 µmol, 2.0 equiv, 0.5 M solution in heptane) in THF (4 mL) at ambient temperature. The resulting black-orange mixture was stirred for 1 h and subsequently the solvent was evaporated under reduced pressure to yield the title compound as a brown-orange solid (119.5 mg, 146.8 µmol, quantitative yield).

^{1}H-NMR (400 MHz, C_6D_6, 298 K): δ 7.33 ("d", 4H), 7.25 (dd, $^3J_{H,H}$ = 8.8, 6.9 Hz, 2H), 7.09 (d, $^3J_{H,H}$ = 8.4 Hz, 2H), 6.61 (d, $^3J_{H,H}$ = 8.4 Hz, 2H), 4.95 (s, 4H), 4.10 (sept, $^3J_{H,H}$ = 6.9 Hz, 4H), 3.37 (br, 8H), 1.49 (m, 4H*), 1.48 (d, $^3J_{H,H}$ = 6.9 Hz, 24H*), 1.31 (m, 4H), 1.18 (br, 8H), 0.90 (t, $^3J_{H,H}$ = 7.1 Hz, 6H), 0.01 (m, 4H).

*Overlapping resonances

^{13}C{^{1}H}-NMR (APT, 101 MHz, C_6D_6, 298 K): δ 170.3, 154.7, 151.7, 147.7, 136.2, 123.8, 122.6, 120.8, 119.4, 63.9, 31.3, 30.9, 27.7, 26.0, 25.3**, 14.0, 10.1.

**Indirectly observed in the ASAP-HMQC spectrum

ATR-IR (cm^{-1}): 3048 (w), 2957 (s), 2926 (m), 2865 (m), 2721 (w), 2673 (w), 2279 (w), 1602 (m), 1512 (m), 1458 (m), 1431 (m), 1420 (m), 1359 (m), 1311 (m), 1251 (m), 1199 (m), 1140(w), 1103 (m), 1056 (m), 908 (m), 841 (m), 799 (m), 770 (m), 498 (m).

Elemental Analysis: The reactive nature of the compound prevented obtaining a satisfactory elemental analysis.

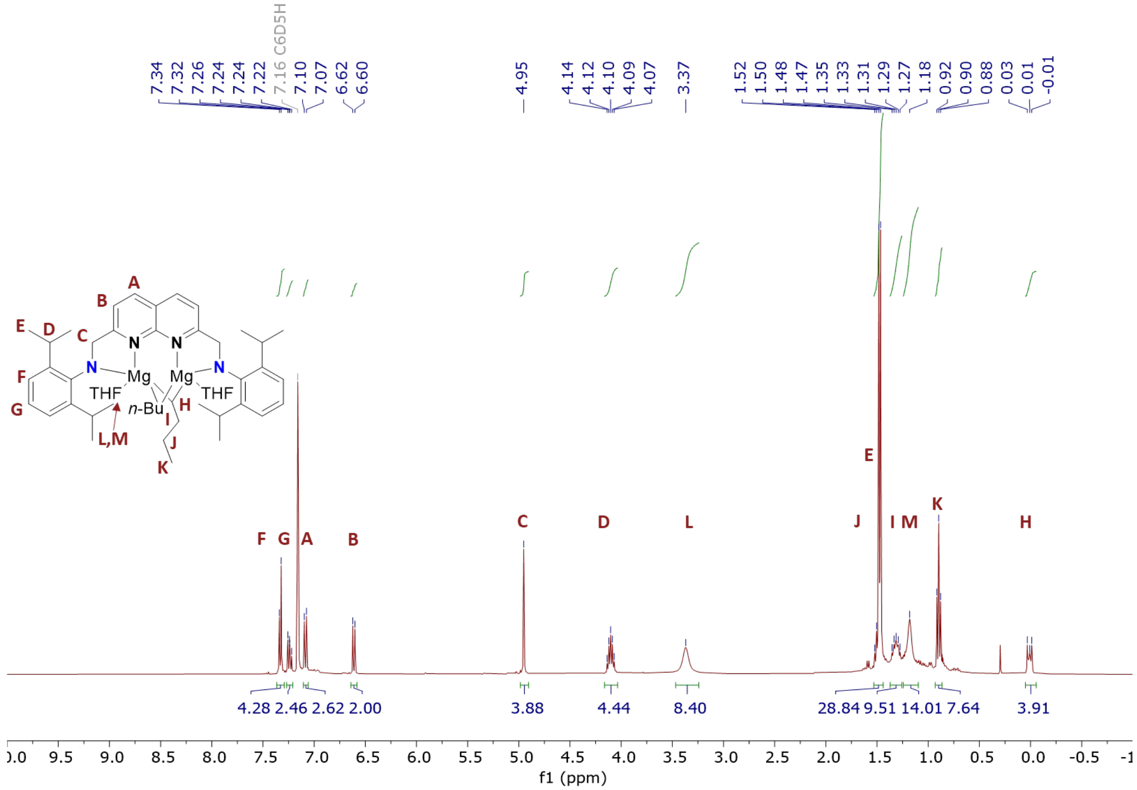

Figure S43: ^{1}H-NMR spectrum of $^{\mathbf{dipp}}$**DAMN**$Mg_2(n\text{-}Bu)_2 \cdot 2$ THF, in C_6D_6 at 298 K.

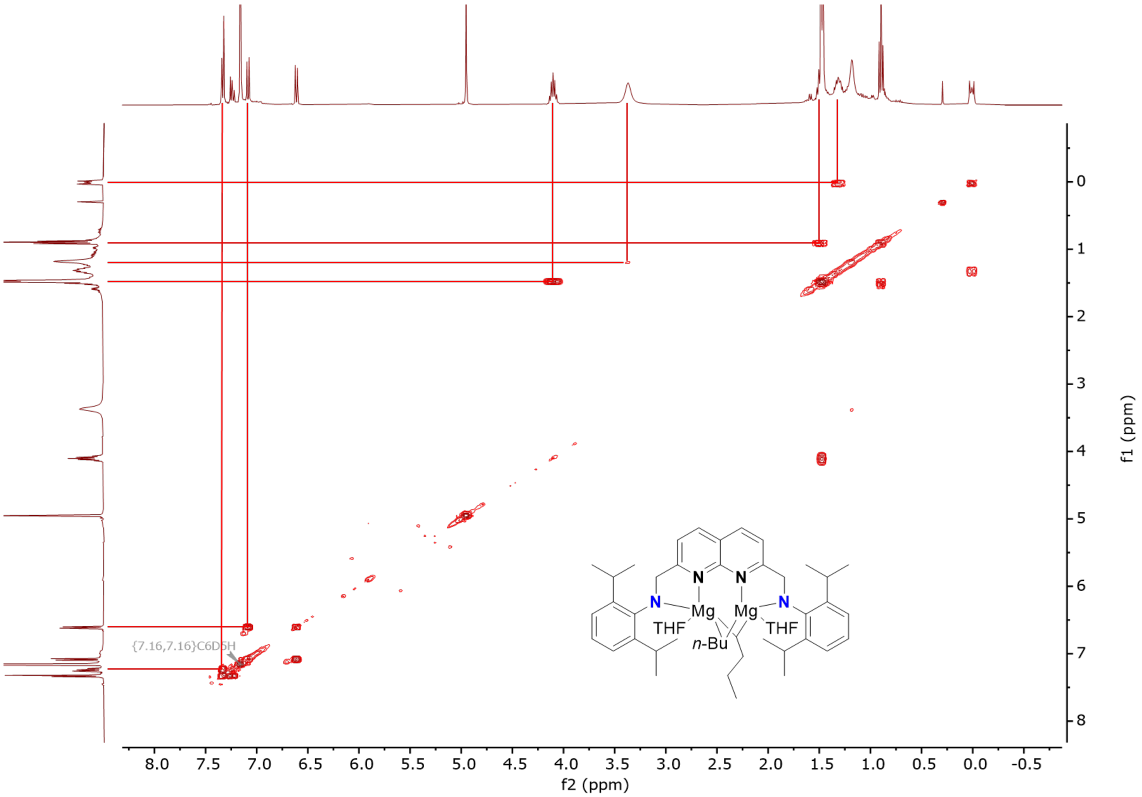

Figure S44: ^{1}H COSY NMR spectrum of $^{\mathbf{dipp}}$**DAMN**$Mg_2(n\text{-}Bu)_2 \cdot 2$ THF, in C_6D_6 at 298 K.

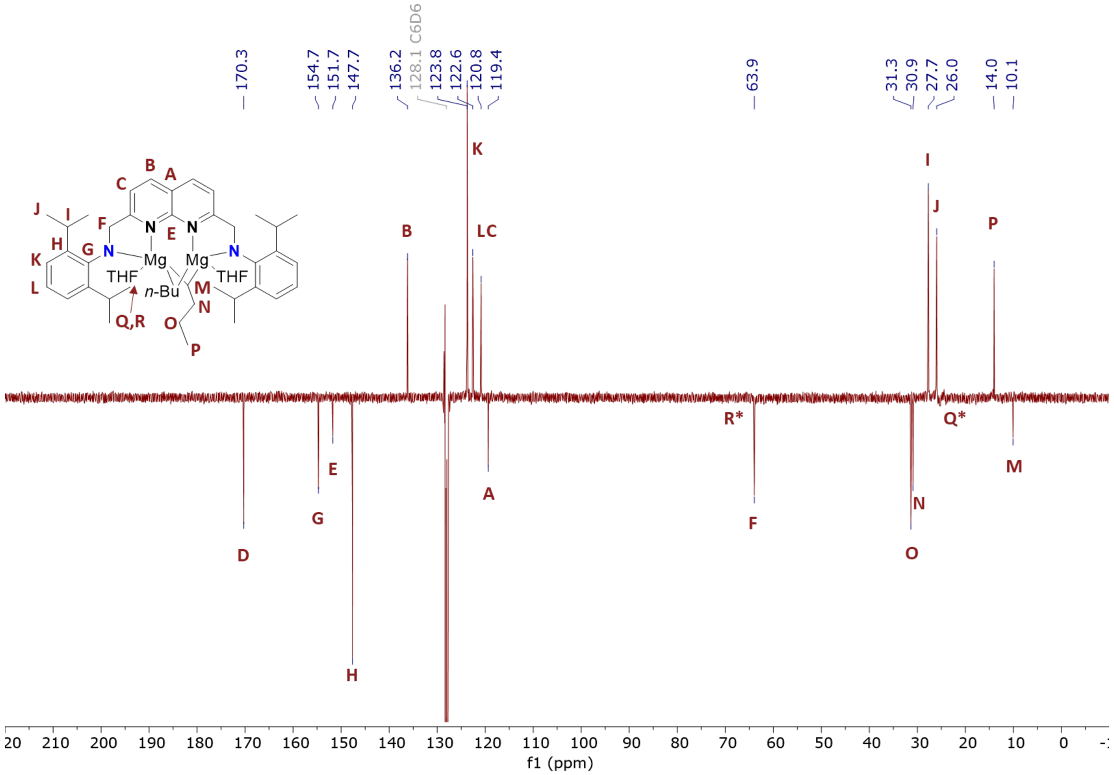

Figure S45: $^{13}C\{^1H\}$-NMR (APT) spectrum of **^{dipp}DAMN**Mg_2(*n*-Bu)$_2$·2 THF, in C_6D_6 at 298 K. The two resonances attributed to the bound THF ligands are not observed in this spectrum but one of them (Q) is indirectly observed through ^1H-^{13}C correlation in the ASAP-HMQC spectrum below.

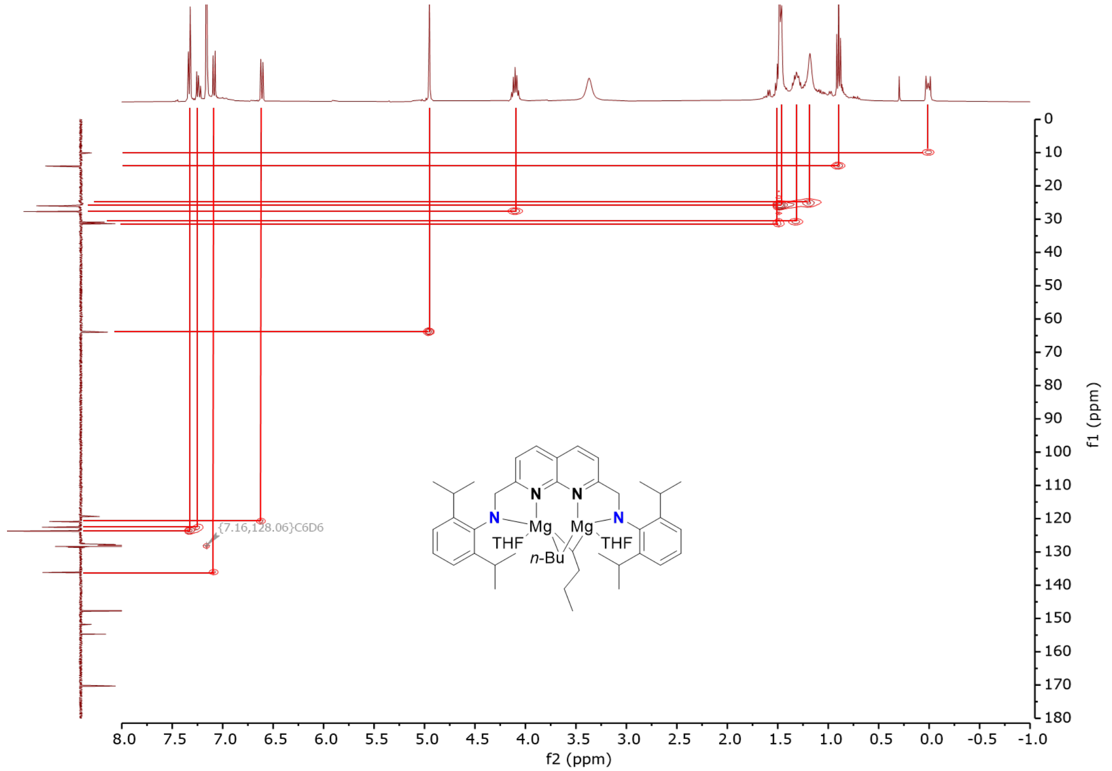

Figure S46: ^1H-^{13}C ASAP-HMQC NMR spectrum of **^{dipp}DAMN**Mg_2(*n*-Bu)$_2$·2 THF, in C_6D_6 at 298 K.

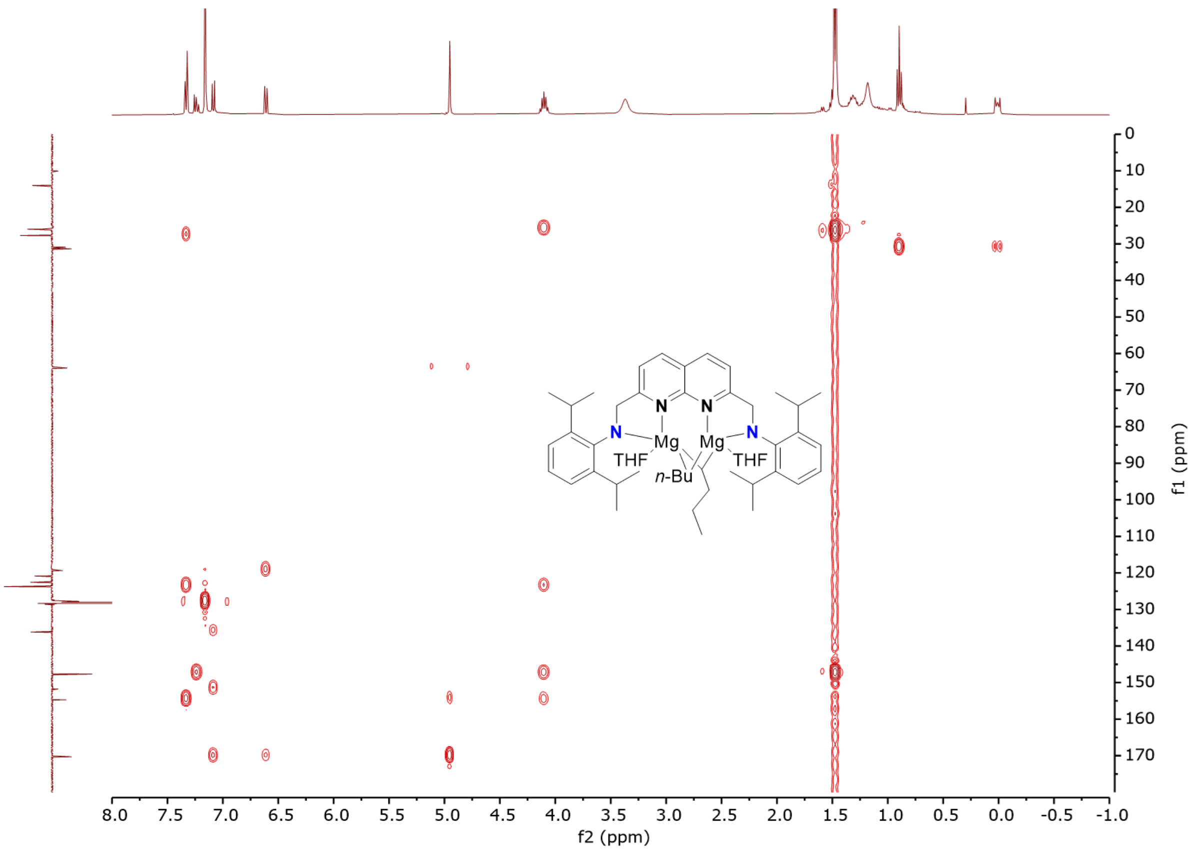

Figure S47: ^{1}H-^{13}C HMBC NMR spectrum of **^{dipp}DAMN**$Mg_2(n\text{-}Bu)_2 \cdot 2$ THF, in C_6D_6 at 298 K.

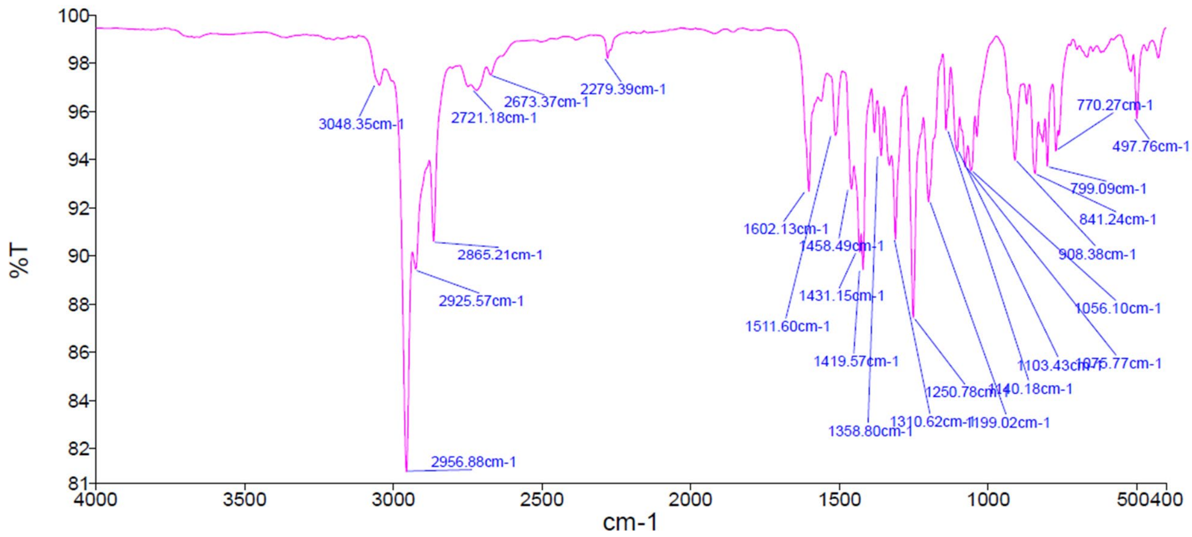

Figure S48: ATR-IR spectrum of **^{dipp}DAMN**$Mg_2(n\text{-}Bu)_2 \cdot 2$ THF measured as a film under N_2 flow at 298 K.

1.11 Reacting ^{dipp}NDC with Mg(*n*-Bu)$_2$:

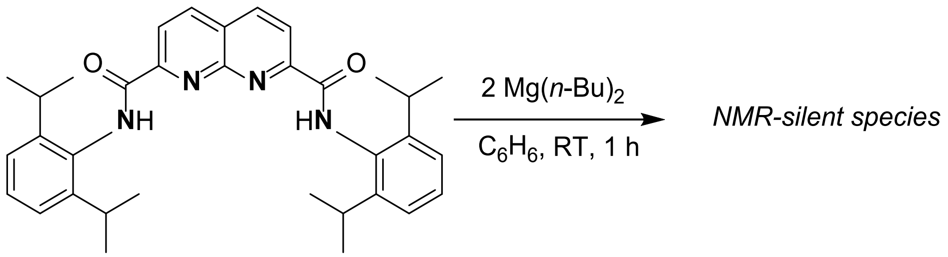

A solution of ^{dipp}**NDC** (10.0 mg, 18.6 µmol, 1.0 equiv) in benzene (1.5 mL) was added dropwise to a vigorously stirred solution of Mg(*n*-Bu)$_2$ (107 µL, 37 µmol, 2.0 equiv, 0.35 M solution in heptane) in benzene (1.5 mL) at ambient temperature. The resulting dark-green solution was stirred for 1 h and subsequently transferred out of the glovebox and quenched with water (1.5 mL). The resulting colourless solution was extracted with CH_2Cl_2 (3 x 3 mL), dried with Na_2SO_4, filtered and dried under vacuum yielding a yellow solid (5.4 mg).

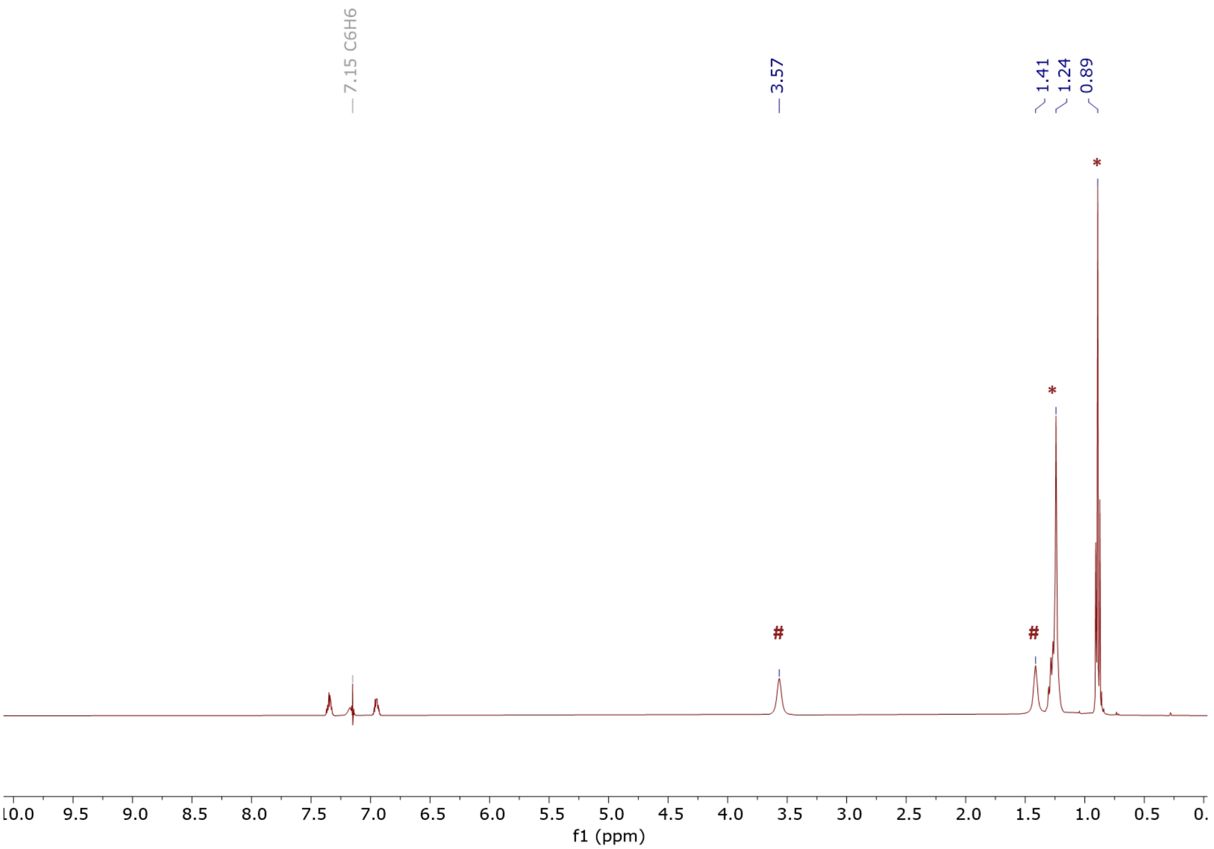

Figure S49: ^{1}H-NMR spectrum (PRESAT) of the reaction mixture of ^{dipp}**NDC** with 2 equiv Mg(*n*-Bu)$_2$, in C_6H_6 at 298 K. Resonances marked with a # are attributed to residual THF and resonances marked with a * are attributed to residual heptane.

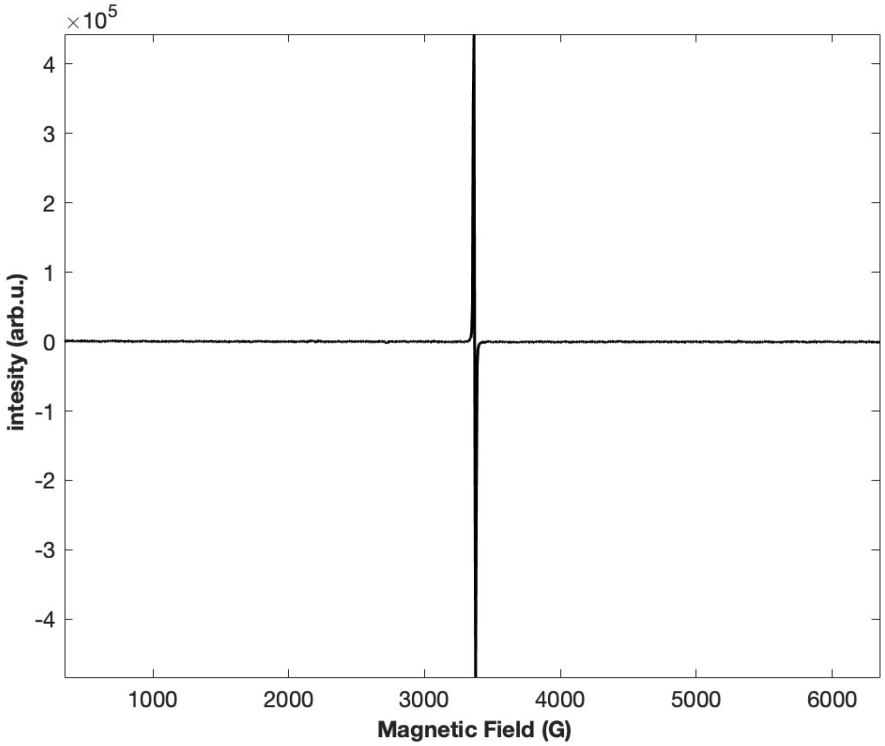

Figure S50: EPR spectrum of the reaction mixture of **^{dipp}NDC** with 2 equiv Mg(*n*-Bu)$_2$, in C_6D_6 at 298 K.

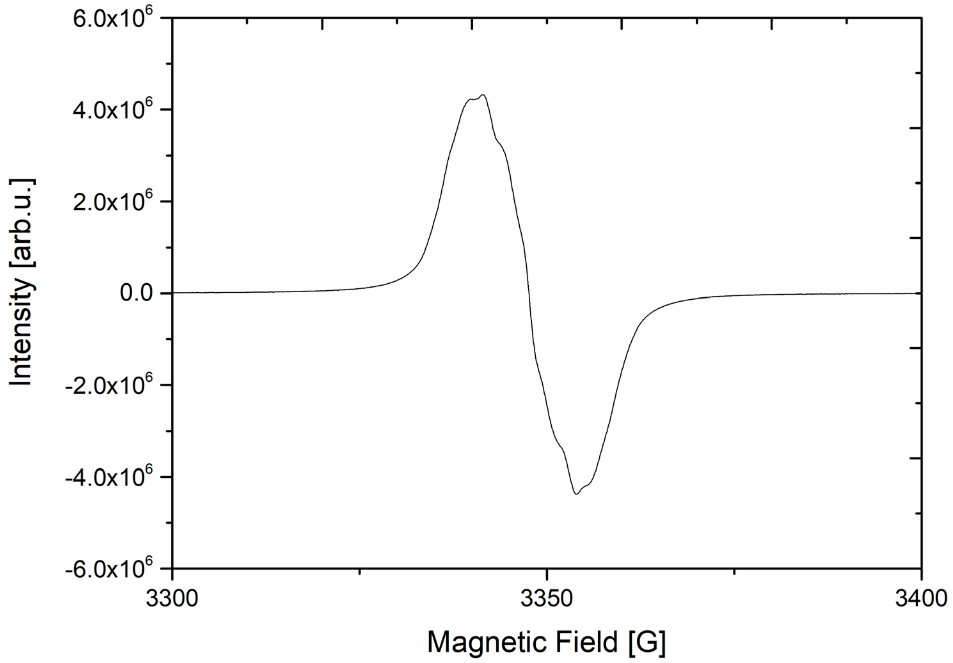

Figure S51: Zoomed-in version of the EPR spectrum depicted on Figure S50.

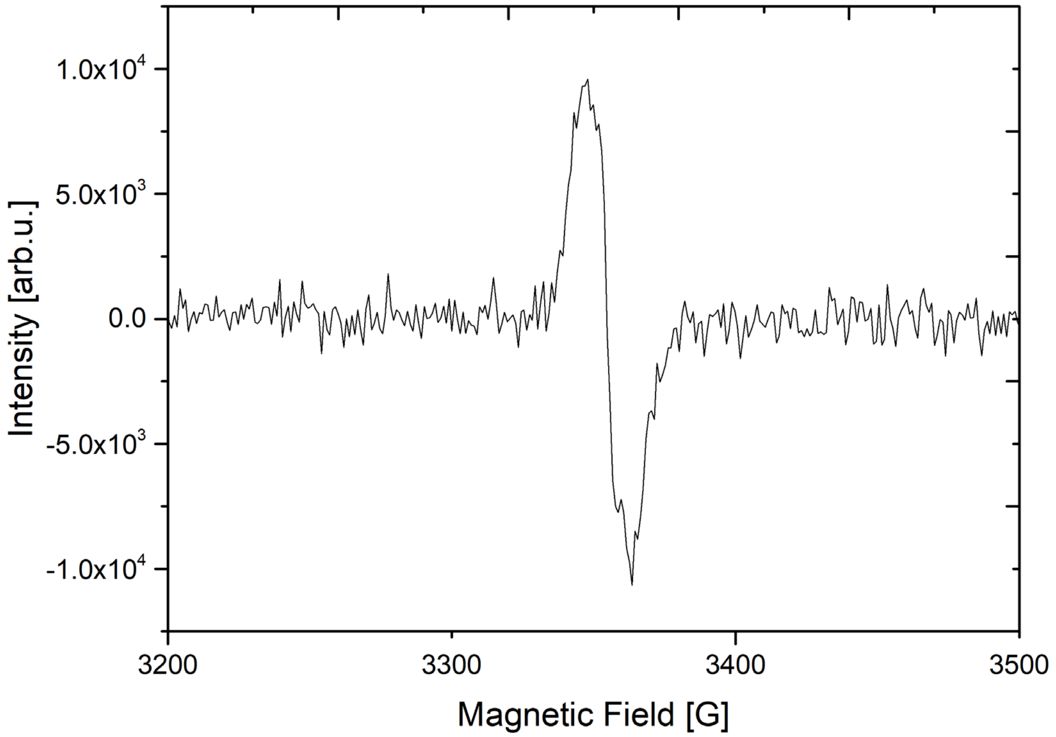

Figure S52: EPR spectrum of the reaction mixture of **^{dipp}NDC** with 2 equiv Mg(*n*-Bu)$_2$, measured under dilute conditions (0.1 mM) in C_6H_6 at 298 K.

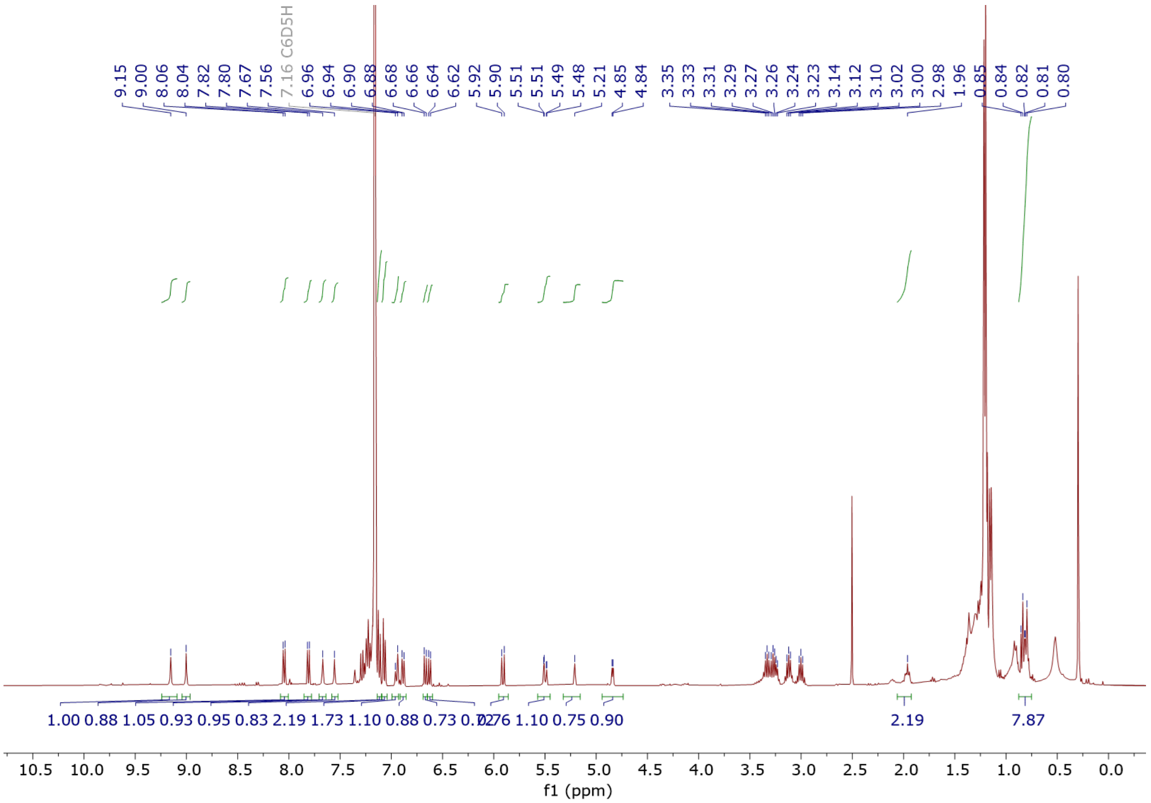

Figure S53: ^{1}H-NMR spectrum of the reaction mixture after quenching with H_2O, in C_6D_6 at 298 K.

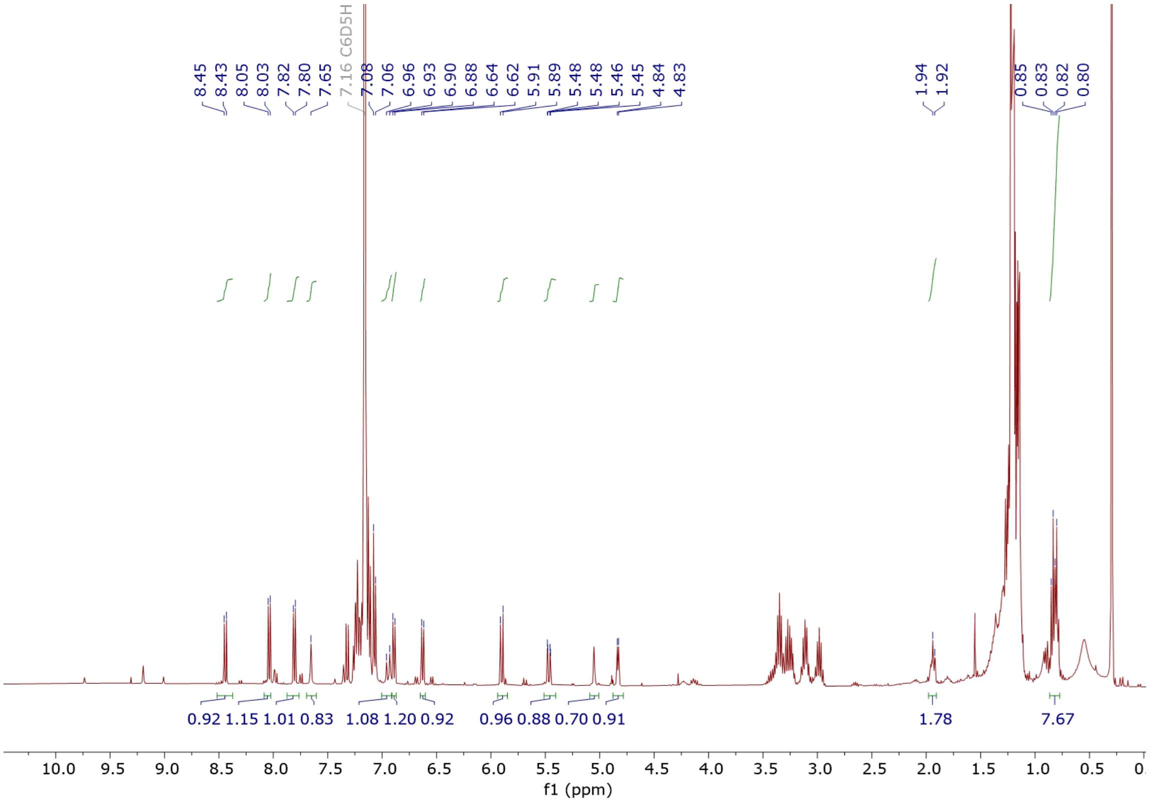

Figure S54: ^{1}H-NMR spectrum of the reaction mixture after quenching with D_2O, in C_6D_6 at 298 K.

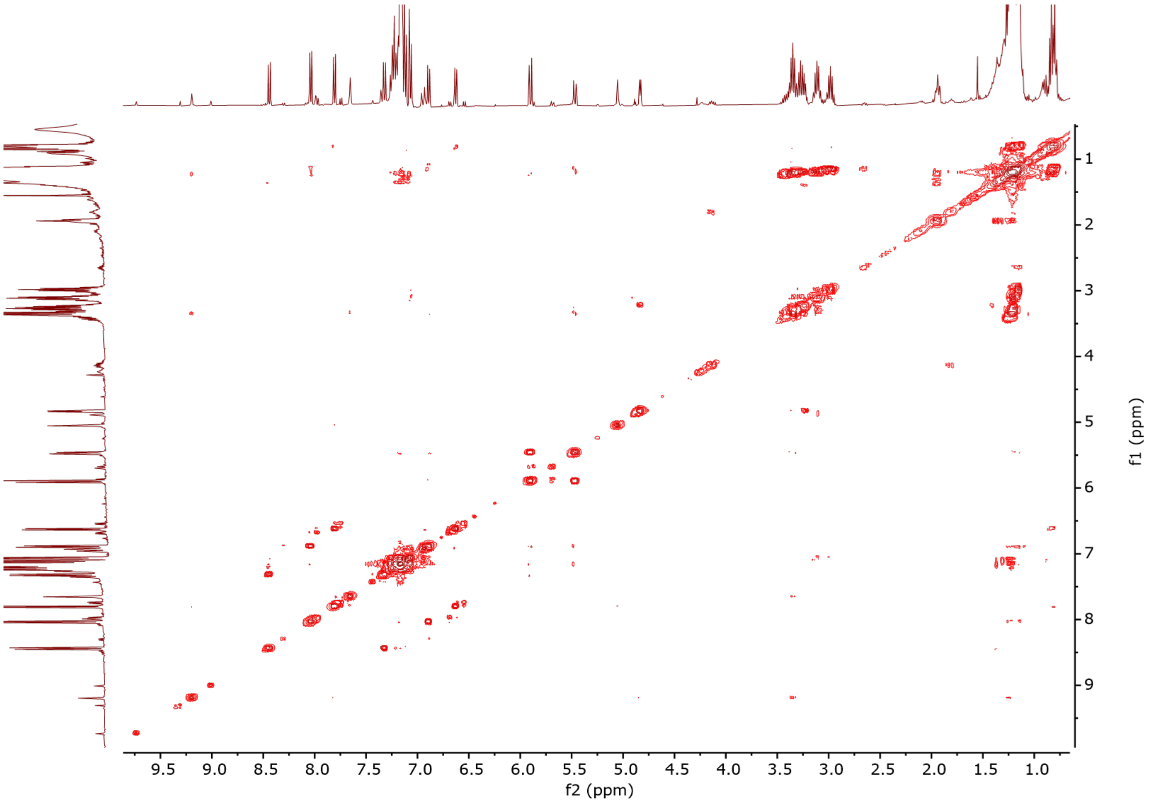

Figure S55: ^{1}H-^{1}H COSY NMR spectrum of the reaction mixture after quenching with D_2O, in C_6D_6 at 298 K.

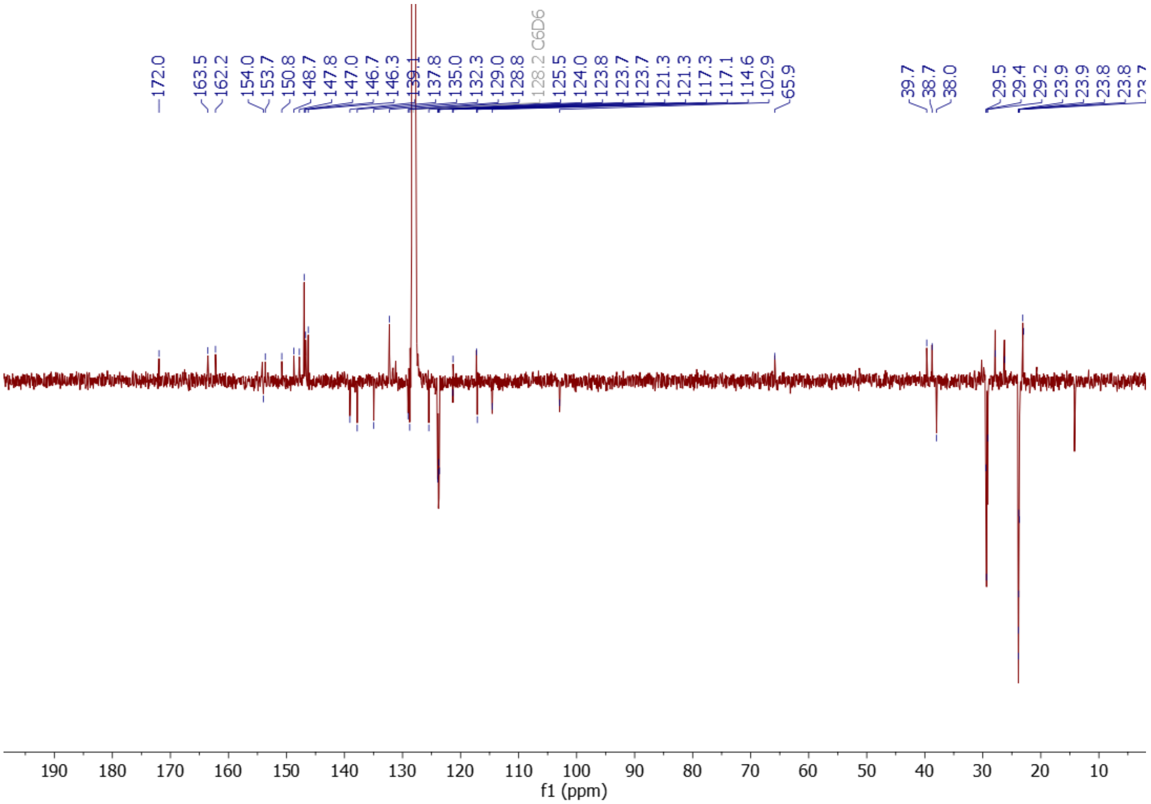

Figure S56: $^{13}C\{^{1}H\}$-NMR (APT) spectrum of the reaction mixture after quenching with D_2O, in C_6D_6 at 298 K.

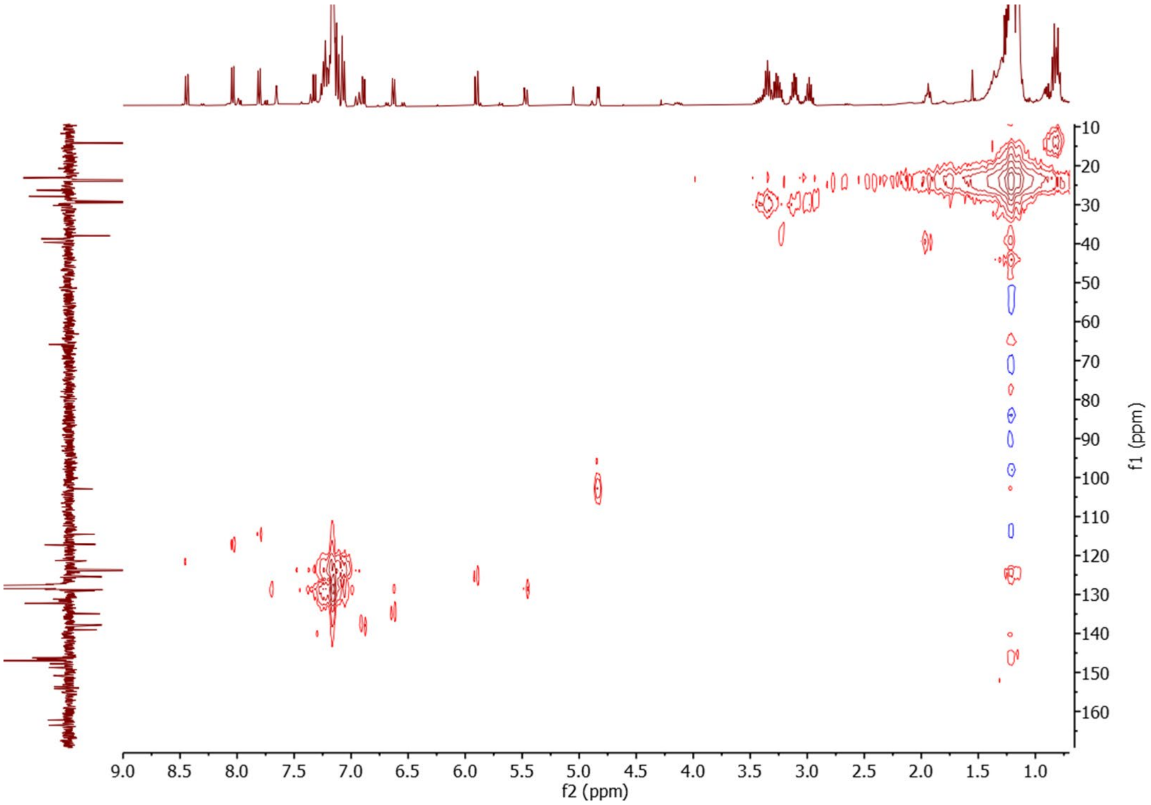

Figure S57: ^{1}H-^{13}C ASAP-HMQC NMR spectrum of the reaction mixture after quenching with D_2O, in C_6D_6 at 298 K.

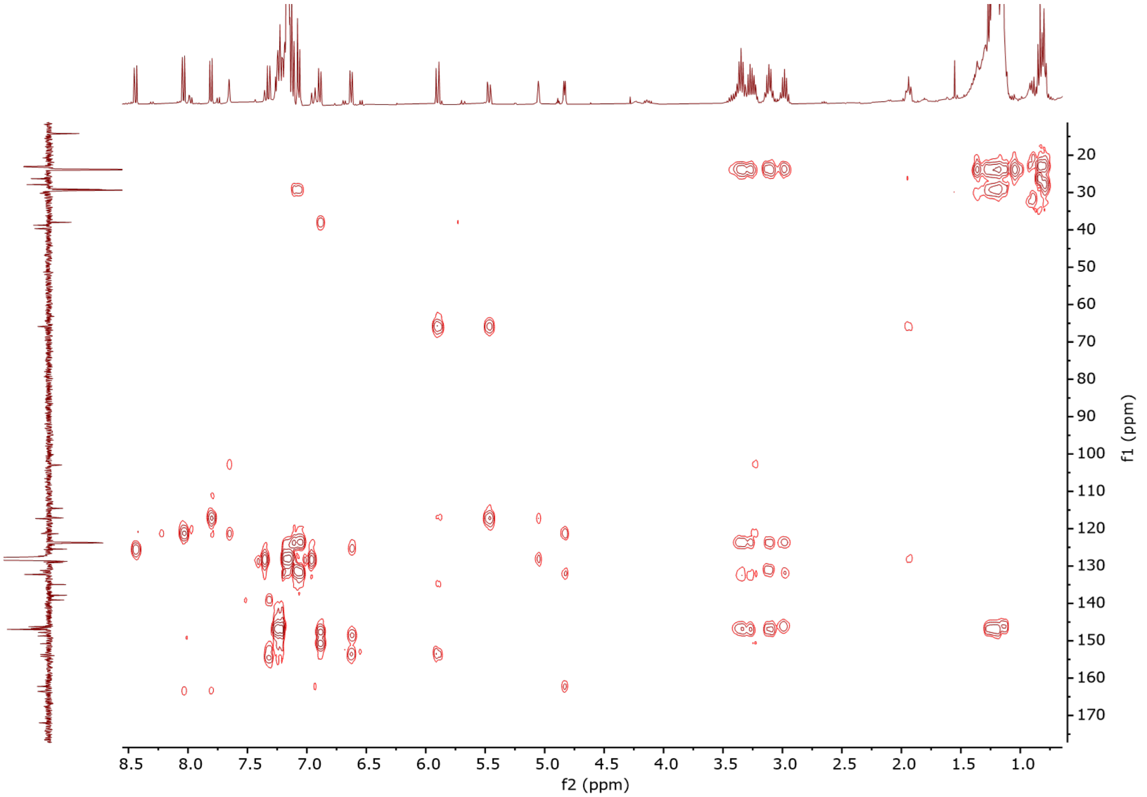

Figure S58: ^{1}H-^{13}C ASAP-HMBC NMR spectrum of the reaction mixture after quenching with D_2O, in C_6D_6 at 298 K.

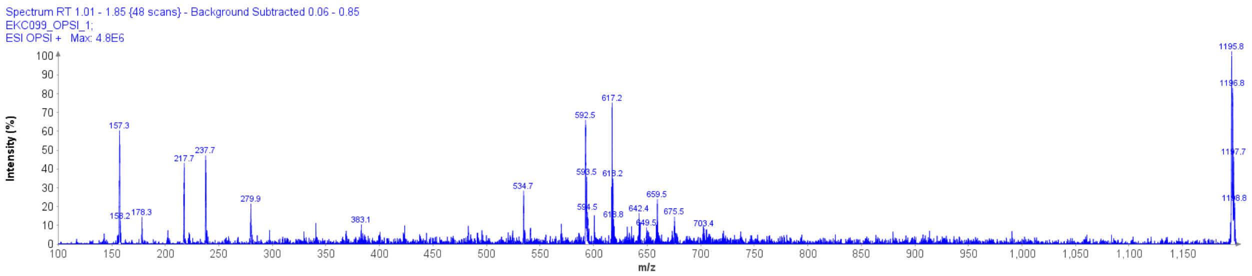

Figure S59: ESI-MS spectrum (positive mode) of the solids obtained after quenching the reaction mixture with H_2O.

1.12 Discussion of the Proposed Equilibria:

It is well established that C–C coupling processes of N-heterocyclic (expanded) pincer ligands typically lead to irreversible products. However, in several documented systems it is demonstrated that the resulting C–C bonds can be relatively weak and cleavable under mild oxidative conditions.[3] Despite many examples of dimerisation resulting in (shallow) thermodynamic sinks, examples are known where the dimerisation of small N-heterocyclic substrates is reversible.[4,5] The involvement of N-heterocyclic radicals in equilibria is not limited to substrates but has also been demonstrated for a pyridine diimine ligand bound to MgR_2 through EPR spectroscopy and isotopic labelling.[6] In this system, the equilibrium lies on the side of the diamagnetic species. It is important to recognize that in fused aromatic systems, the energetic penalty for the initial dearomatisation is significantly less than in their non-fused counterparts, due to the higher stability of the formed radical. This principle is exemplified in the greater reduction potential required for benzene compared to naphthalene.[7] Extending this rationale, reduction of a naphthyridine motif should

be even more facile than a pyridine motif, due to the increased stability of the naphthyridine radical. Applying these arguments to our system, the large, conjugated structure of **^{dipp}NDC** renders it likely that a comparatively (to non-fused N-heterocycles) more stabilised radical species can exist in equilibrium with its dimerised and butylated forms under the given reaction conditions. EPR-spectroscopic data are in agreement with the presence of an organic radical in the reaction mixture. Additionally, both NMR- and MS-data confirm the presence of dimerised and butylated species upon quenching the radical species with water, which support our hypothesis that radical species are most likely in equilibrium with species such as those proposed in Scheme 7 of the manuscript. If radical recombination was irreversible or no equilibrium existed, diamagnetic species would be detectable prior to quenching, which was not observed. If no such equilibria were present in the reaction mixture, it would necessitate that formation of the dimerised and butylated species observed after quenching occurs exclusively upon or after quenching of the radical species with water — a scenario we consider less plausible. The absence of NMR-active species prior to quenching also suggests that the equilibria lie on the side of the radical species.

2. Computational Methods:

2.1 General Considerations:

Calculations were performed using the Gaussian 16 rev. C.01 & C.02 software.[8,9] The Becke 1988 exchange functional (B3LYP) was used.[10,11] The 6-31G** basis set was used for the geometry optimisations.[12–16] For single point calculations the 6-311G** basis set was used.[13,17,18] Starting geometries for the optimisations were obtained from the coordinates of the crystal structures if possible, or by modification of the optimised geometry of the most similar complex. Additionally, Grimme's DFT-D3 scheme for atom-pairwise dispersion correction was used for all atoms in every calculation.[19,20] The absence of imaginary frequencies was checked to confirm that the optimised structures correspond to real local minima.

2.2 Example Input File for Geometry Optimisations:

```
#p
scf=(maxcycle=300)
opt
freq=noraman
B3LYP/6-31G**
EmpiricalDispersion=GD3BJ
nosym
int=ultrafine

"Title"

0 1 or 0 3
[Cartesian Coordinates here]
```

2.3 Example Input File for SP Calculations:

```
#p
scf=(maxcycle=300)
SP
B3LYP/6-311G**
EmpiricalDispersion=GD3BJ
nosym
int=ultrafine

"Title"

0 1 or 0 3
[Cartesian Coordinates here]
```

2.4 Calculated Energies of $^{dipp}DAMNMg_2Me_2{\cdot}2$ THF (Singlet):

Electronic energy from SP calculation: −2485.85239257 hartrees
Gibbs thermal correction: 0.91102 hartrees

2.5 Calculated Energies of ^{dipp}DAMNMg$_2$Me$_2$·2 THF (Triplet):

Electronic energy from SP calculation: −2485.78733701 hartrees
Gibbs thermal correction: 0.907047 hartrees

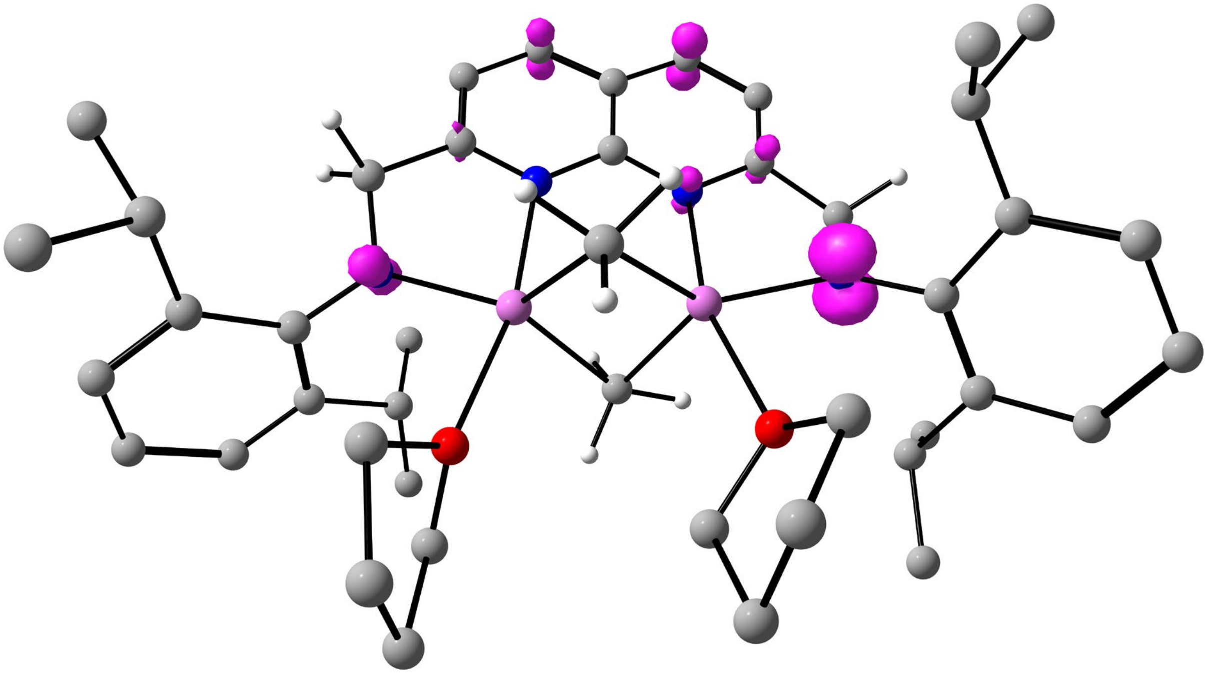

Figure S60: Residual spin density plot of ^{dipp}**DAMN**Mg$_2$Me$_2$·2 THF (Triplet).

2.6 Calculated Energies of ^{dipp}NDCMg$_2$Me$_2$·4 THF (Singlet):

Electronic energy from SP calculation: −3099.13590401 hartrees
Gibbs thermal correction: 1.09753 hartrees

2.7 Calculated Energies of ^{dipp}NDCMg$_2$Me$_2$·4 THF (Triplet):

Electronic energy from SP calculation: −3099.097073 hartrees
Gibbs thermal correction: 1.091803 hartrees

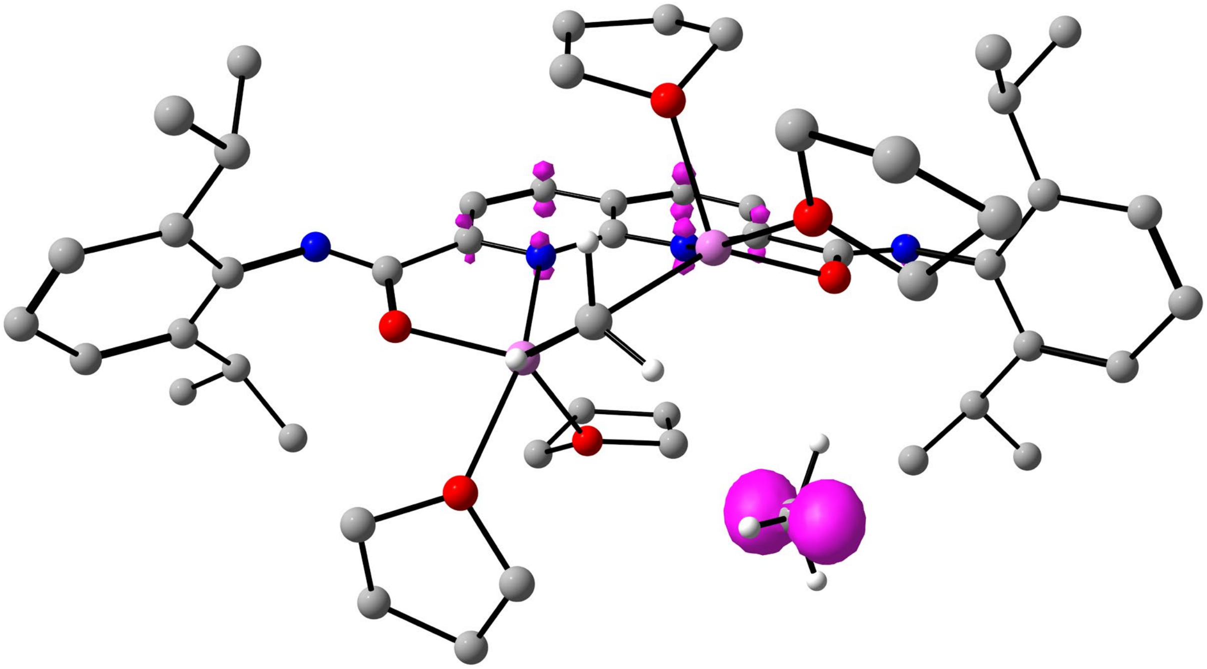

Figure S61: Residual spin density plot of ^{dipp}**NDC**Mg_2Me_2·4 THF (Triplet).

3. X-ray crystal structure determination:

3.1 Crystal structure determination of ^{dipp}NDCK_2·2(18-c-6):

$C_{58}H_{86}K_2N_4O_{14}$ · C_7H_8, Fw = 1233.64, yellow block, 0.28 × 0.18 × 0.09 mm^3, triclinic, P $\overline{1}$ (no. 2), a = 12.8967(7), b = 13.7445(6), c = 19.7939(6) Å, α = 81.917(2), β = 76.556(3), γ = 82.565(2) °, V = 3361.5(3) Å^3, Z = 2, D_x = 1.219 g/cm^3, μ = 0.21 mm^{-1}. The diffraction experiment was performed on a Bruker Kappa ApexII diffractometer with sealed tube and Triumph monochromator (λ = 0.71073 Å) at a temperature of 120(2) K up to a resolution of $(\sin \theta/\lambda)_{max}$ = 0.61 Å^{-1}. The Eval15 software[21] was used for the intensity integration. A multi-scan absorption correction and scaling was performed with SADABS[22] (correction range 0.55-0.75). A total of 45397 reflections was measured, 12523 reflections were unique (R_{int} = 0.084), 6711 reflections were observed [I>2σ(I)]. The structure was solved with Patterson superposition methods using SHELXT.[23] Structure refinement was performed with SHELXL-2019[24] on F^2 of all reflections. Non-hydrogen atoms were refined freely with anisotropic displacement parameters. Hydrogen atoms were introduced in calculated positions and refined with a riding model. Two *i*-propyl groups and one crown-ether ligand were refined with disorder models. 980 Parameters were refined with 2241 restraints (distances, angles and displacement parameters in the disordered groups). R1/wR2 [I > 2σ(I)]: 0.0755 / 0.1951. R1/wR2 [all refl.]: 0.1439 / 0.2335. S = 1.050. Residual electron density between -0.45 and 0.60 e/Å^3. Geometry calculations and checking for higher symmetry was performed with the PLATON program.[25]

3.2 Crystal structure determination of ^{dipp}NDCMg$_2$Cl$_2$·4 THF:

$C_{50}H_{70}Cl_2Mg_2N_4O_6 \cdot C_4H_8O$, Fw = 1014.72, yellow block, 0.25 × 0.10 × 0.07 mm^3, tetragonal, P4$_3$ (no. 78), a = b = 16.9567(5), c = 19.1017(8) Å, V = 5492.3(4) Å^3, Z = 4, D_x = 1.227 g/cm^3, μ = 0.19 mm^{-1}. The diffraction experiment was performed on a Bruker Kappa ApexII diffractometer with sealed tube and Triumph monochromator (λ = 0.71073 Å) at a temperature of 150(2) K up to a resolution of $(\sin \theta/\lambda)_{max}$ = 0.61 Å^{-1}. The Eval15 software was used for the intensity integration.[21] A multi-scan absorption correction and scaling was performed with SADABS (correction range 0.65-0.75).[22] A total of 70786 reflections was measured, 10218 reflections were unique (R_{int} = 0.078), 7493 reflections were observed [$I>2\sigma(I)$]. The structure was solved with Patterson superposition methods using SHELXT.[23] Structure refinement was performed with SHELXL-2018 on F^2 of all reflections.[24] Merohedral twinning was included in the refinement with a twofold rotation about *hkl*=(1,0,0) as twin operation. Non-hydrogen atoms were refined freely with anisotropic displacement parameters. The THF molecules and two of the *i*-propyl groups were refined with a disorder model. The disorder was not fully resolved. Hydrogen atoms were introduced in calculated positions and refined with a riding model. 820 Parameters were refined with 1640 restraints (geometry and displacement parameters of the disordered groups). R1/wR2 [$I > 2\sigma(I)$]: 0.0451 / 0.0967. R1/wR2 [all refl.]: 0.0757 / 0.1089. S = 1.023. Twin fraction BASF = 0.3222(15). Flack parameter[26] x = -0.03(3). Residual electron density between -0.18 and 0.19 e/Å^3. Geometry calculations and checking for higher symmetry was performed with the PLATON program.[25]

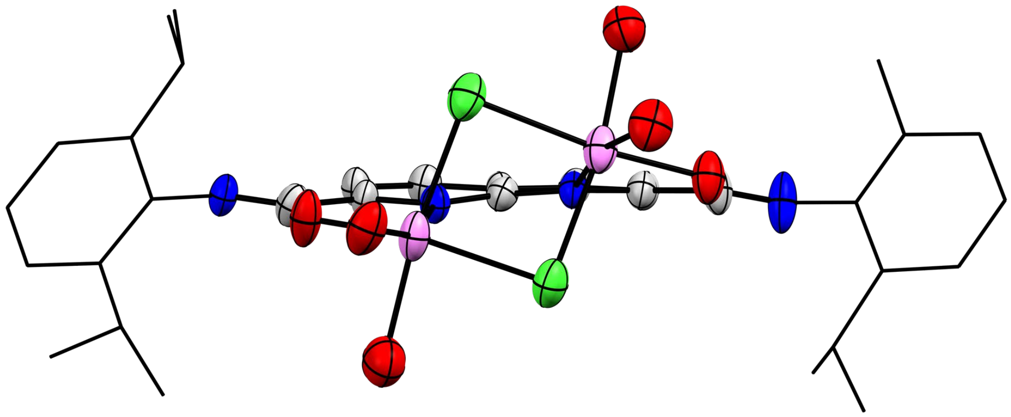

Figure S62: Displacement ellipsoid plot (front view) of the Δ,Δ enantiomer of ^{dipp}**NDC**Mg$_2$Cl$_2$·4 THF at 50% probability. Hydrogen atoms, co-crystallised THF molecules and minor disorder components are omitted for clarity. The dipp substituents are depicted as wireframe for clarity. This view shows the twist observed in the naphthyridine plane and the location of one of the Mg-centres outside of the naphthyridine plane.

3.3 Crystal structure determination of $^{dipp}DAMNMg_2Cl_2 \cdot 2$ THF:

$C_{42}H_{58}Cl_2Mg_2N_4O_2$ + disordered solvent, Fw = 770.44[*], orange needle, 0.56 × 0.12 × 0.06 mm^3, orthorhombic, Pnma (no. 62), a = 15.4268(5), b = 21.1434(8), c = 16.3587(6) Å, V = 5335.8(3) $Å^3$, Z = 4, D_x = 0.959 $g/cm^{3[*]}$, μ = 0.18 $mm^{-1[*]}$. The diffraction experiment was performed on a Bruker Kappa ApexII diffractometer with sealed tube and Triumph monochromator (λ = 0.71073 Å) at a temperature of 150(2) K up to a resolution of $(\sin \theta/\lambda)_{max}$ = 0.61 $Å^{-1}$. The Eval15 software[21] was used for the intensity integration. A multi-scan absorption correction and scaling was performed with SADABS[22] (correction range 0.60-0.75). A total of 42567 reflections was measured, 5125 reflections were unique (R_{int} = 0.076), 2839 reflections were observed [$I>2\sigma(I)$]. The structure was solved with Patterson superposition methods using SHELXT.[23] Structure refinement was performed with SHELXL-2019[24] on F^2 of all reflections. The crystal structure contains voids (1600 $Å^3$ / unit cell) filled with disordered solvent molecules. Their contribution to the structure factors was secured by back-Fourier transformation using the SQUEEZE algorithm[27] resulting in 347 electrons / unit cell. Non-hydrogen atoms were refined freely with anisotropic displacement parameters. The coordinated THF molecule was refined with a disorder model. Hydrogen atoms were introduced in calculated positions and refined with a riding model. 282 Parameters were refined with 112 restraints (geometry and displacement parameters of the disordered THF). R1/wR2 [$I > 2\sigma(I)$]: 0.0635 / 01842. R1/wR2 [all refl.]: 0.1107 / 0.2116. S = 1.044. Residual electron density between -0.33 and 0.33 $e/Å^3$. Geometry calculations and checking for higher symmetry was performed with the PLATON program.[25]

[*] Derived values do not contain the contribution of the disordered solvent molecules.

3.4 Crystal structure determination of $^{dipp}DAMNMg_2(n\text{-}Bu)_2 \cdot 2$ THF:

$C_{50}H_{76}Mg_2N_4O_2 \cdot C_4H_8O$, Fw = 885.87, dark orange needle, 0.69 × 0.13 × 0.10 mm^3, monoclinic, $P2_1/n$ (no. 14), a = 14.4023(9), b = 17.6773(9), c = 20.8713(14) Å, β = 92.020(4)°, V = 5310.4(5) $Å^3$, Z = 4, D_x = 1.108 g/cm^3, μ = 0.09 mm^{-1}. The diffraction experiment was performed on a Bruker Kappa ApexII diffractometer with sealed tube and Triumph monochromator (λ = 0.71073 Å) at a temperature of 150(2) K up to a resolution of $(\sin \theta/\lambda)_{max}$ = 0.61 $Å^{-1}$. The crystal appeared to be twinned with a twofold rotation about *uvw* = [0,0,1] as twin operation. Consequently, two orientation matrices were used for the integration with the Eval15 software,[21] resulting in a HKLF-5 file.[28] A multi-scan absorption correction and scaling was performed with TWINABS[29] (correction range 0.58-0.75). A total of 123089 reflections was measured, 10016 reflections were unique (R_{int} = 0.132), 5936 reflections were observed [$I>2\sigma(I)$]. The structure was solved with Patterson superposition methods using SHELXT.[23] Structure refinement was performed with SHELXL-2018[24] on F^2 of all reflections. Non-hydrogen atoms were refined freely with anisotropic displacement parameters. The coordinated *n*-butyl group and the non-coordinated THF solvent molecule were refined with disorder models. Hydrogen atoms in the ordered part of the structure were located in difference Fourier maps, hydrogen atoms in the disordered part were introduced in calculated positions. All hydrogen atoms were refined with a riding model. 654 Parameters were refined with 366 restraints (geometry and displacement parameters of the disordered *n*-butyl and of the THF molecules). R1/wR2 [$I > 2\sigma(I)$]: 0.0754 / 0.1902. R1/wR2 [all refl.]: 0.1361 / 0.2280. S = 1.018. Twin fraction BASF = 0.486(2). Residual electron density between -0.42 and 0.45 $e/Å^3$. Geometry calculations and checking for higher symmetry was performed with the PLATON program.[25]

CCDC 2422290 (^{dipp}**NDC**K_2·2(18-c-6)), 24222291 (^{dipp}**NDC**Mg_2Cl_2·4 THF), 2422292 (^{dipp}**DAMN**Mg_2Cl_2·2 THF) and 2371205 (^{dipp}**DAMN**Mg_2(*n*-Bu)$_2$·2 THF) contain the supplementary crystallographic data for this paper. These data can be obtained free of charge from The Cambridge Crystallographic Data Centre via www.ccdc.cam.ac.uk/data_request/cif.

4. References

(1) Shields, D. J.; Elkoush, T.; Miura-Stempel, E.; Mak, C. L.; Niu, G.-H.; Gudmundsdottir, A. D.; Campbell, M. G. Visible Light Absorption and Long-Lived Excited States in Dinuclear Silver(I) Complexes with Redox-Active Ligands. *Inorg. Chem.* **2020**, *59* (24), 18338–18344. https://doi.org/10.1021/acs.inorgchem.0c02938.

(2) Behlen, M. J.; Uyeda, C. C2-Symmetric Dinickel Catalysts for Enantioselective [4 + 1]-Cycloadditions. *J. Am. Chem. Soc.* **2020**, *142* (41), 17294–17300. https://doi.org/10.1021/jacs.0c08262.

(3) Doll, J. S.; Regenauer, N. I.; Bothe, V. P.; Wadepohl, H.; Roşca, D.-A. Redox Activity of Iron Diazine-Diimine Carbonyl and Dinitrogen Complexes: A Comparative Study of the Influence of the Heterocyclic Ring. *Inorg. Chem.* **2022**, *61* (1), 520–532. https://doi.org/10.1021/acs.inorgchem.1c03212.

(4) Formanuik, A.; Ortu, F.; Liu, J.; Nodaraki, L. E.; Tuna, F.; Kerridge, A.; Mills, D. P. Double Reduction of 4,4′-Bipyridine and Reductive Coupling of Pyridine by Two Thorium(III) Single-Electron Transfers. *Chem. – Eur. J.* **2017**, *23* (10), 2290–2293. https://doi.org/10.1002/chem.201605974.

(5) Dugan, T. R.; Bill, E.; MacLeod, K. C.; Christian, G. J.; Cowley, R. E.; Brennessel, W. W.; Ye, S.; Neese, F.; Holland, P. L. Reversible C–C Bond Formation between Redox-Active Pyridine Ligands in Iron Complexes. *J. Am. Chem. Soc.* **2012**, *134* (50), 20352–20364. https://doi.org/10.1021/ja305679m.

(6) Blackmore, I. J.; Gibson, V. C.; Hitchcock, P. B.; Rees, C. W.; Williams, D. J.; White, A. J. P. Pyridine N-Alkylation by Lithium, Magnesium, and Zinc Alkyl Reagents: Synthetic, Structural, and Mechanistic Studies on the Bis(Imino)Pyridine System. *J. Am. Chem. Soc.* **2005**, *127* (16), 6012–6020. https://doi.org/10.1021/ja042657g.

(7) Dahlén, A.; Nilsson, Å.; Hilmersson, G. Estimating the Limiting Reducing Power of SmI2/H2O/Amine and YbI2/H2O/Amine by Efficient Reduction of Unsaturated Hydrocarbons. *J. Org. Chem.* **2006**, *71* (4), 1576–1580. https://doi.org/10.1021/jo052268k.

(8) Frisch, M. J.; Trucks, G. W.; Schlegel, H. B.; Scuseria, G. E.; Robb, M. A.; Cheeseman, J. R.; Scalmani, G.; Barone, V.; Petersson, G. A.; Nakatsuji, H.; Li, X.; Caricato, M.; Marenich, A. V.; Bloino, J.; Janesko, B. G.; Gomperts, R.; Mennucci, B.; Hratchian, H. P.; Ortiz, J. V.; Izmaylov, A. F.; Sonnenberg, J. L.; Williams; Ding, F.; Lipparini, F.; Egidi, F.; Goings, J.; Peng, B.; Petrone, A.; Henderson, T.; Ranasinghe, D.; Zakrzewski, V. G.; Gao, J.; Rega, N.; Zheng, G.; Liang, W.; Hada, M.; Ehara, M.; Toyota, K.; Fukuda, R.; Hasegawa, J.; Ishida, M.; Nakajima, T.; Honda, Y.; Kitao, O.; Nakai, H.; Vreven, T.; Throssell, K.; Montgomery Jr., J. A.; Peralta, J. E.; Ogliaro, F.; Bearpark, M. J.; Heyd, J. J.; Brothers, E. N.; Kudin, K. N.; Staroverov, V. N.; Keith, T. A.; Kobayashi, R.; Normand, J.; Raghavachari, K.; Rendell, A. P.; Burant, J. C.; Iyengar, S. S.; Tomasi, J.; Cossi, M.; Millam, J. M.; Klene, M.; Adamo, C.; Cammi, R.; Ochterski, J. W.; Martin, R. L.; Morokuma, K.; Farkas, O.; Foresman, J. B.; Fox, D. J. Gaussian 16 Rev. C.01, Wallingford, CT, (2016).

(9) Frisch, M. J.; Trucks, G. W.; Schlegel, H. B.; Scuseria, G. E.; Robb, M. A.; Cheeseman, J. R.; Scalmani, G.; Barone, V.; Petersson, G. A.; Nakatsuji, H.; Li, X.; Caricato, M.; Marenich, A. V.; Bloino, J.; Janesko, B. G.; Gomperts, R.; Mennucci, B.; Hratchian, H. P.; Ortiz, J. V.; Izmaylov, A. F.; Sonnenberg, J. L.; Williams; Ding, F.; Lipparini, F.; Egidi, F.; Goings, J.; Peng, B.; Petrone, A.; Henderson, T.; Ranasinghe, D.; Zakrzewski, V. G.; Gao, J.; Rega, N.; Zheng, G.; Liang, W.; Hada, M.; Ehara, M.; Toyota, K.; Fukuda, R.; Hasegawa, J.; Ishida, M.; Nakajima, T.; Honda, Y.; Kitao, O.; Nakai, H.; Vreven, T.; Throssell, K.; Montgomery Jr., J. A.; Peralta, J. E.; Ogliaro, F.; Bearpark, M. J.; Heyd, J. J.; Brothers, E. N.; Kudin, K.

N.; Staroverov, V. N.; Keith, T. A.; Kobayashi, R.; Normand, J.; Raghavachari, K.; Rendell, A. P.; Burant, J. C.; Iyengar, S. S.; Tomasi, J.; Cossi, M.; Millam, J. M.; Klene, M.; Adamo, C.; Cammi, R.; Ochterski, J. W.; Martin, R. L.; Morokuma, K.; Farkas, O.; Foresman, J. B.; Fox, D. J. Gaussian 16 Rev. C.02, Wallingford, CT, (2016).

(10) Becke, A. D. Density-functional Thermochemistry. III. The Role of Exact Exchange. *J. Chem. Phys.* **1993**, *98* (7), 5648–5652. https://doi.org/10.1063/1.464913.

(11) Lee, C.; Yang, W.; Parr, R. G. Development of the Colle-Salvetti Correlation-Energy Formula into a Functional of the Electron Density. *Phys. Rev. B* **1988**, *37* (2), 785–789. https://doi.org/10.1103/PhysRevB.37.785.

(12) Ditchfield, R.; Hehre, W. J.; Pople, J. A. Self-Consistent Molecular-Orbital Methods. IX. An Extended Gaussian-Type Basis for Molecular-Orbital Studies of Organic Molecules. *J. Chem. Phys.* **2003**, *54* (2), 724–728. https://doi.org/10.1063/1.1674902.

(13) Francl, M. M.; Pietro, W. J.; Hehre, W. J.; Binkley, J. S.; Gordon, M. S.; DeFrees, D. J.; Pople, J. A. Self-consistent Molecular Orbital Methods. XXIII. A Polarization-type Basis Set for Second-row Elements. *J. Chem. Phys.* **1982**, *77* (7), 3654–3665. https://doi.org/10.1063/1.444267.

(14) Gordon, M. S.; Binkley, J. S.; Pople, J. A.; Pietro, W. J.; Hehre, W. J. Self-Consistent Molecular-Orbital Methods. 22. Small Split-Valence Basis Sets for Second-Row Elements. *J. Am. Chem. Soc.* **1982**, *104* (10), 2797–2803. https://doi.org/10.1021/ja00374a017.

(15) Hariharan, P. C.; Pople, J. A. The Influence of Polarization Functions on Molecular Orbital Hydrogenation Energies. *Theor. Chim. Acta* **1973**, *28* (3), 213–222. https://doi.org/10.1007/BF00533485.

(16) Hehre, W. J.; Ditchfield, R.; Pople, J. A. Self—Consistent Molecular Orbital Methods. XII. Further Extensions of Gaussian—Type Basis Sets for Use in Molecular Orbital Studies of Organic Molecules. *J. Chem. Phys.* **2003**, *56* (5), 2257–2261. https://doi.org/10.1063/1.1677527.

(17) Krishnan, R.; Binkley, J. S.; Seeger, R.; Pople, J. A. Self-consistent Molecular Orbital Methods. XX. A Basis Set for Correlated Wave Functions. *J. Chem. Phys.* **2008**, *72* (1), 650–654. https://doi.org/10.1063/1.438955.

(18) McLean, A. D.; Chandler, G. S. Contracted Gaussian Basis Sets for Molecular Calculations. I. Second Row Atoms, Z=11–18. *J. Chem. Phys.* **2008**, *72* (10), 5639–5648. https://doi.org/10.1063/1.438980.

(19) Grimme, S.; Antony, J.; Ehrlich, S.; Krieg, H. A Consistent and Accurate Ab Initio Parametrization of Density Functional Dispersion Correction (DFT-D) for the 94 Elements H-Pu. *J. Chem. Phys.* **2010**, *132* (15), 154104. https://doi.org/10.1063/1.3382344.

(20) Grimme, S.; Ehrlich, S.; Goerigk, L. Effect of the Damping Function in Dispersion Corrected Density Functional Theory. *J. Comput. Chem.* **2011**, *32* (7), 1456–1465. https://doi.org/10.1002/jcc.21759.

(21) Schreurs, A. M. M.; Xian, X.; Kroon-Batenburg, L. M. J. EVAL15: A Diffraction Data Integration Method Based on Ab Initio Predicted Profiles. *J. Appl. Crystallogr.* **2010**, *43* (1), 70–82. https://doi.org/10.1107/S0021889809043234.

(22) Krause, L.; Herbst-Irmer, R.; Sheldrick, G. M.; Stalke, D. Comparison of Silver and Molybdenum Microfocus X-Ray Sources for Single-Crystal Structure Determination. *J. Appl. Crystallogr.* **2015**, *48* (1), 3–10. https://doi.org/10.1107/S1600576714022985.

(23) Sheldrick, G. M. SHELXT – Integrated Space-Group and Crystal-Structure Determination. *Acta Cryst. A* **2015**, *71* (1), 3–8. https://doi.org/10.1107/S2053273314026370.

(24) Sheldrick, G. M. Crystal Structure Refinement with SHELXL. *Acta Cryst. C* **2015**, *71* (1), 3–8. https://doi.org/10.1107/S2053229614024218.

(25) Spek, A. L. Structure Validation in Chemical Crystallography. *Acta Cryst. D* **2009**, *65* (2), 148–155. https://doi.org/10.1107/S090744490804362X.

(26) Parsons, S.; Flack, H. D.; Wagner, T. Use of Intensity Quotients and Differences in Absolute Structure Refinement. *Acta Cryst. B* **2013**, *69* (3), 249–259. https://doi.org/10.1107/S2052519213010014.

(27) Spek, A. L. *PLATON* SQUEEZE: A Tool for the Calculation of the Disordered Solvent Contribution to the Calculated Structure Factors. *Acta Cryst. C* **2015**, *71* (1), 9–18. https://doi.org/10.1107/S2053229614024929.

(28) Herbst-Irmer, R.; Sheldrick, G. M. Refinement of Twinned Structures with SHELXL97. *Acta Cryst. B* **1998**, *54* (4), 443–449. https://doi.org/10.1107/S0108768197018454.

(29) Sevvana, M.; Ruf, M.; Usón, I.; Sheldrick, G. M.; Herbst-Irmer, R. Non-Merohedral Twinning: From Minerals to Proteins. *Acta Cryst. D* **2019**, *75* (12), 1040–1050. https://doi.org/10.1107/S2059798319010179.
